# 404: Personal data not found — Anonymous vs. account-based internet self-help for social anxiety in a partially randomized patient preference trial

**DOI:** 10.1016/j.invent.2026.100988

**Published:** 2026-08-08

**Authors:** Stefanie Arnold, Michael Vogt, Dajana Šipka, Johanna Böttcher, Friederike Fenski, Malte Elson, Thomas Berger

**Affiliations:** a Department of Clinical Psychology and Psychotherapy, University of Bern, Fabrikstrasse 8, 3012, Bern, Switzerland; b Department of Psychology of Digitalization, University of Bern, Fabrikstrasse 8, 3012, Bern, Switzerland; c Department of Clinical Psychology and Psychotherapy: Psychologische Hochschule Berlin, Am Köllnischen Park 2, 10179, Berlin, Germany

**Keywords:** Internet intervention, Social anxiety, Privacy, Anonymity, Data minimization, Patient preference trial

## Abstract

**Background:**

Privacy and data security concerns are frequently cited barriers to the adoption of internet-based interventions. Allowing anonymous use may reduce these barriers, but whether anonymity affects clinical outcomes is unclear.

**Objective:**

This study compared an internet-based self-help intervention for social anxiety offering platform-level anonymity with a conventional account-based version and investigated whether anonymity preferences were associated with outcomes.

**Methods:**

In this partially randomized patient preference trial, 452 adults with heightened social anxiety symptoms were assigned by randomization or preference to an anonymous or account-based 8-week unguided cognitive behavioral self-help program. Assessments occurred at baseline, post-treatment, and 24-week follow-up. The primary outcome was social anxiety severity, analyzed with mixed-effects models testing superiority of the account-based version.

**Results:**

Participants showed substantial reductions in social anxiety symptoms (within-group d = −0.81 to −0.62). The account-based version was not superior in either arm. The post-treatment between-version difference was small (d = 0.09, 95% CI: −0.17 to 0.35) and robust across sensitivity analyses. Secondary outcomes including depressive symptoms and mental quality of life also improved. Improvements were maintained at follow-up. Most participants preferred the account-based version. Exploratory analyses provided no robust evidence of outcome differences by preference or preference match.

**Discussion:**

The platform-level anonymous intervention was not outperformed by the conventional account-based program. Because the trial was powered for superiority and included no non-active control, formal equivalence and absolute efficacy remain to be established. Within these limits, the findings provide no evidence that the privacy-protective, account-free design came at the cost of reduced symptom improvement.

## Highlights

• Anonymous and account-based intervention both showed social anxiety reductions.
• The account-based version was not superior; the null was robust to sensitivity checks.
• Improvements did not differ by preference match in exploratory analyses.
• Most participants preferred the account-based intervention.

## 1 Introduction

Social anxiety disorder (SAD) is a highly prevalent anxiety disorder associated with substantial social and occupational impairment (Stein and Stein, 2008). Similar to other mental health disorders, many individuals affected by SAD do not seek or receive care. For SAD, however, the treatment gaps are particularly pronounced due to core symptoms such as fear of negative evaluation by others, which hinder engagement with face-to-face treatment (Dalrymple and Zimmerman, 2011). Internet-based interventions have emerged as an important approach to improving access to mental health care (Galderisi et al., 2024). These interventions can deliver evidence-based treatments such as cognitive behavioral therapy in a scalable and flexible format, allowing access at convenient times without direct face-to-face interaction. This makes them particularly well-suited for individuals with SAD, whose core symptoms may make traditional care especially difficult to initiate.

While efficacious internet interventions exist for various psychiatric conditions, including SAD (e.g., Böttcher et al., 2013; Guo et al., 2021), real-world uptake remains low (Jardine et al., 2024). Structural barriers such as limited awareness, poor healthcare integration, and reimbursement issues contribute to this gap (Berardi et al., 2024; Schneider et al., 2025). Beyond these external obstacles, surveys with clinicians and potential users consistently identify privacy and data security concerns as among the most frequently cited barriers to engaging with digital mental health services (Batterham et al., 2023; Berardi et al., 2024; Schneider et al., 2025; Subotic-Kerry et al., 2018). Trust in the organization providing an intervention has also been identified as a relevant factor, with lower institutional trust reducing willingness to engage (Batterham et al., 2023). These concerns are not unfounded. Documented incidents, including the sharing of therapy users' data with third-party advertisers and vulnerabilities in health applications, demonstrate that risks are real (Federal Trade Commission, 2023; Wolfangel, 2023).

The privacy calculus framework offers a useful explanation for how these concerns influence engagement. It proposes that individuals weigh the perceived benefits of using a service against the perceived risks of disclosing personal information, with engagement occurring only when perceived benefits outweigh perceived risks (Kokolakis, 2017). This suggests that intervention design choices that reduce perceived risks, particularly those related to privacy and data disclosure, may be relevant for understanding and improving uptake.

Yet most internet-based interventions rely on conventional account-based systems requiring personally identifiable information such as names, email addresses, and symptom data (Lustgarten et al., 2020), representing a concrete source of perceived risk. The privacy by design framework offers relevant guidance here, proposing that strong privacy protection should be embedded into systems from the outset rather than added retrospectively (Cavoukian, 2009), a principle now reflected in the EU General Data Protection Regulation (GDPR; European Parliament and Council of the European Union, 2016). Applied to internet interventions, this suggests that reducing personal data requirements from the very beginning represents a plausible design strategy to better align interventions with privacy risks and user concerns.

Reduced data collection can be implemented to different degrees. Platforms such as Iterapi, for example, allow participation with only an email address, which can in principle be created for the purpose and not linked to other personal data (Vlaescu et al., 2016). Such pseudonymous designs, however, still generate server-side records of individual usage tied to a persistent identifier. Under the GDPR, pseudonymized data remain personal data (European Parliament and Council of the European Union, 2016), and the burden of achieving unlinkability is shifted to users, presupposing both awareness and digital literacy. Moreover, from a privacy calculus perspective, a registration step may signal identifiability regardless of the data actually stored. The strictest variant of data minimization is anonymity: designing systems in which no personally identifiable information is collected at all, and user activity cannot be linked to individuals. Anonymous interventions may reduce multiple barriers simultaneously by alleviating concerns about data misuse, reducing stigma-related fears, and allowing access without identity disclosure. For individuals with SAD, who may be especially sensitive to evaluation and exposure, these features could be particularly relevant.

Throughout this article, anonymity refers to the intervention platform itself (platform-level anonymity): no account, login, or personally identifiable information is required to use the program, and program use cannot be linked to individuals. Participation in the surrounding research study nonetheless involved standard identifiable procedures (consent, email-based assessments), so the design contrasts an account-free, local-storage workflow with a conventional account-based workflow rather than absolute anonymity.

However, removing identification requirements may also alter core intervention properties. Conventional programs typically use login-based systems to enable centralized data saving or progress tracking, features that may contribute to engagement and treatment effects. Platform-level anonymity thus introduces a potential trade-off between functionality and privacy protection, possibly affecting clinical outcomes, and highlights an empirical gap: would an intervention that protects privacy by forgoing personal data collection be outperformed in terms of clinical outcomes by a conventional, account-based counterpart? For individuals with SAD, who face elevated barriers to care, this question is critical, since a privacy-protective design can only meaningfully improve accessibility if treatment effects are preserved.

From a privacy calculus perspective, users may be expected to favor a format that reduces perceived risks without requiring identity disclosure, though whether this holds when intervention functionality is potentially reduced, and which users are most likely to prefer it, is an open question. Beyond preference itself, whether receiving one's preferred format influences treatment outcomes is a further relevant question, since preference-outcome associations would have direct implications for how anonymous interventions should be implemented and for whom. Prior research suggests that receiving a preferred intervention format is associated with larger treatment effects (Delevry and Le, 2019), but whether this applies to format preferences concerning anonymity specifically has not been examined.

Taken together, platform-level anonymity offers a plausible strategy for reducing privacy-related barriers to engagement but raises questions about clinical outcomes and user preference. To address this, the present partially randomized preference trial evaluated an anonymous version of an internet-based self-help intervention for social anxiety against its conventional account-based counterpart (Berger et al., 2009, Berger et al., 2011; Schulz et al., 2014; Šipka et al., 2025; Stolz et al., 2018). Given that conventional systems include features such as centralized progress tracking that are absent in anonymous designs, a conservative assumption is that removing these may reduce treatment outcomes, motivating a superiority test of the conventional version as the primary research question. Although the broader question concerns comparability of outcomes, a superiority test was chosen as a pragmatic first step. If the conventional version does not outperform the anonymous one, this provides an initial indication that anonymity does not come at the cost of substantially poorer outcomes, justifying further investigation with designs formally powered to test non-inferiority. Secondary questions addressed how many participants preferred the anonymous format, what characteristics distinguished those who did, and whether outcomes were associated with preference and preference match (i.e., whether participants received their preferred or non-preferred version). By examining both outcome and preference data, the study aims to provide an empirical basis for evaluating platform-level anonymity as a viable strategy to improve the accessibility of internet-based interventions for social anxiety.

## 2 Methods

### 2.1 Study design

**Fig. 1:**
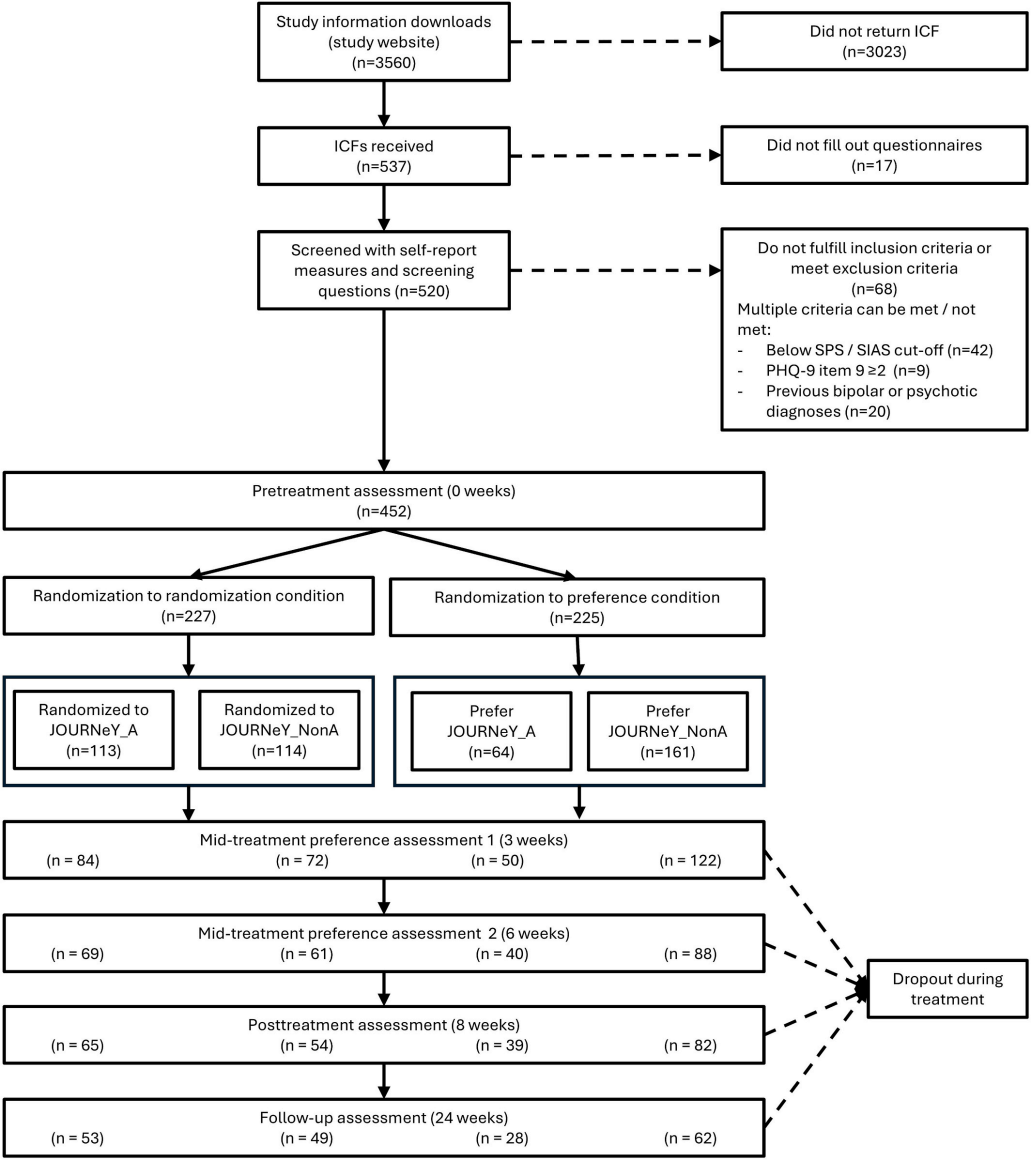
Study flow. Participant flow and assessment timepoints.

This single-center partially randomized patient preference trial is part of the larger study *Preference Research: Investigating Variations of Anonymity, Transparency, and Efficacy in Digital Health Applications* (PRIVATE). A detailed description of the trial and study can be found in the study protocol (Arnold et al., 2025). During the study, participants were assigned to one of two intervention versions: anonymous program (i.e., no login required, no user identification possible) and account-based program (i.e., login required, user data saved, user identification possible). Participants were either assigned according to their preference for one of the program versions or randomly assigned. Data were collected at three timepoints: before the intervention (pre), after the intervention (post), and at follow-up. The overall design is a 2 × 2 × 3 within-between-subjects design with allocation method (randomized vs. preference) and program version (anonymous vs. account-based) as the between-subject factors and timepoint (pre, post, follow-up) as the within-subject factor. Fig. 1 shows the study design and participant flow. The PRIVATE study has been approved by the Ethics Committee of the Canton of Bern, Switzerland on the 30th of May 2024 (2024-00842) and was registered on ClinicalTrials.gov (NCT06465589).

### 2.2 Participants

A total of *n* = 452 participants with heightened social anxiety symptoms were recruited, meeting the pre-specified recruitment target based on the sample size calculation (see 2.8 *Sample Size/Power Analysis*). In the randomized arm, 113 were assigned to the anonymous program while 114 were assigned to the account-based program. In the preference arm, 64 participants chose the anonymous program while 161 chose the account-based program. Candidates were included if they met the following inclusion criteria: 1) be at least 18 years old, 2) sign and return the informed consent form, 3) have access to the internet, 4) have access to a smartphone, PC, or tablet, and 5) exceed pre-defined cutoff scores on at least one of the two SAD measures (>22 points on the Social Phobia Scale [SPS] or >33 points on the Social Interaction Anxiety Scale [SIAS]; Mattick and Clarke, 1998; Stangier et al., 1999). If candidates met the following exclusion criteria, they were excluded: 1) report acute suicidality at the pre-intervention timepoint (≥2 points on suicide item nine of the Patient Health Questionnaire–9 [PHQ-9] (Gräfe et al., 2004; Spitzer, 1999)), or 2) have been diagnosed with bipolar disorder or psychotic symptoms.

### 2.3 Recruitment

The *n* = 452 participants were recruited between the 3rd of September 2024 and the 14th of September 2025 in Switzerland, Germany, Austria, and Liechtenstein through social media (e.g., Instagram, Facebook, TikTok, LinkedIn), Google Ads, interviews in newspapers and magazines as well as forums and websites related to SAD and through the website of the University of Bern. Data were collected from the 3rd of September 2024 to the 2nd of March 2026.

### 2.4 Treatment

#### 2.4.1 Overview

The intervention in the study is the online self-help program JOURNeY which is based on the cognitive behavioral framework for treating SAD by Clark and Wells (1995) and Stangier et al. (2003). The program has demonstrated efficacy compared to control conditions in multiple randomized controlled trials (Berger et al., 2009, Berger et al., 2011; Šipka et al., 2025; Stolz et al., 2018). JOURNeY entails four primary therapeutic components: psychoeducation, cognitive restructuring, attention training, and exposure. The modules, including two supplementary modules, are described below and in further detail in other articles (Arnold et al., 2025; Šipka et al., 2025).

The intervention period lasted for eight weeks, during which participants used the program at their own pace. At the beginning, participants were advised to complete one module per week over six weeks (∼50–60 min each), followed by two weeks of review. Intervention delivery was unguided to ensure equivalence across the two program versions described below and to ensure participants' sense of anonymity. As prior research has shown, unguided interventions can be as efficacious as guided interventions (Berger et al., 2011).

#### 2.4.2 Psychoeducation

This module provided comprehensive, evidence-based information on the mechanisms involved in maintaining social anxiety, including the interaction between negative thoughts, self-focused attention, anxiety, and safety and avoidance behaviors.

#### 2.4.3 Cognitive restructuring

In this module, participants were supported in identifying and modifying maladaptive beliefs contributing to the maintenance of social anxiety through structured exercises.

#### 2.4.4 Attention training

In this module, participants practiced redirecting their focus toward external cues with audio- and video-guided exercises.

#### 2.4.5 Exposure

In this module, participants were instructed to engage in systematic real-life exposure exercises, including the planning, conducting and reflecting of exposure tasks using an exposure diary, with a particular emphasis on reducing safety behaviors.

#### 2.4.6 Additional modules

JOURNeY also included an introductory module with text discussing motivation for changing behaviors as well as a concluding module discussing relapse prevention.

#### 2.4.7 Program versions

Both program versions were delivered via a web-based platform accessible through standard web browsers and contained identical core content. They differed with respect to account creation and the handling of participant-generated content. In the anonymous version (JOURNeY_A), no registration was required; sections involving personal input were provided as a downloadable PDF file that participants completed and stored locally on their devices. In the account-based version (JOURNeY_NonA), participants had to create an account and enter personal reflections directly into the program, allowing for later review. Both versions used cookies solely for essential technical functions (e.g., session management), without tracking user behavior or collecting additional personal data.

As noted in the Introduction, anonymity in JOURNeY_A refers to the intervention platform itself. No personally identifiable information was collected within the program, and usage could not be linked to individual users. Research participation, however, involved standard procedures including written consent and email-based assessments. The conditions therefore reflect a contrast between local-storage-based and account-based intervention workflows rather than absolute anonymity across all study procedures.

### 2.5 Procedures

After giving written consent, candidates were screened for eligibility (see 2.7 *Instruments*). During screening, participants received brief descriptions of both program versions and indicated a preference. Eligible participants were then assigned to a program version using a two-step randomization. First, participants were randomized with equal probability to either a randomization arm or a preference arm. Second, participants in the randomization arm were allocated with equal probability to either the anonymous or account-based program version, independent of stated preference, whereas participants in the preference arm were assigned to their preferred version. This design allowed estimation of between-version effects under randomization as well as exploratory analyses of preference effects.

Assessments were conducted at baseline (pre-assessment), post-intervention (eight weeks), and follow-up (24 weeks). Additional brief assessments occurred in weeks three and six and included preference measures and other measures of the PRIVATE trial not relevant for this study. All measures were self-report questionnaires and distributed automatically via REDCap (Harris et al., 2009, Harris et al., 2019) by email at each timepoint.

To monitor for adverse events, suicidality was assessed using item nine of the Patient Health Questionnaire–9 (PHQ-9) at baseline, post-intervention, and follow-up. If elevated suicidality was reported, the study team contacted the participant to provide crisis resources and encourage help-seeking. Additionally, participants were informed that they could contact the study team at any time in case of distress or to withdraw from the study.

### 2.6 Randomization

The first randomization was conducted using REDCap's built-in randomization tool (Harris et al., 2009, Harris et al., 2019), which is also where all study data were collected. The second randomization was performed by an independent researcher using a list generated with randomly permuted blocks, as REDCap supports only a single randomization per participant. Condition allocation was concealed from investigators.

### 2.7 Instruments/outcomes

#### 2.7.1 Primary outcome

The primary outcome was social anxiety symptom severity at post-treatment (eight weeks), assessed using the Social Phobia Scale (SPS) and the Social Interaction Anxiety Scale (SIAS) (Mattick and Clarke, 1998; Stangier et al., 1999). These widely used self-report measures assess fear of negative evaluation in performance-related (SPS) and interaction-related (SIAS) social situations and have demonstrated good validity and reliability. The German versions show excellent internal consistency (Cronbach α = 0.94 for both scales; Stangier et al., 1999). The internal consistencies at baseline in our sample were good, with α = 0.89 for SPS, and α = 0.86 for SIAS. Each scale comprises 20 items rated on a 5-point Likert scale (0 = not at all to 4 = extremely), with higher scores indicating greater symptom severity. For analyses, a composite score was calculated by averaging the z-standardized SPS and SIAS sum scores, using the pre-treatment mean and pre-treatment standard deviation of the full sample, consistent with recommendations and prior research (Šipka et al., 2025; Song et al., 2013).

#### 2.7.2 Secondary outcomes and other measures

##### 2.7.2.1 Depressive symptoms (Patient Health Questionnaire; PHQ-9)

Depressive symptom severity was assessed using the 9-item Patient Health Questionnaire (PHQ-9), which measures DSM-5 symptoms of major depression on a 4-point Likert scale (American Psychiatric Association, 2013; Gräfe et al., 2004; Spitzer, 1999). Higher scores indicate greater symptom severity. The PHQ-9 has demonstrated good internal consistency with α = 0.88 (Gräfe et al., 2004). Internal consistency in our sample was good with α = 0.81.

##### 2.7.2.2 Quality of life (12-Item Short Form Health Survey; SF-12)

Health-related quality of life was measured using the 12-Item Short Form Health Survey (SF-12), comprising physical and mental health subscales. The SF-12 shows good psychometric properties comparable to the longer 36-item version SF-36 and is widely used as a global quality-of-life measure (Gandek et al., 1998; Ware et al., 1996).

##### 2.7.2.3 Mental illness stigma (Internalized Stigma of Mental Illness Scale; ISMI)

Internalized stigma was assessed using the 29-item Internalized Stigma of Mental Illness Scale (ISMI; Boyd Ritsher et al., 2003; Sibitz et al., 2013), which includes five subscales: alienation, stereotype endorsement, discrimination experience, social withdrawal, and stigma resistance. The scale has demonstrated good internal consistency and test–retest reliability (Sibitz et al., 2013). In line with prior recommendations (Hammer and Toland, 2017), a confirmatory factor analysis was conducted on the full 29-item, five-factor structure to evaluate whether the stigma resistance subscale should be retained in the total score. Factor loadings within the stigma resistance subscale were highly inconsistent: two items loaded strongly (item 26: λ = 0.74; item 27: λ = 0.81), while two loaded negligibly (item 7: λ = 0.21; item 14: λ = 0.09) and one loaded negatively (item 24: λ = −0.16). Internal consistency for the stigma resistance subscale was poor (α = 0.40), compared to acceptable-to-good consistency across the remaining four subscales (α = 0.63–0.84). Given this evidence of internal incoherence, the stigma resistance subscale was excluded from the total score. The four-factor, 24-item model demonstrated adequate fit (CFI = 0.90, TLI = 0.89, RMSEA = 0.058). Internal consistency for the total scale was high with α = 0.92.

##### 2.7.2.4 Help seeking attitudes (Inventory of Attitudes Toward Seeking Mental Health Services; IASMHS)

Help-seeking attitudes regarding mental health services were measured using the 24-item Inventory of Attitudes Toward Seeking Mental Health Services (IASMHS; Kessler et al., 2015; Mackenzie et al., 2004). The instrument has been used in clinical and nonclinical samples, and the German version shows acceptable psychometric properties (Kessler et al., 2015). Internal consistency in our sample was good with α = 0.82.

##### 2.7.2.5 Personality functioning (Level of Personality Functioning Scale–Brief Form 2.0; LPFS-BF)

Personality functioning was assessed with the 12-item Level of Personality Functioning Scale–Brief Form 2.0 (LPFS-BF; Hutsebaut et al., 2016; Spitzer et al., 2021; Weekers et al., 2019), based on the Alternative DSM-5 Model of Personality Disorders (American Psychiatric Association, 2013). Items were rated on a 4-point Likert scale and cover impairments in self- and interpersonal functioning. Validation in clinical samples is ongoing and good internal consistency has been reported for the German version with McDonald's ω ≥ 0.83 (C. Spitzer et al., 2021). In our sample, internal consistency was acceptable with α = 0.79.

##### 2.7.2.6 Preference

Program preference was assessed by presenting participants with descriptions of the two program versions. Participants indicated their preferred version and rated preference strength on a 4-point Likert scale (“How important is this choice to you?” 1 = not important at all to 4 = very important), as done by previous researchers (Lindegaard et al., 2020). A forced choice was required at pre-intervention to allow allocation in the preference arm. At subsequent assessments, participants could indicate no preference (see appendix for translated program descriptions and preference questions).

##### 2.7.2.7 Privacy concerns and protection scale (PCP; Buchanan et al., 2007)

This 28-item self-report measure assesses privacy concerns and behavior. It consists of three subscales: concern, general caution, and technical protection. The concern subscale measures general privacy concern, general caution measures the common-sense steps individuals take to protect their personal information, such as shredding documents or hiding bank card PIN numbers, and technical protection assesses what behaviors participants have previously engaged in to protect their privacy such as deleting cookies. Items were rated on a 5-point Likert scale and internal consistency was acceptable to excellent (concern: α = 0.92; general caution: α = 0.71; technical protection: α = 0.75). Only the baseline measurement of this scale was used to assess differences between participants preferring JOURNeY_A and those preferring JOURNeY_NonA. This measure was not mentioned in the study protocol, and therefore constitutes a deviation from the original analysis plan (Arnold et al., 2025).

##### 2.7.2.8 Engagement

Program engagement was operationalized as the total number of clicks during the intervention period. As individual user tracking was not possible in the anonymous conditions due to the anonymity of the design, engagement was calculated as the average number of clicks per participant for each program version and reported descriptively.

### 2.8 Sample size/power analysis

The study was powered for the primary outcome (composite SPS/SIAS score) and the primary research question comparing social anxiety symptom reduction between program versions in the randomized condition. For the power analysis, superiority of the account-based version was assumed. A target effect size of Cohen's d = 0.35 was specified, with smaller effects considered clinically insignificant. Power calculations were conducted for a time × group interaction with α = 0.05 and 80% power using the ANOVA power Shiny app (Lakens and Caldwell, 2021), based on R (R Foundation for Statistical Computing, 2021) and the Superpower package. The power analysis assumed complete data. Based on 2000 Monte Carlo simulations, *n* = 113 participants per randomized condition were required, yielding *n* = 452 participants across all four conditions (full details in Arnold et al., 2025).

### 2.9 Statistical analysis

Differences in social anxiety symptom changes between JOURNeY_A and JOURNeY_NonA were analyzed using linear mixed-effects models, with post-treatment as the primary endpoint. Time was modeled as a within-subject factor and condition as a between-subject factor. Fixed effects included time, condition, and their interaction, with participant-specific random intercepts to account for individual baseline differences. This approach accounts for correlated observations within participants and allowed estimation of differential change over time (Molenberghs et al., 2004).

Primary analyses, that is, the mixed-effects models, were conducted according to the intention-to-treat (ITT) principle. Estimates for each assessment point were derived from the fitted models. Model assumptions were checked via residual plots and Q-Q plots; no substantial violations were observed. To contextualize the primary result against the pre-specified smallest effect size of interest (d = 0.35; see 2.8), the confidence interval of the between-version difference at post-treatment was additionally examined.

No adjustment for multiple comparisons was applied. The confirmatory conclusion of the trial rests on a single pre-specified primary analysis tested at α = 0.05, and all secondary and preference-related analyses are reported as supportive or exploratory rather than as confirmatory claims. To allow calibrated interpretation, we instead indicate throughout which of these results would not survive correction.

Sensitivity analyses for the primary outcome were conducted using multiple imputation (predictive mean matching, m = 20), applying the same mixed-effects model structure and pooling results across imputed datasets using Rubin's rules. Because both the mixed models and the multiple-imputation sensitivity analysis assume data are missing at random (MAR), we additionally conducted a delta-adjusted (tipping-point) sensitivity analysis for the primary outcome in the randomized arm. For this analysis, the imputation model was enriched with auxiliary variables predictive of the missing values or of missingness itself (the follow-up primary outcome score, mid-treatment measures from the broader PRIVATE trial serving solely as auxiliary predictors, and baseline covariates). Imputed post-treatment values of non-completers in one condition were then shifted toward greater symptom severity in increments of δ = 0.1 standard deviation units (the metric of the composite score, standardized to the pre-treatment mean and standard deviation of the full sample), and the primary model was re-estimated and pooled at each step to identify the smallest departure from MAR at which the time × version interaction would become significant. This procedure was applied to each condition in separate scenarios.

Exploratory analyses examining preference effects used appropriate statistical methods (e.g., mixed models, *t*-tests, or chi-square tests) depending on outcome type. These included comparisons between participants who received their preferred intervention and those who did not. Missing data patterns were reported and considered in interpretation. Effect sizes were calculated to facilitate comparison with other studies. All analyses were conducted using R (Version 4.3.3; R Foundation for Statistical Computing, 2021).

## 3 Results

### 3.1 Participants

The descriptive statistics for the overall sample can be found in Table 1. In line with Consolidated Standards of Reporting Trials (CONSORT) recommendations (Eysenbach and CONSORT-EHEALTH Group, 2011; Moher et al., 2010), no significance tests of baseline differences between randomized conditions are reported, as any such differences are due to chance (de Boer et al., 2015).

**Table 1:** Baseline characteristics of the whole PRIVATE sample.

	Condition	Rand A	Rand NonA	Pref A	Pref NonA	Total
Number of participants	n	113	114	64	161	452
Age	M	35.44	35.35	34.34	34.95	35.09
	SD	11.95	12.15	10.8	10.71	11.38
Sex (n)	Male	38	45	17	66	166
	Female	73	68	46	92	279
	Non-Binary	2	1	1	3	7
Sex detailed (n)	Cis Male	37	44	17	65	163
	Cis Female	73	68	46	92	279
	Trans Male	1	1	0	1	3
	Trans Female	0	0	0	0	0
	Non-Binary	2	1	1	3	7
Relationship Status (n)	Single	37	39	20	49	145
	In a relationship	49	44	27	69	189
	Married	19	26	13	36	94
	Divorced/Separated	7	3	2	5	17
	Widowed	1	2	0	0	3
	Other	0	0	2	2	4
Employment status (n)	Working full-time	47	42	19	62	170
	Working part-time	26	35	24	41	126
	Student (full- or part-time)	28	28	17	39	112
	Unemployed	3	1	3	6	13
	Pensioner	4	4	1	2	11
	Other	5	4	0	11	20
Area of residence (n)	City	59	60	33	76	228
	Suburban	31	33	15	42	121
	Rural	23	21	16	43	103
Pharmacological Treatment (n)	No	96	86	58	121	361
	Yes	17	28	6	40	91
Psychological Treatment (n)	No	78	78	50	116	322
	Yes	35	36	14	45	130
Education (n)	Mandatory Schooling	3	4	3	3	13
	Traineeship	14	21	11	27	73
	University Entrance Qualification	30	28	11	33	102
	Bachelor	27	26	17	38	108
	Master	28	29	17	37	111
	Doctorate or higher	2	2	2	8	14
	Other	9	4	3	15	31
Nationality (n)	Swiss	69	60	35	103	267
	German	33	45	19	47	144
	Austrian	5	2	1	6	14
	Swiss, German	4	1	6	2	13
	Other	2	6	3	3	14
Sexual Orientation (n)	Heterosexual	92	91	53	133	369
	Homosexual	4	9	1	7	21
	Bisexual	8	12	6	15	41
	Pansexual	6	0	3	3	12
	Asexual	1	1	0	2	4
	Other	2	1	1	1	5
Preference Choice	JOURNeY_A	32	29	64	0	125
	JOURNeY_NonA	81	85	0	161	327

Note. Rand A = Randomized to JOURNeY_A; Rand NonA = Randomized to JOURNeY_NonA; Pref A = Assigned to JOURNeY_A because of preference; Pref NonA = Assigned to JOURNeY_NonA because of preference.

Disclaimer: Due to an error in the randomization, one participant too many was assigned to the condition Rand JOURNeY_NonA. Hence, the n in the two randomized conditions are not the same.

In the preference conditions, independent t-tests showed no significant between-group differences except for the subscale self-functioning of the LPFS-BF (t(223) = −2.88, *p* = .004; d = 0.43) where the participants preferring JOURNeY_NonA reported higher impairment. Chi-squared tests revealed no significant between-group differences in the preference arm for most demographics, though differences were found for nationality (χ^2^(4, *N* = 225) = 11.23, *p* = .024), and concurrent pharmacological treatment (χ^2^(1, N = 225) = 6.74, *p* = .009). These baseline comparisons involved a large number of tests without correction for multiple comparisons.

### 3.2 Primary outcome

Results are based on the ITT sample. The primary outcome analysis in the randomized arm was treated as confirmatory. Analyses of the SPS and SIAS, and of the preference arm, are reported as supportive. Estimated means and effect sizes are presented in Table 2. Observed means, fixed effects omnibus tests, and preference strength estimates are in the appendix.

**Table 2:** Primary and secondary outcomes for randomized and preference arms for pre-post analyses and Cohen's d for within-group and between-group effects.

Primary outcome, estimated means						
Condition	Pre-treatment		Post-treatment		Within-group effect size pre - post	Between-group effect size post
	M SD	n	M SD	n	Cohen's d (95% CI)	Cohen's d (95% CI)
SPS						
Rand JOURNeY_A	36.74	113	27.52	113	−0.62	0.07
	13.41		16.16		(−0.89 to −0.35)	
Rand JOURNeY_NonA	38.71	114	28.78	114	−0.64	(−0.19–0.34)
	13.41		17.38		(−0.91 to −0.37)	
Pref JOURNeY_A	34.03	64	23.7	64	−0.76	0.18
	12.55		14.66		(−1.11 to −0.40)	
Pref JOURNeY_NonA	35.96	161	26.45	161	−0.67	(−0.11–0.47)
	12.55		15.61		(−0.90 to −0.45)	

SIAS						
Rand JOURNeY_A	48.32	113	38.72	113	−0.68	0.1
	12.6		15.35		(−0.95 to −0.41)	
Rand JOURNeY_NonA	50.26	114	40.26	114	−0.68	(−0.16–0.36)
	12.6		16.57		(−0.95 to −0.41)	
Pref JOURNeY_A	48.94	64	40.15	64	−0.69	−0.14
	11.74		13.82		(−1.04 to −0.33)	
Pref JOURNeY_NonA	48.22	161	38.06	161	−0.76	(−0.43–0.15)
	11.74		14.75		(−0.99 to −0.54)	

Composite Score						
Rand JOURNeY_A	−0.02	113	−0.79	113	−0.71	0.09
	0.98		1.19		(−0.98 to −0.44)	
Rand JOURNeY_NonA	0.14	114	−0.68	114	−0.72	(−0.17–0.35)
	0.98		1.29		(−0.99 to −0.45)	
Pref JOURNeY_A	−0.10	64	−0.88	64	−0.81	0.01
	0.89		1.04		(−1.17 to −0.45)	
Pref JOURNeY_NonA	−0.05	161	−0.86	161	−0.81	(−0.28–0.31)
	0.89		1.11		(−1.03 to −0.58)	


Secondary outcome, estimated means						
Condition	Pre-treatment		Post-treatment		Within-group effect size pre - post	Between-group effect size post
	M (SD)	n	M (SD)	n	Cohen's d (95% CI)	Cohen's d (95% CI)
PHQ-9						
Rand JOURNeY_A	9.19	113	8.24	113	−0.17	0.11
	5.01		6.07		(−0.43–0.09)	
Rand JOURNeY_NonA	9.89	114	8.95	114	−0.16	(−0.15–0.37)
	5.01		6.54		(−0.42–0.10)	
Pref JOURNeY_A	8.44	64	6.96	64	−0.29	0.15
	4.59		5.44		(−0.64–0.05)	
Pref JOURNeY_NonA	8.77	161	7.80	161	−0.18	(−0.14–0.44)
	4.59		5.83		(−0.40–0.03)	

SF-12, physical						
Rand JOURNeY_A	51.78	113	50.59	113	−0.15	0.12
	7.04		8.76		(−0.41–0.11)	
Rand JOURNeY_NonA	52.93	114	51.71	114	−0.15	(−0.14–0.38)
	7.04		9.44		(−0.41–0.11)	
Pref JOURNeY_A	54.20	64	52.74	64	−0.19	−0.14
	6.92		8.18		(−0.54–0.15)	
Pref JOURNeY_NonA	53.32	161	51.52	161	−0.23	(−0.43–0.15)
	6.92		8.75		(−0.45 to −0.01)	

SF-12, mental						
Rand JOURNeY_A	35.26	113	39.90	113	0.41	−0.06
	9.95		12.50		(0.15–0.67)	
Rand JOURNeY_NonA	34.64	114	39.14	114	0.38	(−0.32–0.20)
	9.95		13.49		(0.12–0.64)	
Pref JOURNeY_A	39.11	64	42.78	64	0.34	−0.19
	9.80		11.57		(−0.01–0.69)	
Pref JOURNeY_NonA	37.66	161	40.53	161	0.26	(−0.48–0.11)
	9.80		12.36		(0.04–0.48)	

ISMI (no resistance subscale)						
Rand JOURNeY_A	1.98	113	1.78	113	−0.38	0.05
	0.49		0.58		(−0.64 to −0.12)	
Rand JOURNeY_NonA	1.97	114	1.81	114	−0.3	(−0.21–0.31)
	0.49		0.61		(−0.56 to −0.04)	
Pref JOURNeY_A	1.84	64	1.70	64	−0.25	0.09
	0.51		0.56		(−0.60–0.10)	
Pref JOURNeY_NonA	1.90	161	1.76	161	−0.26	(−0.20–0.38)
	0.51		0.59		(−0.48 to −0.04)	

IASMHS						
Rand JOURNeY_A	65.32	113	66.76	113	0.11	0.15
	12.26		14.40		(−0.15–0.37)	
Rand JOURNeY_NonA	64.25	114	68.95	114	0.34	(−0.11–0.41)
	12.26		15.27		(0.08–0.60)	
Pref JOURNeY_A	66.30	64	67.40	64	0.09	0.19
	12.19		13.46		(−0.26–0.43)	
Pref JOURNeY_NonA	67.20	161	70.07	161	0.22	(−0.10–0.48)
	12.19		14.05		(−0.001–0.44)	

LPFS-BF						
Rand JOURNeY_A	28.71	113	27.35	113	−0.22	0.15
	5.70		6.69		(−0.48–0.04)	
Rand JOURNeY_NonA	29.46	114	28.36	114	−0.17	(−0.11–0.41)
	5.70		7.13		(−0.43–0.09)	
Pref JOURNeY_A	27.53	64	27.07	64	−0.07	0.09
	5.90		6.58		(−0.42–0.27)	
Pref JOURNeY_NonA	28.61	161	27.67	161	−0.15	(−0.20–0.38)
	5.90		6.84		(−0.37–0.07)	

LPFS-BF self-functioning						
Rand JOURNeY_A	16.23	113	15.19	113	−0.27	0.04
	3.52		4.12		(−0.53 to −0.008)	
Rand JOURNeY_NonA	16.10	114	15.37	114	−0.18	(−0.22–0.30)
	3.52		4.39		(−0.44–0.08)	
Pref JOURNeY_A	14.81	64	14.36	64	−0.12	0.24
	3.41		3.90		(−0.47–0.22)	
Pref JOURNeY_NonA	16.21	161	15.33	161	−0.23	(−0.05–0.53)
	3.41		4.08		(−0.45 to −0.01)	

LPFS-BF interpersonal functioning						
Rand JOURNeY_A	12.48	113	12.15	113	−0.09	0.21
	3.21		3.79		(−0.35–0.17)	
Rand JOURNeY_NonA	13.36	114	13.00	114	−0.1	(−0.05–0.48)
	3.21		4.05		(−0.36–0.16)	
Pref JOURNeY_A	12.72	64	12.71	64	−0.002	−0.11
	3.33		3.80		(−0.35–0.34)	
Pref JOURNeY_NonA	12.40	161	12.28	161	−0.03	(−0.40–0.18)
	3.33		3.97		(−0.25–0.19)	

Note: Standard deviations were derived from model-based standard errors (SD = SE × √n) rather than observed sample standard deviations, as the estimated marginal means from the mixed-effects models do not have directly corresponding observed SDs. Cohen's d was computed from these model-derived pooled SDs. Given the substantial proportion of missing data, observed standard deviations would reflect only completers rather than the full intention-to-treat sample, whereas the model-based approach ensures consistency between reported means and variability estimates. Note that this differs from the conventional computation of Cohen's d using pooled observed standard deviations.

In the randomized conditions, there was a significant effect of time for all outcomes (*F*_*CS*_ (1, 153.54) = 106.94, *p* < .001; *F*_*SPS*_ (1, 143.76) = 88.64, p < .001; *F*_*SIAS*_ (1, 154.16) = 96.93, p < .001) corresponding to medium to large within-group effect sizes (d = −0.72 to −0.62). In the preference conditions, significant improvements over time were similarly observed (*F*_*CS*_ (1, 140.89) = 131.72, p < .001; *F*_*SPS*_ (1, 137.20) = 104.90, p < .001; *F*_*SIAS*_ (1, 144.23) = 102.48, p < .001), corresponding to medium to large within-group effect sizes (d = −0.81 to −0.67). There were no significant time × program version interactions in either arm, and between-group differences within each arm at post-treatment were small (randomized: d = 0.07 to 0.10; preference: d = −0.14 to 0.18).

To contextualize the null interaction against the effect size the trial was designed to detect, we examined the confidence interval of the between-version difference at post-treatment in the randomized arm. For the composite score, the difference was d = 0.09 (95% CI: −0.17 to 0.35). The upper confidence limit thus coincides with the pre-specified smallest effect size of interest (d = 0.35). The data are therefore consistent with the absence of differences the trial was powered to detect, but do not formally exclude an effect at that margin.

Sensitivity analyses in the randomized conditions using multiple imputation yielded consistent results (all interactions *p* > .38). The delta-adjusted (tipping-point) analysis further examined the dependence of this finding on the missing-at-random assumption. Under the auxiliary-enriched imputation model, results at δ = 0 were consistent with the primary analysis (time × version interaction = −0.03, SE = 0.14, *p* = .82). The fraction of missing information for the time × version interaction was 0.29 under the standard imputation model and 0.24 under the auxiliary-enriched model, in both cases well below the proportion of missing post-treatment observations (approximately 48%), reflecting the substantial information about post-treatment scores carried by baseline severity. The interaction became significant only at δ ≥ 0.6, that is, if the unobserved post-treatment scores of non-completers in the anonymous condition were on average at least 0.6 pre-treatment standard deviations more severe than predicted by an imputation model conditioning on their baseline severity, mid-treatment measures, and available follow-up data. A systematic, unidirectional departure of this magnitude (roughly 80% of the average overall pre-to-post improvement) has no empirical support in the observed data, although observed-data comparisons cannot by themselves exclude a missing-not-at-random mechanism. Nonetheless, dropout rates did not differ between program versions and non-completers did not differ at baseline. In the symmetric scenario shifting non-completers in the account-based condition, the interaction likewise became significant only at δ ≥ 0.6 (*p* = .049), now in the direction of superiority of the anonymous version. The primary null finding is thus equally robust to one-sided MAR violations in either condition. Full results are provided in the appendix (J.1–J.4).

Taken together, these results indicate substantial reductions in social anxiety symptoms over time with no evidence of differential change between program versions or allocation methods.

### 3.3 Secondary outcomes

Results for secondary outcomes are presented in Table 2.

In the randomized conditions, significant improvements over time were observed for depressive symptoms (*F*_*PHQ*_ (1, 137.66) = 5.93, *p* = .016), mental quality of life (*F*_*SFmental*_ (1, 153.34) = 28.33, *p* < .001), internalized stigma (*F*_*ISMInr*_ (1, 139.50) = 30.96, p < .001), attitudes toward help-seeking (*F*_*IASMHS*_ (1, 138.05) = 13.70, p < .001) and personality functioning overall (*F*_*LPFS*_ (1, 133.91) = 10.18, *p* = .002) and for the self-functioning subscale (F_LPFSs_ (1, 134.22) = 14.04, p < .001). Physical quality of life showed a small decline (F_SFphysical_ (1, 153.92) = 4.16, p = .04) that would not survive correction for multiple comparisons. Effect sizes were small to moderate (d = −0.38 to 0.41) for the secondary outcomes. No significant time × program version interactions were observed.

In the preference conditions, significant improvements over time were found for depressive symptoms (*F*_*PHQ*_ (1, 134.54) = 10.46, p = .002), mental quality of life (*F*_*SFmental*_ (1, 141.67) = 17.11, p < .001), internalized stigma (*F*_*ISMInr*_ (1, 127.81) = 20.43, p < .001), attitudes toward help-seeking (*F*_*IASMHS*_ (1, 126.31) = 7.81, *p* = .006) and self-functioning (*F*_*LPFSs*_ (1, 135.18) = 8.16, *p* = .005). The effect for overall personality functioning was marginal (*F*_*LPFS*_ (1, 129.52) = 3.92, *p* = .0499). Physical quality of life decreased (*F*_*SFphysical*_ (1, 140.29) = 8.41, *p* = .004). Effect sizes were small (d = −0.29 to 0.34), and no significant time × program version interactions were found. All results of the fixed effects omnibus tests can be found in the appendix. Two participants (0.44% of the whole sample) reported negative effects they attributed to the program and these concerned exhaustion from intervention and influence on relationships and studying.

Secondary outcome analyses were not adjusted for multiple comparisons and are interpreted as supportive rather than confirmatory evidence. Findings with *p*-values close to 0.05 would be the first not to survive such adjustment.

### 3.4 Preference analysis: differences in participant characteristics

Baseline characteristics were compared between participants preferring JOURNeY_A (*n* = 125) and JOURNeY_NonA (*n* = 327), independent of condition assignment. Independent samples *t*-tests showed that participants who preferred JOURNeY_NonA reported significantly higher levels of internalized stigma (t(450) = −2.03, *p* = .043), greater impairment in personality functioning (t(450) = −1.97, p = .049) and greater impairment in self-functioning (t(450) = −2.52, *p* = .012) compared to participants preferring JOURNeY_A. Participants preferring JOURNeY_A additionally reported higher privacy concern at baseline (t(450) = 2.44, *p* = .015, d = 0.26), whereas the general caution and technical protection subscales did not differ significantly. Chi-squared tests indicated significant differences for sex (χ^2^(3, *N* = 452) = 10.04, *p* = .018) and nationality (χ^2^(4, N = 452) = 11.19, *p* = .025). Cis men were more likely to prefer JOURNeY_NonA compared to cis women, trans men and non-binary participants. In addition, Austrian participants were overrepresented among those preferring JOURNeY_NonA. No other baseline differences were observed.

These analyses were exploratory and involved a large number of comparisons without alpha correction. None of the reported differences would remain significant under Bonferroni correction.

### 3.5 Preference match analysis

Further analyses compared preference-matched (*n* = 342) and preference-not-matched (*n* = 110) participants across the full dataset. Table 3 shows estimated means, standard deviations and effect sizes for the preference match analysis. It should be noted that by design, preference-mismatched participants were drawn exclusively from the randomized arm, while preference-matched participants included both randomized and preference arm participants. This structural coupling of match status with allocation method limits causal interpretation of the following analyses, which should be considered exploratory and descriptive.

**Table 3:** Primary and secondary outcomes for preference matched vs. preference not matched arms for pre-post analyses and Cohen's d for within-group and between-group effects.

Primary outcome, estimated means						
Condition Preference Match	Pre-treatment		Post-treatment		Within-group effect size pre - post	Between-group effect size post
	M SD	n	M SD	n	Cohen's d (95% CI)	Cohen's d (95% CI)
SPS						
Match	36.31	342	26.92	342	−0.64	−0.01
	13.05		16.16		(−0.79 to −0.49)	
No Match	37.40	110	26.75	110	−0.74	(−0.23–0.20)
	13.05		15.70		(−1.01 to −0.46)	

SIAS						
Match	48.97	342	39.40	342	−0.69	−0.08
	12.18		15.28		(−0.85 to −0.54)	
No Match	48.52	110	38.16	110	−0.76	(−0.30–0.13)
	12.18		14.83		(−1.04 to −0.49)	

Composite Score						
Match	−0.01	342	−0.79	342	−0.74	−0.05
	0.94		1.17		(−0.89 to −0.58)	
No Match	0.02	110	−0.85	110	−0.83	(−0.27–0.16)
	0.94		1.14		(−1.10 to −0.55)	

Secondary outcome, estimated means						
Condition Preference match	Pre-treatment		Post-treatment		Within-group effect size pre - post	Between-group effect size post
	M (SD)	n	M (SD)	n	Cohen's d (95% CI)	Cohen's d (95% CI)
PHQ-9						
Match	9.15	342	8.21	342	−0.17	−0.1
	4.82		6.05		(−0.32 to −0.02)	
No Match	9.00	110	7.63	110	−0.26	(−0.31–0.12)
	4.82		5.87		(−0.52–0.01)	

SF-12, physical						
Match	53.34	342	51.64	342	−0.21	−0.07
	6.97		8.86		(−0.36 to −0.06)	
No Match	51.77	110	51.03	110	−0.09	(−0.28–0.15)
	6.97		8.64		(−0.36–0.17)	

SF-12, mental						
Match	36.87	342	40.04	342	0.28	0.09
	9.97		12.64		(0.13–0.43)	
No Match	35.38	110	41.27	110	0.53	(−0.12–0.31)
	9.97		12.33		(0.26–0.79)	

ISMI (no resistance subscale)						
Match	1.91	342	1.77	342	−0.25	−0.05
	0.50		0.59		(−0.40 to −0.10)	
No Match	1.99	110	1.74	110	−0.46	(−0.27–0.16)
	0.50		0.58		(−0.73 to −0.19)	

IASMHS						
Match	66.52	342	69.02	342	0.19	−0.15
	12.20		14.41		(0.04–0.34)	
No Match	63.79	110	66.89	110	0.23	(−0.36–0.07)
	12.20		14.15		(−0.03–0.50)	

LPFS-BF						
Match	28.59	342	27.75	342	−0.13	−0.04
	5.81		6.86		(−0.28 to −0.02)	
No Match	29.01	110	27.44	110	−0.25	(−0.26–0.17)
	5.81		6.71		(−0.51–0.02)	

LPFS-BF self-functioning						
Match	15.86	342	15.20	342	−0.17	−0.03
	3.48		4.16		(−0.32 to −0.02)	
No Match	16.38	110	15.08	110	−0.34	(−0.24–0.19)
	3.48		4.07		(−0.61 to −0.08)	

LPFS-BF interpersonal functioning						
Match	12.73	342	12.54	342	−0.05	−0.05
	3.28		3.95		(−0.20–0.10)	
No Match	12.63	110	12.35	110	−0.08	(−0.26–0.17)
	3.28		3.86		(−0.34–0.19)	

Note: Standard deviations were derived from model-based standard errors (SD = SE × √n) rather than observed sample standard deviations, as the estimated marginal means from the mixed-effects models do not have directly corresponding observed SDs. Cohen's d was computed from these model-derived pooled SDs. Given the substantial proportion of missing data, observed standard deviations would reflect only completers rather than the full intention-to-treat sample, whereas the model-based approach ensures consistency between reported means and variability estimates. Note that this differs from the conventional computation of Cohen's d using pooled observed standard deviations.

At baseline, independent t-tests showed small differences between groups only in help-seeking attitudes (t(450) = 2.07, *p* = .039; d = −0.23) and physical quality of life (t(450) = 2.14, *p* = .033; d = −0.24), with preference-matched participants reporting slightly more favorable help-seeking attitudes and higher physical quality of life. Mixed models indicated significant improvements over time in social anxiety symptoms and most secondary outcomes across both groups, corresponding to small-to-large within-group effect sizes. Overall, improvements were similar between groups. Two nominally significant interactions emerged: preference-mismatched participants showed larger reductions in internalized stigma (*F*_*ISMInr*_ (1, 266.14) = 5.47, *p* = .02) with a between-group effect at post of d = −0.05 and larger improvements in mental quality of life (*F*_*SFmental*_ (1, 292.94) = 4.49, *p* = .035) with a between-group effect at post of d = 0.09. However, both effects were small and would not survive correction for multiple comparisons. Subgroup analyses splitting the mismatch group by direction of preference yielded no significant interactions. Given the small subgroup, the exploratory nature of the analyses, and structural coupling between mismatch status and randomized allocation, these results should not be interpreted as strong evidence that outcomes are independent of preference match.

### 3.6 Long-term effects

**Fig. 2:**
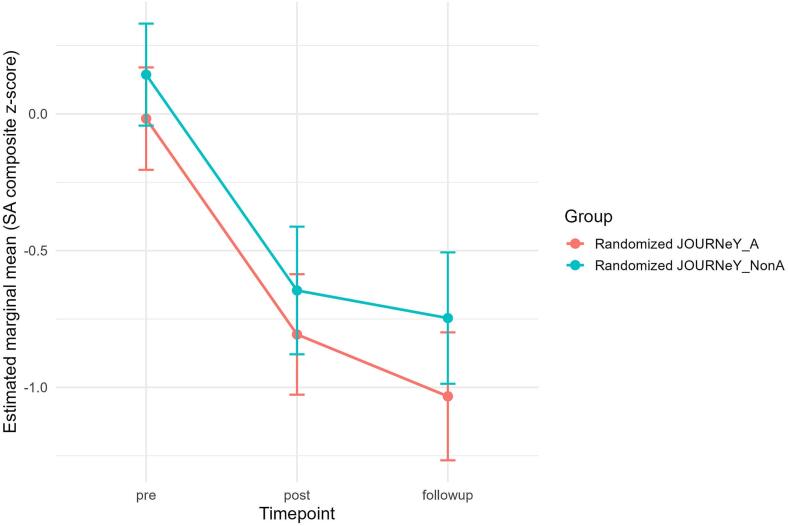
Estimated marginal means of social anxiety (composite z-score) across timepoints in randomized arm. Note: Estimated marginal means of social anxiety composite z-scores at pre, post, and follow-up assessments, derived from a linear mixed-effects model including fixed effects of timepoint, randomized group, and their interaction, with a random intercept for participants. Error bars represent 95% confidence intervals.

**Fig. 3:**
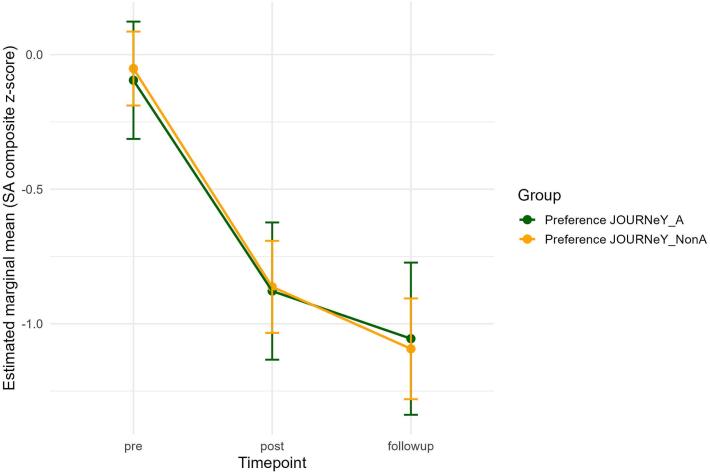
Estimated marginal means of social anxiety (composite z-score) across timepoints in preference arm. Note: Estimated marginal means of social anxiety composite z-scores at pre, post, and follow-up assessments, derived from a linear mixed-effects model including fixed effects of timepoint, preference group, and their interaction, with a random intercept for participants. Error bars represent 95% confidence intervals.

**Fig. 4:**
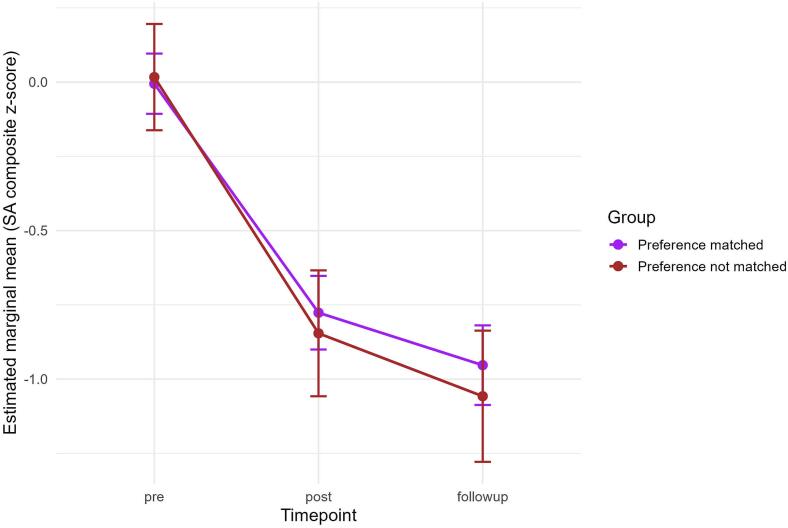
Estimated marginal means of social anxiety (composite z-score) across timepoints in preference matched vs. not matched groups. Note: Estimated marginal means of social anxiety composite z-scores at pre, post, and follow-up assessments, derived from a linear mixed-effects model including fixed effects of timepoint, preference matched vs. not matched group, and their interaction, with a random intercept for participants. Error bars represent 95% confidence intervals.

Fig. 2, Fig. 3, Fig. 4 show the estimated marginal means of the composite social anxiety score across all timepoints; full statistics are in the appendix. Long-term analyses of the primary outcome were pre-specified and are reported as supportive, as are analyses of secondary outcomes. Preference analyses remain exploratory.

There was a significant overall time effect for the randomized conditions (*F*_*CS*_ (2, 250.28) = 103.46, *p* < .001), the preference conditions (*F*_*CS*_ (2, 235.22) = 105.62, *p* < .001), and the preference match analysis (*F*_*CS*_ (2, 487.97) = 190.97, p < .001). Within-group effect sizes from pre to follow-up were large across all analyses (randomized: d = −0.89 to −0.76; preference: d = −0.98 to −0.94; preference match: d = −1.00 to −0.85). Significant time effects with small to moderate effect sizes (d = −0.45 to 0.51) were also observed for depressive symptoms, mental quality of life, internalized stigma, help-seeking attitudes, and personality functioning as well as self-functioning across all analyses. Physical quality of life declined in the preference conditions and preference match analyses. In the randomized arm, a nominally significant time × version interaction emerged for help-seeking attitudes (F_*IASMHS*_ (2, 237.51) = 3.35, p = .04). Significant time × group interactions in the preference match analyses for mental quality of life (*F*_*SFmental*_ (2, 507.88) = 4.04, p = .02), internalized stigma (*F*_*ISMInr*_ (2, 457.21) = 3.28, *p* = .04) and self-functioning (*F*_*LPFSs*_ (2, 466.75) = 3.84, p = .02) indicated larger improvements among preference-mismatched participants. All of these interactions are exploratory, uncorrected, and would not survive correction for multiple comparisons.

### 3.7 Engagement with intervention and study dropouts

Observed means and standard deviations including the n per timepoint and group for each questionnaire can be found in the appendix. Participants using JOURNeY_NonA generated a mean of 160 clicks per participant, whereas participants using JOURNeY_A generated a mean of 138.4 clicks per participant. Because individual user tracking was not possible in JOURNeY_A, these figures are aggregate counts at the respective program level. No inferential comparison between program versions or allocation methods was therefore possible or attempted.

Dropout was defined as missing SPS data, SIAS data, or both at post or follow-up. Completion rates were similar across arms: randomized conditions 52.42% at post and 44.93% at follow-up; preference conditions 53.78% at post and 40.00% at follow-up. Chi-squared tests revealed no significant differences in dropout rates between program versions, allocation methods, or preference-match groups at any timepoint.

## 4 Discussion

Addressing the privacy barriers highlighted in the introduction, the present study compared an internet-based self-help intervention for social anxiety offering platform-level anonymity with its conventional account-based counterpart and examined how user preferences relate to outcomes. Across all arms and conditions, participants showed substantial reductions in social anxiety symptoms with medium to large within-group effect sizes, and the account-based version was not superior in either the randomized or the preference arm. The between-version difference at post-treatment was small, with a confidence interval bounded by the pre-specified smallest effect size of interest, and the primary null finding was robust across sensitivity analyses of the missing data assumptions.

An important constraint on interpretation is that the trial did not include a non-active control condition. The observed within-group improvements (d = −0.62 to −0.81 at post-treatment) therefore cannot be attributed to the intervention alone, as they also reflect regression to the mean, expectancy effects, and the natural course of symptoms in a self-selected, openly recruited sample. This caveat is not merely theoretical. A recent trial of automated interventions for social anxiety reported within-group effects of similar magnitude (d = 0.72–0.79) that did not exceed a waitlist condition, which itself improved by d = 0.54 (Hlynsson et al., 2026). Although previous randomized controlled trials of the same intervention have demonstrated superiority over waitlist controls (Berger et al., 2009, Berger et al., 2011), the present design speaks to the relative outcomes of the two program versions rather than to the absolute efficacy of either. Accordingly, our conclusions concern whether removing account-based features was associated with reduced improvement, not whether the improvement itself was caused by the program.

At least three explanations for the absence of superiority warrant consideration. First, platform-level anonymity may genuinely be compatible with unchanged clinical outcomes. The account-based features removed in the anonymous version (integrated storage and later review of personal entries) may simply not be necessary ingredients of unguided self-help, with core therapeutic content and self-directed practice carrying the improvement. Second, the functional contrast between the versions may have been weaker in practice than by design. The versions differed in a single designed feature (i.e., integrated, account-based storage of personal entries versus local storage via a downloadable PDF) and because usage of the PDF feature could not be monitored, it remains unknown to what extent participants in the anonymous condition engaged with this difference at all. Third, the experienced privacy contrast may have been attenuated by the research context. Perceived anonymity during program use was not assessed, and all participants were identifiable to the study team, so the anonymous version may not have produced a distinctly more private user experience. The present data cannot distinguish between these explanations. The first would support anonymity as a cost-free design choice, whereas the latter two would imply that the contrast tested here was weaker than the conceptual contrast between anonymous and identifiable care. Future studies should therefore include manipulation checks of perceived anonymity during program use and, where possible, objective usage data for both formats.

Symptom reductions were largely maintained at 24-week follow-up and were observed across preference-matched and mismatched groups. These findings are consistent with prior research demonstrating the efficacy of internet-based cognitive behavioral interventions for social anxiety disorder (De Ponti et al., 2024; Kampmann et al., 2016), and the effect sizes fall within the range reported in earlier trials with the same intervention (e.g., Šipka et al., 2025). Improvements were also observed in depressive symptoms, mental quality of life, internalized stigma, help-seeking attitudes, and personality functioning, consistent with evidence that SAD interventions often produce gains across related psychological domains (e.g., Šipka et al., 2025). However, secondary findings should be interpreted with caution, as they were not corrected for multiple comparisons and several effects would not survive such adjustment. The small decline in physical quality of life may reflect temporary discomfort associated with exposure tasks, but given its marginal *p*-value in the randomized conditions and absence in prior trials with the same intervention (Šipka et al., 2025), it should not be over-interpreted.

Beyond the primary comparison, the trial's second aim concerned user preferences, the other side of the privacy calculus: not whether anonymity changes outcomes, but whether users actually want it. Given that data security and anonymity rank among the chief concerns of potential intervention users (Batterham et al., 2023), most participants might have been expected to prefer the anonymous version. The opposite was found: most participants expressed a preference for the account-based version. Notably, preference for the anonymous version was associated with higher self-reported privacy concern at baseline, in line with the privacy calculus framework, suggesting that the anonymity contrast engaged privacy-related motivation at the preference stage. Beyond this, differences between preference groups were small and based on uncorrected comparisons; none would survive Bonferroni correction. These findings do not provide robust evidence that preference for anonymity is associated with a distinct clinical or demographic profile.

Several explanations for the preference pattern are plausible. From a privacy calculus perspective, participants may have perceived the functional benefits of the conventional version, particularly the ability to save entries directly within the program rather than managing them in a separate file, as outweighing the perceived risks of sharing personal data. The context of the study may also be relevant. Since the intervention was developed and provided by a university, baseline trust in the provider may already have been relatively high, making privacy concerns less salient than they might be in other contexts (Batterham et al., 2023). As the present study did not assess participants' reasons for their preferences, these interpretations remain speculative.

The preference distribution must also be interpreted against the selection processes preceding it. All participants had consented to a university-run trial, provided an email address, and accepted repeated email-based assessments. Individuals with the strongest privacy or data security concerns may therefore have declined participation altogether. The enrolled sample is thus likely to underrepresent precisely the privacy-sensitive population for whom anonymous access is intended, and the observed majority preference for the account-based version should not be generalized to potential users outside research contexts. This is underscored by the finding that, even within this self-selected sample, higher privacy concern was associated with preferring the anonymous version. Whether anonymous formats increase reach among non-enrolling, privacy-sensitive individuals is a question this design cannot answer and requires evaluation of publicly deployed programs outside a trial framework.

Finally, contrary to expectations derived from preference trial literature (Delevry and Le, 2019), receiving the preferred intervention format was not associated with larger improvements. Two nominally significant interactions in the pre-post preference match analyses suggested larger improvements among mismatched participants for internalized stigma and mental quality of life. However, these effects were small, exploratory, and would not survive correction for multiple comparisons. Overall, these exploratory findings do not provide clear evidence that format preference or preference match was associated with outcomes in this trial.

### 4.1 Limitations

Several limitations should be considered when interpreting the results. First, a substantial proportion of participants did not provide post-treatment or follow-up data. Although mixed-effects models use all available observations rather than complete cases only, attrition of this magnitude reduces precision and increases the dependence of the estimates on the missing-at-random assumption. Relatedly, the a priori power simulation assumed complete data. Given completion rates of approximately 52% at post-treatment, realized power for the time × version interaction was lower than the planned 80%.The primary null finding should be interpreted with this reduced power in mind, alongside the confidence intervals and sensitivity analyses reported above. Second, the study relied exclusively on self-report measures, which may be influenced by reporting biases. Third, analyses of preference and preference match effects were exploratory, and neither these nor the secondary outcome analyses were corrected for multiple comparisons. Findings should therefore be interpreted accordingly, as several effects would not survive corrections such as Bonferroni adjustment. Fourth, engagement was operationalized using aggregate click counts, a rough proxy for actual program use that permitted no group comparisons due to the anonymous design of JOURNeY_A, and uptake of the PDF journaling feature could not be verified. The strength of the functional contrast between the versions therefore remains uncertain. Fifth, and relatedly, no manipulation check of perceived anonymity was conducted. Although general privacy concern was assessed at baseline and was associated with program preference, we did not measure whether participants in JOURNeY_A actually experienced the program as more private during use. The absence of differential outcomes can therefore not be unambiguously attributed to anonymity being clinically inert, as discussed above. In addition, the psychometric properties of the ISMI warrant caution in interpretation. The preliminary factor analysis suggested that the stigma resistance subscale should be excluded from the total score, and the notably high Cronbach's alpha may reflect item redundancy rather than strong reliability (Tavakol and Dennick, 2011). This scale, especially the German version, therefore requires further psychometric evaluation. Lastly, the whole sample was self-selected and recruited online from the community, limiting generalizability.

Several of these limitations share a common source. Designs that maximize anonymity necessarily minimize what can be observed about their users. The same absence of identifiers that protects participants also prevented individual-level engagement tracking and verification of feature uptake. Reach among individuals unwilling to be identified is limited by a separate constraint, namely the regulatory requirements governing clinical trials (see Section 4.3). Privacy protection and evaluability therefore stand in tension, and studies of anonymous interventions will generally face a trade-off between the strength of the privacy guarantee and the richness of the data available to evaluate it. Methods such as privacy-preserving or aggregated telemetry may ease this tension, but some loss of evidential precision appears intrinsic to genuinely anonymous designs.

### 4.2 Strengths

Despite these limitations, the study has several notable strengths. A key strength of this study is the use of a partially randomized patient preference design, which allowed insight into treatment outcomes under both controlled (randomized) and more naturalistic (preference-based) allocation. Another strength is the direct comparison of two program versions that differed only with respect to platform-level anonymity while maintaining identical therapeutic content. This design allowed a well-controlled comparison of the intended design feature, within the limits noted above. In addition, the study included a relatively large sample, a 24-week follow-up, and analyses with sensitivity checks of the missing data assumptions underlying the primary conclusion.

### 4.3 Implications

The findings have several implications for developing and disseminating internet interventions. First, they demonstrate the feasibility of an account-free, unguided internet-based self-help program that was not outperformed by its conventional counterpart. Delivered via open-access online platforms, such programs can bypass structural barriers such as bureaucratic and reimbursement issues that often limit uptake (Berardi et al., 2024; Schneider et al., 2025). Anonymous access may lower entry thresholds, particularly for those reluctant to seek traditional care due to stigma, evaluation fears, or privacy concerns. Whether this translates into broader community uptake remains to be examined in studies specifically designed to measure real-world access.

Second, and more specifically, the results suggest that strong privacy protections may be implementable in internet interventions without evidence of reduced symptom improvement, within the limits of a superiority design, lending initial support to the feasibility of privacy-centric design approaches, such as privacy by design (Cavoukian, 2009). Notably, the anonymous version relied on a simple downloadable PDF file for user reflections and local storage rather than integrated account-based logging or progress tracking. This suggests that core therapeutic content and self-directed practice may be sufficient, allowing simpler implementations that potentially reduce technical and regulatory complexity. Reducing or eliminating the collection of personally identifiable data may also simplify compliance with data protection requirements, as fewer sensitive data processing procedures are required.

Third, although program version was not associated with differential outcomes, participants nonetheless showed clear preferences, with most favoring the account-based version. This suggests that the accessibility advantages of anonymity may be more relevant for specific subgroups or contexts not fully captured in this trial, such as individuals with elevated privacy concerns. As preference determinants were not directly assessed, future research should examine which intervention characteristics shape format preferences for which user groups, including characteristics such as perceived usability, perceived usefulness, and perceived burden of additional features such as data storage.

Fourth, evaluating anonymous interventions raises methodological demands of its own. Because platform-level anonymity limits what can be observed about program use, future studies will need designs that plan for this trade-off. Aggregated or privacy-preserving telemetry can yield group-level engagement data without re-identifying individuals, and assessments of perceived anonymity during program use, absent here, would clarify whether such designs are experienced as more private rather than merely labeled as such. The question of reach is likewise answerable in principle outside a trial framework, where the regulatory requirements for consent and identification that govern clinical trials do not apply. Aggregate uptake metrics from publicly deployed programs require neither, and could be compared across program versions. Establishing clinical outcomes in this population is harder, since repeated symptom measurement presupposes some form of linkage across timepoints, and individuals who accept such linkage are, by definition, no longer those the anonymous format is intended to reach. The users for whom anonymity matters most are the hardest to observe, and claims about their outcomes will rest on extrapolation from more readily studied samples. Finally, formal non-inferiority designs with predefined margins are needed to establish whether anonymous interventions can be considered clinically equivalent to conventional formats.

### 4.4 Conclusion

The present study showed that an internet intervention for social anxiety offering platform-level anonymity was not outperformed by its conventional account-based counterpart, and this null finding was robust across sensitivity analyses of the missing data assumptions. Because the trial was powered for superiority and included no non-active control condition, formal equivalence and absolute efficacy remain to be established, ideally in a trial designed and powered for non-inferiority. Within these limits, the findings provide no evidence that the privacy-protective, account-free design came at the cost of reduced symptom improvement. Designing interventions that explicitly consider privacy concerns may therefore represent an important step toward improving accessibility, potentially reducing structural barriers and extending the reach of evidence-based care to those who might otherwise not seek it.

## Declaration of Generative AI and AI-assisted technologies in the writing process

During the preparation of this work the authors used Claude AI in order to improve clarity and readability of writing style as well as linking sentences and words connecting paragraphs. After using this tool, the authors reviewed and edited the content as needed and take full responsibility for the content of the published article.

## Funding

The study was funded by the 10.13039/100009068University of Bern (Digitalization Commission DigiK) as part of the strategy “Humans in Digital Transformation” given to Prof. Dr. Thomas Berger and Prof. Dr. Malte Elson. The funding source had no role in the design of the study, data collection, analysis, interpretation, writing of the manuscript nor in the decision to submit this report.

## Declaration of competing interest

The authors declare that they have no known competing financial interests or personal relationships that could have appeared to influence the work reported in this paper.

## Data Availability

The anonymized data that support the findings of this study are available on OSF at https://osf.io/njb3z. The full data are not publicly available due to the sensitive nature of mental health data and the need to protect participant privacy. More of the data may be available on request from the last author, TB.
