# Supplementary material for: 404: Personal data not found — Anonymous vs. account-based internet self-help for social anxiety in a partially randomized patient preference trial
Source: Internet Interv. 2026 Aug 8;45:100988. doi: 10.1016/j.invent.2026.100988 (PMC13476740; doi:10.1016/j.invent.2026.100988)
Supplement: Supplementary file 1 [file mmc1.docx]

Appendix A

Texts used to ask participant’s preferences including the answer possibilities

Version 1: To use the online program, you do not need to create an account for this version, i.e., you do not need to provide an email address or password. You can access the program directly via any browser and use it completely anonymously. In individual modules of the program, there are sections in which you are asked to write down your personal thoughts and experiences. Furthermore, some exercises ask you to complete the exercises and then record your experiences. To do this, you will be given the option at the beginning to download a PDF file where you can write down these thoughts and experiences and save them locally on your computer, so that no personal data is transmitted and stored on our server.

Version 2: In this version, you must first enter an email address and set a password to create an account so that you can use the program. This has the advantage that every time you log in, you can continue right where you left off last time. In addition, there are sections and exercises in individual modules and pages of the program in which you are asked to answer questions and write down your personal thoughts and experiences. In this version, you can write everything down directly in the program and access it again at any time. However, data is not stored completely anonymously here, but in connection with your account or the email address you provided when setting up your account.

Baseline questions with radiobuttons:

After reading these descriptions, which program do you prefer? Which version would you rather work with?

○ Version 1

○ Version 2

How important is this choice to you?

○ 1, not at all important

○ 2,

○ 3,

○ 4, very important

Subsequent measurement point questions with radiobuttons:

After reading these descriptions, which program do you prefer? Which version would you rather work with?

○ 0, Version 1

○ 1, Version 2

○ 99, Unsure / undecided

How important is this choice to you?

○ 1, not at all important

○ 2,

○ 3,

○ 4, very important

Appendix B.1

Observed means and standard deviations for primary outcomes at all timepoints across all arms

| Primary Outcome - Observed | | | | | | |
| --- | --- | --- | --- | --- | --- | --- |
|  | Pre-Treatment | | Post-Treatment | | Follow-Up | |
|  | M  SD | n | M  SD | n | M  SD | n |
|  | SPS | | | | | |
| Rand A | 36.74 | 113 | 28.23 | 65 | 23.34 | 53 |
|  | 12.03 |  | 13.93 |  | 13.17 |  |
| Rand NonA | 38.71 | 114 | 28.48 | 54 | 27.76 | 49 |
|  | 13.46 |  | 15.46 |  | 15.45 |  |
| Pref A | 34.03 | 64 | 23.10 | 39 | 21.34 | 28 |
|  | 12.56 |  | 11.98 |  | 11.67 |  |
| Pref NonA | 35.96 | 161 | 25.83 | 82 | 22.19 | 62 |
|  | 13.12 |  | 11.42 |  | 11.15 |  |
|  | SIAS | | | | | |
| Rand A | 48.32 | 113 | 39.69 | 65 | 35.57 | 53 |
|  | 11.53 |  | 14.30 |  | 14.86 |  |
| Rand NonA | 50.26 | 114 | 40.48 | 54 | 40.00 | 49 |
|  | 11.59 |  | 15.31 |  | 15.48 |  |
| Pref A | 48.93 | 64 | 39.79 | 39 | 36.00 | 28 |
|  | 11.16 |  | 12.50 |  | 12.17 |  |
| Pref NonA | 48.22 | 161 | 37.70 | 82 | 35.39 | 62 |
|  | 11.51 |  | 12.52 |  | 12.26 |  |
|  | Composite Score SA | | | | | |
| Rand A | -0.02 | 113 | -0.72 | 65 | -1.09 | 53 |
|  | 0.86 |  | 1.10 |  | 1.10 |  |
| Rand NonA | 0.14 | 114 | -0.68 | 54 | -0.73 | 49 |
|  | 0.94 |  | 1.20 |  | 1.21 |  |
| Pref A | -0.1 | 64 | -0.92 | 39 | -1.13 | 28 |
|  | 0.86 |  | 0.92 |  | 0.92 |  |
| Pref NonA | -0.05 | 161 | -0.90 | 82 | -1.14 | 62 |
|  | 0.89 |  | 0.89 |  | 0.88 |  |

Note: Rand A = randomized to anonymous version; Rand NonA = randomized to non-anonymous version; Pref A = prefer and assigned to anonymous version; Pref NonA = prefer and assigned to non-anonymous version

Appendix B.2

Observed means and standard deviations for secondary outcomes at all timepoints across all arms

| Secondary Outcomes - Observed | | | | | | |
| --- | --- | --- | --- | --- | --- | --- |
|  | Pre-Treatment | | Post-Treatment | | Follow-Up | |
|  | M  SD | n | M  SD | n | M  SD | n |
|  | PHQ-9 | | | | | |
| Rand A | 9.19 | 113 | 7.95 | 65 | 7.17 | 52 |
|  | 4.58 |  | 4.57 |  | 5.39 |  |
| Rand NonA | 9.89 | 114 | 8.30 | 54 | 9.06 | 49 |
|  | 5.28 |  | 5.49 |  | 5.75 |  |
| Pref A | 8.44 | 64 | 6.69 | 39 | 7.96 | 28 |
|  | 4.45 |  | 3.76 |  | 5.68 |  |
| Pref NonA | 8.77 | 161 | 7.38 | 82 | 6.95 | 62 |
|  | 4.65 |  | 4.70 |  | 5.28 |  |
|  | SF12 physical | | | | | |
| Rand A | 51.78 | 113 | 50.66 | 64 | 52.05 | 52 |
|  | 7.01 |  | 7.29 |  | 6.83 |  |
| Rand NonA | 52.93 | 114 | 51.99 | 54 | 51.27 | 49 |
|  | 6.64 |  | 7.79 |  | 8.09 |  |
| Pref A | 54.2 | 64 | 52.18 | 39 | 53.59 | 28 |
|  | 5.31 |  | 6.54 |  | 6.06 |  |
| Pref NonA | 53.32 | 161 | 51.32 | 82 | 52.25 | 62 |
|  | 6.95 |  | 8.00 |  | 7.61 |  |
|  |  |  |  |  |  |  |
|  | SF12 mental | | | | | |
| Rand A | 35.26 | 113 | 40.21 | 64 | 40.05 | 52 |
|  | 9.34 |  | 9.76 |  | 12.07 |  |
| Rand NonA | 34.64 | 114 | 39.49 | 54 | 37.53 | 49 |
|  | 9.4 |  | 12.37 |  | 11.99 |  |
| Pref A | 39.11 | 64 | 43.99 | 39 | 42.17 | 28 |
|  | 9.4 |  | 9.55 |  | 11.30 |  |
| Pref NonA | 37.66 | 161 | 40.67 | 82 | 41.71 | 62 |
|  | 9.79 |  | 10.27 |  | 11.05 |  |
|  |  |  |  |  |  |  |
|  | ISMI NoRes | | | | | |
| Rand A | 1.98 | 113 | 1.81 | 64 | 1.75 | 51 |
|  | 0.48 |  | 0.51 |  | 0.51 |  |
| Rand NonA | 1.97 | 114 | 1.80 | 54 | 1.88 | 49 |
|  | 0.48 |  | 0.57 |  | 0.49 |  |
| Pref A | 1.84 | 64 | 1.76 | 39 | 1.71 | 27 |
|  | 0.48 |  | 0.57 |  | 0.50 |  |
| Pref NonA | 1.9 | 161 | 1.71 | 82 | 1.68 | 61 |
|  | 0.51 |  | 0.46 |  | 0.47 |  |
|  |  |  |  |  |  |  |
|  | IASMHS | | | | | |
| Rand A | 65.32 | 113 | 66.84 | 64 | 69.88 | 51 |
|  | 11.91 |  | 12.31 |  | 12.44 |  |
| Rand NonA | 64.25 | 114 | 68.24 | 54 | 65.85 | 48 |
|  | 11.78 |  | 14.40 |  | 14.14 |  |
| Pref A | 66.3 | 64 | 65.72 | 39 | 68.33 | 27 |
|  | 13.33 |  | 13.11 |  | 13.97 |  |
| Pref NonA | 67.2 | 161 | 71.38 | 82 | 72.69 | 61 |
|  | 11.88 |  | 10.53 |  | 11.36 |  |
|  |  |  |  |  |  |  |
|  | LPFS total | | | | | |
| Rand A | 28.71 | 113 | 27.06 | 64 | 25.63 | 51 |
|  | 5.25 |  | 6.07 |  | 6.25 |  |
| Rand NonA | 29.46 | 114 | 28.42 | 53 | 28.69 | 48 |
|  | 5.64 |  | 6.29 |  | 5.76 |  |
| Pref A | 27.53 | 64 | 27.21 | 38 | 26.59 | 27 |
|  | 6.03 |  | 6.26 |  | 6.38 |  |
| Pref NonA | 28.61 | 161 | 26.95 | 82 | 26.80 | 61 |
|  | 5.74 |  | 5.99 |  | 6.15 |  |
|  |  |  |  |  |  |  |
|  | LPFS - self | | | | | |
| Rand A | 16.23 | 113 | 14.94 | 64 | 14.04 | 51 |
|  | 3.33 |  | 3.62 |  | 3.86 |  |
| Rand NonA | 16.10 | 114 | 15.43 | 53 | 15.52 | 48 |
|  | 3.54 |  | 3.80 |  | 3.88 |  |
| Pref A | 14.81 | 64 | 14.13 | 38 | 14.04 | 27 |
|  | 3.42 |  | 3.63 |  | 3.51 |  |
| Pref NonA | 16.21 | 161 | 15.05 | 82 | 15.03 | 61 |
|  | 3.23 |  | 3.75 |  | 3.67 |  |
|  |  |  |  |  |  |  |
|  | LPFS - interpersonal | | | | | |
| Rand A | 12.48 | 113 | 12.13 | 64 | 11.59 | 51 |
|  | 2.86 |  | 3.22 |  | 3.09 |  |
| Rand NonA | 13.36 | 114 | 12.98 | 53 | 13.17 | 48 |
|  | 3.37 |  | 3.57 |  | 3.13 |  |
| Pref A | 12.72 | 64 | 13.08 | 38 | 12.56 | 27 |
|  | 3.41 |  | 3.38 |  | 3.46 |  |
| Pref NonA | 12.40 | 161 | 11.90 | 82 | 11.77 | 61 |
|  | 3.32 |  | 3.29 |  | 3.52 |  |
|  |  |  |  |  |  |  |
|  | PCP Concerns | | | | | |
| Rand A | 42.61 | 113 |  |  |  |  |
|  | 12.85 |  |  |  |  |  |
| Rand NonA | 43.80 | 114 |  |  |  |  |
|  | 13.11 |  |  |  |  |  |
| Pref A | 43.05 | 64 |  |  |  |  |
|  | 13.24 |  |  |  |  |  |
| Pref NonA | 39.55 | 161 |  |  |  |  |
|  | 11.78 |  |  |  |  |  |
|  |  |  |  |  |  |  |
|  | PCP General Caution | | | | | |
| Rand A | 13.41 | 113 |  |  |  |  |
|  | 4.60 |  |  |  |  |  |
| Rand NonA | 13.76 | 114 |  |  |  |  |
|  | 4.54 |  |  |  |  |  |
| Pref A | 13.59 | 64 |  |  |  |  |
|  | 4.45 |  |  |  |  |  |
| Pref NonA | 13.21 | 161 |  |  |  |  |
|  | 4.42 |  |  |  |  |  |
|  |  |  |  |  |  |  |
|  | PCP Technical Protection | | | | | |
| Rand A | 18.89 | 113 |  |  |  |  |
|  | 5.53 |  |  |  |  |  |
| Rand NonA | 19.18 | 114 |  |  |  |  |
|  | 5.05 |  |  |  |  |  |
| Pref A | 19.28 | 64 |  |  |  |  |
|  | 4.76 |  |  |  |  |  |
| Pref NonA | 19.25 | 161 |  |  |  |  |
|  | 5.23 |  |  |  |  |  |

Note: Rand A = randomized to anonymous version; Rand NonA = randomized to non-anonymous version; Pref A = prefer and assigned to anonymous version; Pref NonA = prefer and assigned to non-anonymous version

Appendix B.3

Observed preference strength for all arms at all timepoints

|  | Preference Strength | | | | | | | | | | | | | | | | |
| --- | --- | --- | --- | --- | --- | --- | --- | --- | --- | --- | --- | --- | --- | --- | --- | --- | --- |
|  |  | | Pre-  Treatment |  | Mid1-  Treatment | |  | Mid2-  Treatment | |  | | Post-  Treatment | |  | Follow-up  Treatment | |  |
|  |  | | M  SD | n | M  SD | | n | M  SD | | n | | M  SD | | n | M  SD | | n |
| Rand A | | 2.36 | | 113 | | 2.43 | 84 | | 2.45 | | 69 | 2.39 | 64 | | 2.14 | 50 | |
|  | | 0.92 | |  |  | 0.97 |  |  | 0.93 | |  | 0.94 |  |  | 0.83 |  |  |
| Rand NonA | | 2.62 | | 114 | | 2.82 | 72 | | 2.69 | | 61 | 2.6 | 53 | | 2.64 | 47 | |
|  | | 0.88 | |  |  | 0.84 |  |  | 0.87 | |  | 0.97 |  |  | 0.92 |  |  |
| Pref A | | 2.42 | | 64 | | 2.68 | 50 | | 2.58 | | 40 | 2.66 | 38 | | 2.63 | 27 | |
|  | | 0.83 | |  |  | 0.84 |  |  | 0.87 | |  | 0.97 |  |  | 0.97 |  |  |
| Pref NonA | | 2.45 | | 161 | | 2.72 | 122 | | 2.63 | | 88 | 2.72 | 81 | | 2.56 | 61 | |
|  | | 0.86 | |  |  | 0.89 |  |  | 0.9 | |  | 0.99 |  |  | 0.92 |  |  |

Note: Rand A = randomized to anonymous version; Rand NonA = randomized to non-anonymous version; Pref A = prefer and assigned to anonymous version; Pref NonA = prefer and assigned to non-anonymous version

Appendix B.4

Observed means and standard deviations for primary and secondary outcomes at all timepoints across

for whole sample split by preference match.

| Primary Outcome - Observed | | | | | | |
| --- | --- | --- | --- | --- | --- | --- |
|  | Pre-Treatment | | Post-Treatment | | Follow-Up | |
|  | M  SD | n | M  SD | n | M  SD | n |
|  | SPS | | | | | |
| Preference matched | 36.31 | 342 | 26.25 | 178 | 23.88 | 138 |
|  | 12.98 |  | 12.23 |  | 12.30 |  |
| Preference not matched | 37.4 | 110 | 27.74 | 62 | 23.87 | 54 |
|  | 12.72 |  | 15.91 |  | 15.08 |  |
|  | SIAS | | | | | |
| Preference matched | 48.97 | 342 | 39.17 | 178 | 37.46 | 138 |
|  | 11.43 |  | 12.97 |  | 12.91 |  |
| Preference not matched | 48.52 | 110 | 39.29 | 62 | 34.76 | 54 |
|  | 11.68 |  | 15.51 |  | 16.12 |  |
|  | Composite score | | | | | |
| Preference matched | -0.01 | 342 | -0.82 | 178 | -0.99 | 138 |
|  | 0.89 |  | 0.95 |  | 0.97 |  |
| Preference not matched | 0.02 | 110 | -0.76 | 62 | -1.11 | 54 |
|  | 0.89 |  | 1.24 |  | 1.23 |  |
| Secondary Outcomes | | | | | | |
|  | Pre-Treatment | | Post-Treatment | | Follow-Up | |
|  | M  SD | n | M  SD | n | M  SD | n |
|  | PHQ-9 | | | | | |
| Preference matched | 9.15 | 342 | 7.72 | 178 | 8.17 | 138 |
|  | 4.83 |  | 4.91 |  | 5.70 |  |
| Preference not matched | 9 | 110 | 7.37 | 62 | 6.49 | 53 |
|  | 4.66 |  | 4.17 |  | 4.87 |  |
|  | SF12 physical | | | | | |
| Preference matched | 53.34 | 342 | 51.33 | 178 | 52.48 | 138 |
|  | 6.49 |  | 7.95 |  | 7.51 |  |
| Preference not matched | 51.77 | 110 | 51.75 | 61 | 51.28 | 53 |
|  | 7.22 |  | 6.11 |  | 6.75 |  |
|  | SF12 mental | | | | | |
| Preference matched | 36.87 | 342 | 40.71 | 178 | 39.65 | 138 |
|  | 9.71 |  | 10.64 |  | 11.71 |  |
| Preference not matched | 35.38 | 110 | 41.16 | 61 | 41.83 | 53 |
|  | 9.35 |  | 10.48 |  | 11.49 |  |
|  | ISMI NoRes | | | | | |
| Preference matched | 1.91 | 342 | 1.75 | 178 | 1.76 | 136 |
|  | 0.49 |  | 0.52 |  | 0.51 |  |
| Preference not matched | 1.99 | 110 | 1.79 | 61 | 1.74 | 52 |
|  | 0.5 |  | 0.53 |  | 0.45 |  |
|  | IASMHS | | | | | |
| Preference matched | 66.52 | 342 | 69.04 | 178 | 69.58 | 136 |
|  | 12.11 |  | 12.06 |  | 13.20 |  |
| Preference not matched | 63.79 | 110 | 67.03 | 61 | 69.43 | 51 |
|  | 11.86 |  | 13.7 |  | 12.44 |  |
|  | LPFS total | | | | | |
| Preference matched | 28.59 | 342 | 27.42 | 176 | 27.40 | 136 |
|  | 5.78 |  | 6.01 |  | 6.03 |  |
| Preference not matched | 29.01 | 110 | 27.15 | 61 | 25.69 | 51 |
|  | 5.25 |  | 6.42 |  | 6.42 |  |
|  | LPFS self | | | | | |
| Preference matched | 15.86 | 342 | 14.97 | 176 | 15.00 | 136 |
|  | 3.42 |  | 3.68 |  | 3.61 |  |
| Preference not matched | 16.38 | 110 | 14.93 | 61 | 14.06 | 51 |
|  | 3.25 |  | 3.8 |  | 4.16 |  |
|  | LPFS interpersonal | | | | | |
| Preference matched | 12.73 | 342 | 12.45 | 176 | 12.40 | 136 |
|  | 3.37 |  | 3.34 |  | 3.44 |  |
| Preference not matched | 12.63 | 110 | 12.21 | 61 | 11.63 | 51 |
|  | 2.89 |  | 3.46 |  | 3.01 |  |
|  | PCP Concerns | | | | | |
| Preference matched | 41.25 | 342 |  |  |  |  |
|  | 12.52 |  |  |  |  |  |
| Preference not matched | 43.84 | 110 |  |  |  |  |
|  | 13.05 |  |  |  |  |  |
|  | PCP General Caution | | | | | |
| Preference matched | 13.33 | 342 |  |  |  |  |
|  | 4.48 |  |  |  |  |  |
| Preference not matched | 13.84 | 110 |  |  |  |  |
|  | 4.51 |  |  |  |  |  |
|  | PCP Technical Protection | | | | | |
| Preference matched | 19.15 | 342 |  |  |  |  |
|  | 5.19 |  |  |  |  |  |
| Preference not matched | 19.16 | 110 |  |  |  |  |
|  | 5.20 |  |  |  |  |  |

Appendix B.5

Observed means and standard deviations for preference strength for all timepoints for whole sample split by preference match.

| Preference Strength | | | | | | | | | |  |
| --- | --- | --- | --- | --- | --- | --- | --- | --- | --- | --- |
|  | Pre-  Treatment | | Mid1-  Treatment | | Mid2-  Treatment | | Post-  Treatment | | Follow-up  Treatment | |
|  | M  SD | n | M  SD | n | M  SD | n | M  SD | n | M  SD | n |
|  | Preference Strength | | | | | | | | | |
| Preference matched | 2.47 | 342 | 2.74 | 250 | 2.65 | 191 | 2.7 | 175 | 2.61 | 135 |
|  | 0.86 |  | 0.88 |  | 0.90 |  | 0.97 |  | 0.92 |  |
| Preference not matched | 2.46 | 110 | 2.40 | 78 | 2.39 | 67 | 2.3 | 61 | 2.12 | 50 |
|  | 0.93 |  | 0.93 |  | 0.85 |  | 0.92 |  | 0.82 |  |

Appendix B.6

Observed means and standard deviations for primary and secondary outcomes at all timepoints across

for whole sample split by baseline preference indication

| Primary Outcome - Observed | | | | | | |
| --- | --- | --- | --- | --- | --- | --- |
|  | Pre-Treatment | | Post-Treatment | | Follow-Up | |
|  | M  SD | n | M  SD | n | M  SD | n |
|  | SPS | | | | | |
| Prefer JOURNeY_A | 35.33 | 125 | 25.32 | 74 | 23.68 | 59 |
|  | 12.39 |  | 12.85 |  | 13.67 |  |
| Prefer JOURNeY_NonA | 37.05 | 327 | 27.22 | 166 | 23.96 | 133 |
|  | 13.09 |  | 13.43 |  | 12.90 |  |
|  | SIAS | | | | | |
| Prefer JOURNeY_A | 48.73 | 125 | 39.05 | 74 | 35.80 | 59 |
|  | 10.73 |  | 14.72 |  | 14.35 |  |
| Prefer JOURNeY_NonA | 48.91 | 327 | 39.27 | 166 | 37.11 | 133 |
|  | 11.77 |  | 13.17 |  | 13.72 |  |
|  | Composite score | | | | | |
| Prefer JOURNeY_A | -0.05 | 125 | -0.86 | 74 | -1.07 | 59 |
|  | 0.85 |  | 1.06 |  | 1.09 |  |
| Prefer JOURNeY_NonA | 0.02 | 327 | -0.78 | 166 | -1.00 | 133 |
|  | 0.91 |  | 1.02 |  | 1.03 |  |
| Secondary Outcomes | | | | | | |
|  | Pre-Treatment | | Post-Treatment | | Follow-Up | |
|  | M  SD | n | M  SD | n | M  SD | n |
|  | PHQ-9 | | | | | |
| Prefer JOURNeY_A | 8.65 | 125 | 7.07 | 74 | 7.69 | 59 |
|  | 4.99 |  | 4.22 |  | 5.72 |  |
| Prefer JOURNeY_NonA | 9.29 | 327 | 7.88 | 166 | 7.70 | 132 |
|  | 4.70 |  | 4.92 |  | 5.45 |  |
|  | SF12 physical | | | | | |
| Prefer JOURNeY_A | 53.11 | 125 | 51.44 | 74 | 53.16 | 59 |
|  | 6.56 |  | 7.25 |  | 6.00 |  |
| Prefer JOURNeY_NonA | 52.90 | 327 | 51.43 | 165 | 51.69 | 132 |
|  | 6.77 |  | 7.65 |  | 7.80 |  |
|  | SF12 mental | | | | | |
| Prefer JOURNeY_A | 37.34 | 125 | 42.40 | 74 | 41.44 | 59 |
|  | 9.50 |  | 10.41 |  | 12.18 |  |
| Prefer JOURNeY_NonA | 36.18 | 327 | 40.12 | 165 | 39.73 | 132 |
|  | 9.68 |  | 10.61 |  | 11.43 |  |
|  | ISMI NoRes | | | | | |
| Prefer JOURNeY_A | 1.85 | 125 | 1.78 | 74 | 1.76 | 58 |
|  | 0.48 |  | 0.56 |  | 0.55 |  |
| Prefer JOURNeY_NonA | 1.96 | 327 | 1.76 | 165 | 1.76 | 130 |
|  | 0.49 |  | 0.50 |  | 0.47 |  |
|  | IASMHS | | | | | |
| Prefer JOURNeY_A | 66.12 | 125 | 66.54 | 74 | 68.11 | 57 |
|  | 12.61 |  | 13.77 |  | 14.58 |  |
| Prefer JOURNeY_NonA | 65.76 | 327 | 69.42 | 165 | 70.17 | 130 |
|  | 11.91 |  | 11.83 |  | 12.20 |  |
|  | LPFS total | | | | | |
| Prefer JOURNeY_A | 27.85 | 125 | 26.68 | 73 | 26.26 | 57 |
|  | 5.95 |  | 6.25 |  | 6.45 |  |
| Prefer JOURNeY_NonA | 29.01 | 327 | 27.65 | 164 | 27.23 | 130 |
|  | 5.51 |  | 6.04 |  | 6.04 |  |
|  | LPFS self | | | | | |
| Prefer JOURNeY_A | 15.34 | 125 | 14.25 | 73 | 14.07 | 57 |
|  | 3.59 |  | 3.74 |  | 3.95 |  |
| Prefer JOURNeY_NonA | 16.24 | 327 | 15.27 | 164 | 15.04 | 130 |
|  | 3.27 |  | 3.66 |  | 3.68 |  |
|  | LPFS interpersonal | | | | | |
| Prefer JOURNeY_A | 12.50 | 125 | 12.44 | 73 | 12.19 | 57 |
|  | 3.38 |  | 3.34 |  | 3.31 |  |
| Prefer JOURNeY_NonA | 12.78 | 327 | 12.37 | 164 | 12.19 | 130 |
|  | 3.21 |  | 3.39 |  | 3.36 |  |
|  | PCP Concerns | | | | | |
| Prefer JOURNeY_A | 44.22 | 125 |  |  |  |  |
|  | 12.98 |  |  |  |  |  |
| Prefer JOURNeY_NonA | 40.99 | 327 |  |  |  |  |
|  | 12.48 |  |  |  |  |  |
|  | PCP General Caution | | | | | |
| Prefer JOURNeY_A | 13.96 | 125 |  |  |  |  |
|  | 4.65 |  |  |  |  |  |
| Prefer JOURNeY_NonA | 13.26 | 327 |  |  |  |  |
|  | 4.42 |  |  |  |  |  |
|  | PCP Technical Protection | | | | | |
| Prefer JOURNeY_A | 19.59 | 125 |  |  |  |  |
|  | 4.86 |  |  |  |  |  |
| Prefer JOURNeY_NonA | 18.98 | 327 |  |  |  |  |
|  | 5.30 |  |  |  |  |  |

Appendix B.7

Observed means and standard deviations for preference strength for all timepoints for whole sample split by indicated baseline preference

| Preference Strength | | | | | | | | | |  |
| --- | --- | --- | --- | --- | --- | --- | --- | --- | --- | --- |
|  | Pre-  Treatment | | Mid1-  Treatment | | Mid2-  Treatment | | Post-  Treatment | | Follow-up  Treatment | |
|  | M  SD | n | M  SD | n | M  SD | n | M  SD | n | M  SD | n |
|  | Preference Strength | | | | | | | | | |
| Prefer JOURNeY_A | 2.39 | 125 | 2.66 | 92 | 2.66 | 76 | 2.60 | 73 | 2.49 | 57 |
|  | 0.82 |  | 0.88 |  | 0.83 |  | 0.86 |  | 0.93 |  |
| Prefer JOURNeY_NonA | 2.50 | 327 | 2.66 | 236 | 2.55 | 182 | 2.59 | 163 | 2.47 | 128 |
|  | 0.90 |  | 0.88 |  | 0.93 |  | 1.02 |  | 0.92 |  |

Appendix C.1

Omnibus Tests of Fixed Effects (Time, Program Version, and Time × Program Version) from Linear Mixed Models with Randomized Arms. Pre-Post Model

| Questionnaire | Effect | NumDF | DenDF | F value | p |
| --- | --- | --- | --- | --- | --- |
| SPS | timepoint | 1 | 143.76 | 88.64 | < .001 |
| SPS | rand_overall | 1 | 243.33 | 0.86 | 0.35 |
| SPS | timepoint:rand_overall | 1 | 143.76 | 0.12 | 0.73 |
| SIAS | timepoint | 1 | 154.16 | 96.93 | < .001 |
| SIAS | rand_overall | 1 | 249.23 | 1.15 | 0.29 |
| SIAS | timepoint:rand_overall | 1 | 154.16 | 0.04 | 0.84 |
| SA | timepoint | 1 | 153.54 | 106.94 | < .001 |
| SA | rand_overall | 1 | 248.95 | 1.18 | 0.28 |
| SA | timepoint:rand_overall | 1 | 153.54 | 0.09 | 0.76 |
| PHQ9 | timepoint | 1 | 137.66 | 5.93 | 0.02 |
| PHQ9 | rand_overall | 1 | 236.61 | 1.16 | 0.28 |
| PHQ9 | timepoint:rand_overall | 1 | 137.66 | < .001 | 0.98 |
| SF12_P | timepoint | 1 | 153.92 | 4.16 | 0.04 |
| SF12_P | rand_overall | 1 | 245.39 | 1.58 | 0.21 |
| SF12_P | timepoint:rand_overall | 1 | 153.92 | < .001 | 0.98 |
| SF12_M | timepoint | 1 | 153.34 | 28.33 | < .001 |
| SF12_M | rand_overall | 1 | 241.62 | 0.29 | 0.59 |
| SF12_M | timepoint:rand_overall | 1 | 153.34 | 0.01 | 0.93 |
| ISMI_NR | timepoint | 1 | 139.50 | 30.96 | < .001 |
| ISMI_NR | rand_overall | 1 | 245.99 | 0.03 | 0.85 |
| ISMI_NR | timepoint:rand_overall | 1 | 139.50 | 0.32 | 0.57 |
| IASMHS | timepoint | 1 | 138.05 | 13.70 | < .001 |
| IASMHS | rand_overall | 1 | 244.72 | 0.12 | 0.73 |
| IASMHS | timepoint:rand_overall | 1 | 138.05 | 3.85 | 0.05 |
| LPFS | timepoint | 1 | 133.91 | 10.18 | 0.002 |
| LPFS | rand_overall | 1 | 242.77 | 1.40 | 0.24 |
| LPFS | timepoint:rand_overall | 1 | 133.91 | 0.12 | 0.73 |
| LPFS_S | timepoint | 1 | 134.22 | 14.04 | < .001 |
| LPFS_S | rand_overall | 1 | 243.26 | < .001 | 0.97 |
| LPFS_S | timepoint:rand_overall | 1 | 134.22 | 0.43 | 0.52 |
| LPFS_I | timepoint | 1 | 135.71 | 2.39 | 0.12 |
| LPFS_I | rand_overall | 1 | 243.45 | 4.21 | 0.04 |
| LPFS_I | timepoint:rand_overall | 1 | 135.71 | 0.01 | 0.93 |

Note. F-tests are based on Type III analysis of variance with Satterthwaite degrees of freedom. Time refers to the change from pre- to post-assessment. rand_overall = condition participants were assigned to

Appendix C.2

Omnibus Tests of Fixed Effects (Time, Program Version, and Time × Program Version) from Linear Mixed Models with Preference Arms. Pre-Post Model

| Questionnaire | Effect | NumDF | DenDF | F value | p |
| --- | --- | --- | --- | --- | --- |
| SPS | timepoint | 1 | 137.20 | 104.90 | < .001 |
| SPS | rand_overall | 1 | 229.31 | 1.70 | 0.19 |
| SPS | timepoint:rand_overall | 1 | 137.20 | 0.18 | 0.67 |
| SIAS | timepoint | 1 | 144.23 | 102.48 | < .001 |
| SIAS | rand_overall | 1 | 233.79 | 0.70 | 0.40 |
| SIAS | timepoint:rand_overall | 1 | 144.23 | 0.54 | 0.47 |
| SA | timepoint | 1 | 140.89 | 131.72 | < .001 |
| SA | rand_overall | 1 | 231.87 | 0.05 | 0.82 |
| SA | timepoint:rand_overall | 1 | 140.89 | 0.04 | 0.84 |
| PHQ9 | timepoint | 1 | 134.54 | 10.46 | .002 |
| PHQ9 | rand_overall | 1 | 222.54 | 0.82 | 0.37 |
| PHQ9 | timepoint:rand_overall | 1 | 134.54 | 0.46 | 0.50 |
| SF12_P | timepoint | 1 | 140.29 | 8.41 | .004 |
| SF12_P | rand_overall | 1 | 229.02 | 1.15 | 0.29 |
| SF12_P | timepoint:rand_overall | 1 | 140.29 | 0.09 | 0.76 |
| SF12_M | timepoint | 1 | 141.67 | 17.11 | < .001 |
| SF12_M | rand_overall | 1 | 230.82 | 1.77 | 0.19 |
| SF12_M | timepoint:rand_overall | 1 | 141.67 | 0.25 | 0.62 |
| ISMI_NR | timepoint | 1 | 127.81 | 20.43 | < .001 |
| ISMI_NR | rand_overall | 1 | 228.48 | 0.62 | 0.43 |
| ISMI_NR | timepoint:rand_overall | 1 | 127.81 | 0.01 | 0.92 |
| IASMHS | timepoint | 1 | 126.31 | 7.81 | 0.01 |
| IASMHS | rand_overall | 1 | 227.50 | 1.02 | 0.31 |
| IASMHS | timepoint:rand_overall | 1 | 126.31 | 1.56 | 0.21 |
| LPFS | timepoint | 1 | 129.52 | 3.92 | 0.05 |
| LPFS | rand_overall | 1 | 231.85 | 0.95 | 0.33 |
| LPFS | timepoint:rand_overall | 1 | 129.52 | 0.45 | 0.50 |
| LPFS_S | timepoint | 1 | 135.18 | 8.16 | .005 |
| LPFS_S | rand_overall | 1 | 234.53 | 5.76 | 0.02 |
| LPFS_S | timepoint:rand_overall | 1 | 135.18 | 0.86 | 0.36 |
| LPFS_I | timepoint | 1 | 133.83 | 0.07 | 0.79 |
| LPFS_I | rand_overall | 1 | 233.39 | 0.61 | 0.44 |
| LPFS_I | timepoint:rand_overall | 1 | 133.83 | 0.06 | 0.81 |

Note. F-tests are based on Type III analysis of variance with Satterthwaite degrees of freedom. Time refers to the change from pre- to post-assessment. rand_overall = condition participants were assigned to

Appendix C.3

Omnibus Tests of Fixed Effects (Time, Program Version, and Time × Program Version) from Linear Mixed Models with Preference matched vs. not matched conditions. Pre-Post Model

| Questionnaire | Effect | NumDF | DenDF | F value | p |
| --- | --- | --- | --- | --- | --- |
| SPS | timepoint | 1 | 280.96 | 168.46 | < .001 |
| SPS | preference_match | 1 | 472.65 | 0.11 | 0.74 |
| SPS | timepoint:preference_match | 1 | 280.96 | 0.67 | 0.41 |
| SIAS | timepoint | 1 | 299.44 | 175.50 | < .001 |
| SIAS | preference_match | 1 | 483.44 | 0.43 | 0.51 |
| SIAS | timepoint:preference_match | 1 | 299.44 | 0.27 | 0.60 |
| SA | timepoint | 1 | 294.99 | 206.87 | < .001 |
| SA | preference_match | 1 | 481.14 | 0.03 | 0.85 |
| SA | timepoint:preference_match | 1 | 294.99 | 0.51 | 0.48 |
| PHQ9 | timepoint | 1 | 272.58 | 14.92 | < .001 |
| PHQ9 | preference_match | 1 | 459.24 | 0.51 | 0.47 |
| PHQ9 | timepoint:preference_match | 1 | 272.58 | 0.54 | 0.46 |
| SF12_P | timepoint | 1 | 295.42 | 7.36 | 0.01 |
| SF12_P | preference_match | 1 | 476.46 | 2.20 | 0.14 |
| SF12_P | timepoint:preference_match | 1 | 295.42 | 1.13 | 0.29 |
| SF12_M | timepoint | 1 | 292.94 | 49.96 | < .001 |
| SF12_M | preference_match | 1 | 475.05 | 0.01 | 0.90 |
| SF12_M | timepoint:preference_match | 1 | 292.94 | 4.49 | 0.04 |
| ISMI_NR | timepoint | 1 | 266.14 | 60.21 | < .001 |
| ISMI_NR | preference_match | 1 | 474.34 | 0.28 | 0.60 |
| ISMI_NR | timepoint:preference_match | 1 | 266.14 | 5.47 | 0.02 |
| IASMHS | timepoint | 1 | 266.71 | 20.76 | < .001 |
| IASMHS | preference_match | 1 | 474.29 | 3.41 | 0.07 |
| IASMHS | timepoint:preference_match | 1 | 266.71 | 0.24 | 0.62 |
| LPFS | timepoint | 1 | 264.17 | 17.52 | < .001 |
| LPFS | preference_match | 1 | 474.46 | 0.01 | 0.93 |
| LPFS | timepoint:preference_match | 1 | 264.17 | 1.57 | 0.21 |
| LPFS_S | timepoint | 1 | 269.97 | 29.37 | < .001 |
| LPFS_S | preference_match | 1 | 477.50 | 0.29 | 0.59 |
| LPFS_S | timepoint:preference_match | 1 | 269.97 | 3.09 | 0.08 |
| LPFS_I | timepoint | 1 | 271.39 | 1.86 | 0.17 |
| LPFS_I | preference_match | 1 | 477.58 | 0.17 | 0.68 |
| LPFS_I | timepoint:preference_match | 1 | 271.39 | 0.06 | 0.81 |

Note. F-tests are based on Type III analysis of variance with Satterthwaite degrees of freedom. Time refers to the change from pre- to post-assessment. Preference_match refers to whether or not indicated preference at baseline was matched in condition assignment. rand_overall = condition participants were assigned to

Appendix C.4

Omnibus Tests of Fixed Effects (Time, Program Version, and Time × Program Version) from Linear Mixed Models with Preference matched vs. not matched detailed conditions. Pre-Post Model

| Questionnaire | Effect | NumDF | DenDF | F value | p |
| --- | --- | --- | --- | --- | --- |
| SPS | timepoint | 1 | 279.89 | 100.82 | < .001 |
| SPS | preference_notmatched | 2 | 470.98 | 0.06 | 0.95 |
| SPS | timepoint:preference_notmatched | 2 | 279.83 | 0.51 | 0.60 |
| SIAS | timepoint | 1 | 298.25 | 102.16 | < .001 |
| SIAS | preference_notmatched | 2 | 481.56 | 0.22 | 0.80 |
| SIAS | timepoint:preference_notmatched | 2 | 298.19 | 0.25 | 0.78 |
| SA | timepoint | 1 | 293.89 | 121.89 | < .001 |
| SA | preference_notmatched | 2 | 479.38 | 0.02 | 0.98 |
| SA | timepoint:preference_notmatched | 2 | 293.83 | 0.42 | 0.66 |
| PHQ9 | timepoint | 1 | 271.24 | 9.81 | < .001 |
| PHQ9 | preference_notmatched | 2 | 457.05 | 0.60 | 0.55 |
| PHQ9 | timepoint:preference_notmatched | 2 | 271.18 | 0.29 | 0.75 |
| SF12_P | timepoint | 1 | 293.74 | 2.64 | 0.11 |
| SF12_P | preference_notmatched | 2 | 473.84 | 1.15 | 0.32 |
| SF12_P | timepoint:preference_notmatched | 2 | 293.93 | 0.60 | 0.55 |
| SF12_M | timepoint | 1 | 291.35 | 35.53 | < .001 |
| SF12_M | preference_notmatched | 2 | 472.56 | 0.14 | 0.87 |
| SF12_M | timepoint:preference_notmatched | 2 | 291.53 | 2.35 | 0.10 |
| ISMI_NR | timepoint | 1 | 265.14 | 36.84 | < .001 |
| ISMI_NR | preference_notmatched | 2 | 472.53 | 0.73 | 0.48 |
| ISMI_NR | timepoint:preference_notmatched | 2 | 265.24 | 2.86 | 0.06 |
| IASMHS | timepoint | 1 | 265.14 | 16.65 | < .001 |
| IASMHS | preference_notmatched | 2 | 472.14 | 1.72 | 0.18 |
| IASMHS | timepoint:preference_notmatched | 2 | 265.24 | 0.92 | 0.40 |
| LPFS | timepoint | 1 | 262.77 | 13.41 | < .001 |
| LPFS | preference_notmatched | 2 | 472.18 | 0.16 | 0.85 |
| LPFS | timepoint:preference_notmatched | 2 | 262.89 | 0.94 | 0.39 |
| LPFS_S | timepoint | 1 | 268.49 | 23.32 | < .001 |
| LPFS_S | preference_notmatched | 2 | 475.15 | 0.45 | 0.64 |
| LPFS_S | timepoint:preference_notmatched | 2 | 268.62 | 1.89 | 0.15 |
| LPFS_I | timepoint | 1 | 269.92 | 1.22 | 0.27 |
| LPFS_I | preference_notmatched | 2 | 475.16 | 0.10 | 0.90 |
| LPFS_I | timepoint:preference_notmatched | 2 | 270.06 | 0.03 | 0.97 |

F-tests are based on Type III analysis of variance with Satterthwaite degrees of freedom. Time refers to the change from pre- to post-assessment. Matched vs. not matched detailed refers to preference matched vs. preference for non-anonymous not matched vs. preference for anonymous not matched.

Appendix D.1

Omnibus Tests of Fixed Effects (Time, Program Version, and Time × Program Version) from Linear Mixed Models with Randomized Arms. Pre-Post-Followup Model

| Questionnaire | Effect | NumDF | DenDF | F value | p |
| --- | --- | --- | --- | --- | --- |
| SPS | timepoint | 2 | 241.93 | 89.15 | < .001 |
| SPS | rand_overall | 1 | 245.35 | 2.01 | 0.16 |
| SPS | timepoint:rand_overall | 2 | 241.93 | 0.51 | 0.60 |
| SIAS | timepoint | 2 | 252.38 | 87.18 | < .001 |
| SIAS | rand_overall | 1 | 253.09 | 2.10 | 0.15 |
| SIAS | timepoint:rand_overall | 2 | 252.38 | 0.25 | 0.78 |
| SA | timepoint | 2 | 250.28 | 103.46 | < .001 |
| SA | rand_overall | 1 | 252.27 | 2.41 | 0.12 |
| SA | timepoint:rand_overall | 2 | 250.28 | 0.41 | 0.66 |
| PHQ9 | timepoint | 2 | 245.46 | 3.21 | 0.04 |
| PHQ9 | rand_overall | 1 | 239.70 | 3.10 | 0.08 |
| PHQ9 | timepoint:rand_overall | 2 | 245.46 | 0.91 | 0.40 |
| SF12_P | timepoint | 2 | 268.44 | 1.82 | 0.16 |
| SF12_P | rand_overall | 1 | 245.21 | 0.40 | 0.53 |
| SF12_P | timepoint:rand_overall | 2 | 268.44 | 1.04 | 0.35 |
| SF12_M | timepoint | 2 | 265.26 | 13.91 | < .001 |
| SF12_M | rand_overall | 1 | 246.09 | 1.05 | 0.31 |
| SF12_M | timepoint:rand_overall | 2 | 265.26 | 0.34 | 0.71 |
| ISMI_NR | timepoint | 2 | 239.36 | 19.04 | < .001 |
| ISMI_NR | rand_overall | 1 | 248.39 | 0.44 | 0.51 |
| ISMI_NR | timepoint:rand_overall | 2 | 239.36 | 0.97 | 0.38 |
| IASMHS | timepoint | 2 | 237.51 | 8.20 | < .001 |
| IASMHS | rand_overall | 1 | 248.82 | 0.21 | 0.64 |
| IASMHS | timepoint:rand_overall | 2 | 237.51 | 3.35 | 0.04 |
| LPFS | timepoint | 2 | 234.53 | 8.54 | < .001 |
| LPFS | rand_overall | 1 | 246.07 | 3.67 | 0.06 |
| LPFS | timepoint:rand_overall | 2 | 234.53 | 1.99 | 0.14 |
| LPFS_S | timepoint | 2 | 235.38 | 13.16 | < .001 |
| LPFS_S | rand_overall | 1 | 247.38 | 0.68 | 0.41 |
| LPFS_S | timepoint:rand_overall | 2 | 235.38 | 2.94 | 0.05 |
| LPFS_I | timepoint | 2 | 236.53 | 1.52 | 0.22 |
| LPFS_I | rand_overall | 1 | 246.40 | 6.41 | 0.01 |
| LPFS_I | timepoint:rand_overall | 2 | 236.53 | 0.39 | 0.68 |

Note: F-tests are based on Type III analysis of variance with Satterthwaite degrees of freedom. Time refers to the change from pre to post to followup-assessment. rand_overall = condition participants were assigned to

Appendix D.2

Omnibus Tests of Fixed Effects (Time, Program Version, and Time × Program Version) from Linear Mixed Models with Preference Arms. Pre-Post-Followup Model

| Questionnaire | Effect | NumDF | DenDF | F value | p |
| --- | --- | --- | --- | --- | --- |
| SPS | timepoint | 2 | 231.75 | 79.38 | < .001 |
| SPS | rand_overall | 1 | 234.59 | 1.42 | 0.24 |
| SPS | timepoint:rand_overall | 2 | 231.75 | 0.12 | 0.89 |
| SIAS | timepoint | 2 | 235.81 | 89.08 | < .001 |
| SIAS | rand_overall | 1 | 243.07 | 1.03 | 0.31 |
| SIAS | timepoint:rand_overall | 2 | 235.81 | 0.43 | 0.65 |
| SA | timepoint | 2 | 235.22 | 105.62 | < .001 |
| SA | rand_overall | 1 | 240.56 | 0.003 | 0.96 |
| SA | timepoint:rand_overall | 2 | 235.22 | 0.13 | 0.87 |
| PHQ9 | timepoint | 2 | 229.54 | 5.42 | 0.01 |
| PHQ9 | rand_overall | 1 | 236.54 | 0.20 | 0.66 |
| PHQ9 | timepoint:rand_overall | 2 | 229.54 | 1.03 | 0.36 |
| SF12_P | timepoint | 2 | 236.22 | 4.36 | 0.01 |
| SF12_P | rand_overall | 1 | 237.67 | 1.80 | 0.18 |
| SF12_P | timepoint:rand_overall | 2 | 236.22 | 0.28 | 0.76 |
| SF12_M | timepoint | 2 | 244.38 | 8.88 | < .001 |
| SF12_M | rand_overall | 1 | 240.19 | 0.92 | 0.34 |
| SF12_M | timepoint:rand_overall | 2 | 244.38 | 0.56 | 0.57 |
| ISMI_NR | timepoint | 2 | 216.66 | 17.84 | < .001 |
| ISMI_NR | rand_overall | 1 | 236.09 | 0.85 | 0.36 |
| ISMI_NR | timepoint:rand_overall | 2 | 216.66 | 0.11 | 0.89 |
| IASMHS | timepoint | 2 | 214.06 | 11.36 | < .001 |
| IASMHS | rand_overall | 1 | 234.76 | 1.10 | 0.30 |
| IASMHS | timepoint:rand_overall | 2 | 214.06 | 0.85 | 0.43 |
| LPFS | timepoint | 2 | 221.70 | 3.37 | 0.04 |
| LPFS | rand_overall | 1 | 241.29 | 0.80 | 0.37 |
| LPFS | timepoint:rand_overall | 2 | 221.70 | 0.21 | 0.81 |
| LPFS_S | timepoint | 2 | 228.82 | 4.89 | 0.01 |
| LPFS_S | rand_overall | 1 | 245.10 | 4.26 | 0.04 |
| LPFS_S | timepoint:rand_overall | 2 | 228.82 | 0.95 | 0.39 |
| LPFS_I | timepoint | 2 | 225.46 | 1.16 | 0.32 |
| LPFS_I | rand_overall | 1 | 242.13 | 0.30 | 0.58 |
| LPFS_I | timepoint:rand_overall | 2 | 225.46 | 0.19 | 0.82 |

Note: F-tests are based on Type III analysis of variance with Satterthwaite degrees of freedom. Time refers to the change from pre to post to followup-assessment. rand_overall = condition participants were assigned to

Appendix D.3

Omnibus Tests of Fixed Effects (Time, Program Version, and Time × Program Version) from Linear Mixed Models with Preference matched vs. not matched conditions. Pre-Post-Followup Model

| Questionnaire | Effect | NumDF | DenDF | F value | p |
| --- | --- | --- | --- | --- | --- |
| SPS | timepoint | 2 | 475.01 | 153.13 | < .001 |
| SPS | preference_match | 1 | 473.90 | 0.005 | 0.94 |
| SPS | timepoint:preference_match | 2 | 475.01 | 0.68 | 0.51 |
| SIAS | timepoint | 2 | 491.05 | 161.41 | < .001 |
| SIAS | preference_match | 1 | 488.92 | 0.90 | 0.34 |
| SIAS | timepoint:preference_match | 2 | 491.05 | 0.49 | 0.61 |
| SA | timepoint | 2 | 487.97 | 190.97 | < .001 |
| SA | preference_match | 1 | 486.59 | 0.25 | 0.62 |
| SA | timepoint:preference_match | 2 | 487.97 | 0.70 | 0.50 |
| PHQ9 | timepoint | 2 | 476.38 | 9.91 | < .001 |
| PHQ9 | preference_match | 1 | 467.99 | 2.34 | 0.13 |
| PHQ9 | timepoint:preference_match | 2 | 476.38 | 2.40 | 0.09 |
| SF12_P | timepoint | 2 | 506.91 | 3.44 | 0.03 |
| SF12_P | preference_match | 1 | 475.55 | 3.18 | 0.08 |
| SF12_P | timepoint:preference_match | 2 | 506.91 | 0.90 | 0.41 |
| SF12_M | timepoint | 2 | 507.88 | 27.39 | < .001 |
| SF12_M | preference_match | 1 | 479.59 | 0.41 | 0.52 |
| SF12_M | timepoint:preference_match | 2 | 507.88 | 4.04 | 0.02 |
| ISMI_NR | timepoint | 2 | 457.21 | 40.43 | < .001 |
| ISMI_NR | preference_match | 1 | 478.04 | 0.07 | 0.78 |
| ISMI_NR | timepoint:preference_match | 2 | 457.21 | 3.28 | 0.04 |
| IASMHS | timepoint | 2 | 455.89 | 18.59 | < .001 |
| IASMHS | preference_match | 1 | 481.02 | 2.36 | 0.13 |
| IASMHS | timepoint:preference_match | 2 | 455.89 | 0.52 | 0.60 |
| LPFS | timepoint | 2 | 458.59 | 15.20 | < .001 |
| LPFS | preference_match | 1 | 482.49 | 0.18 | 0.67 |
| LPFS | timepoint:preference_match | 2 | 458.59 | 1.98 | 0.14 |
| LPFS_S | timepoint | 2 | 466.75 | 24.52 | < .001 |
| LPFS_S | preference_match | 1 | 487.36 | 0.03 | 0.87 |
| LPFS_S | timepoint:preference_match | 2 | 466.75 | 3.84 | 0.02 |
| LPFS_I | timepoint | 2 | 464.84 | 2.13 | 0.12 |
| LPFS_I | preference_match | 1 | 483.81 | 0.35 | 0.55 |
| LPFS_I | timepoint:preference_match | 2 | 464.84 | 0.13 | 0.88 |

Note: F-tests are based on Type III analysis of variance with Satterthwaite degrees of freedom. Time refers to the change from pre to post to followup-assessment. Preference_match refers to whether or not indicated preference at baseline was matched in condition assignment.

Appendix D.4

Omnibus Tests of Fixed Effects (Time, Program Version, and Time × Program Version) from Linear Mixed Models with Preference matched vs. not matched detailed conditions. Pre-Post-Followup Model

| Questionnaire | Effect | NumDF | DenDF | F value | p |
| --- | --- | --- | --- | --- | --- |
| SPS | timepoint | 2 | 470.27 | 92.50 | < .001 |
| SPS | preference_notmatched | 2 | 464.91 | 0.02 | 0.98 |
| SPS | timepoint:preference_notmatched | 4 | 471.65 | 0.43 | 0.79 |
| SIAS | timepoint | 2 | 486.31 | 98.05 | < .001 |
| SIAS | preference_notmatched | 2 | 479.95 | 0.44 | 0.64 |
| SIAS | timepoint:preference_notmatched | 4 | 487.70 | 0.28 | 0.89 |
| SA | timepoint | 2 | 483.31 | 115.68 | < .001 |
| SA | preference_notmatched | 2 | 477.70 | 0.13 | 0.88 |
| SA | timepoint:preference_notmatched | 4 | 484.70 | 0.38 | 0.82 |
| PHQ9 | timepoint | 2 | 471.00 | 6.99 | 0.001 |
| PHQ9 | preference_notmatched | 2 | 458.07 | 1.34 | 0.26 |
| PHQ9 | timepoint:preference_notmatched | 4 | 472.70 | 1.28 | 0.28 |
| SF12_P | timepoint | 2 | 500.70 | 1.39 | 0.25 |
| SF12_P | preference_notmatched | 2 | 464.53 | 1.60 | 0.20 |
| SF12_P | timepoint:preference_notmatched | 4 | 502.96 | 0.46 | 0.76 |
| SF12_M | timepoint | 2 | 501.71 | 20.62 | < .001 |
| SF12_M | preference_notmatched | 2 | 468.50 | 0.34 | 0.71 |
| SF12_M | timepoint:preference_notmatched | 4 | 503.93 | 2.05 | 0.09 |
| ISMI_NR | timepoint | 2 | 453.24 | 24.50 | < .001 |
| ISMI_NR | preference_notmatched | 2 | 469.94 | 0.33 | 0.72 |
| ISMI_NR | timepoint:preference_notmatched | 4 | 454.39 | 1.98 | 0.10 |
| IASMHS | timepoint | 2 | 452.04 | 12.88 | < .001 |
| IASMHS | preference_notmatched | 2 | 474.62 | 1.16 | 0.32 |
| IASMHS | timepoint:preference_notmatched | 4 | 452.88 | 1.05 | 0.38 |
| LPFS | timepoint | 2 | 454.33 | 9.74 | < .001 |
| LPFS | preference_notmatched | 2 | 475.54 | 0.10 | 0.90 |
| LPFS | timepoint:preference_notmatched | 4 | 455.33 | 1.92 | 0.11 |
| LPFS_S | timepoint | 2 | 462.46 | 17.73 | < .001 |
| LPFS_S | preference_notmatched | 2 | 480.11 | 0.09 | 0.92 |
| LPFS_S | timepoint:preference_notmatched | 4 | 463.60 | 2.55 | 0.04 |
| LPFS_I | timepoint | 2 | 460.29 | 0.83 | 0.43 |
| LPFS_I | preference_notmatched | 2 | 476.33 | 0.26 | 0.77 |
| LPFS_I | timepoint:preference_notmatched | 4 | 461.50 | 0.75 | 0.56 |

F-tests are based on Type III analysis of variance with Satterthwaite degrees of freedom. Time refers to the change from pre to post to followup-assessment. Matched vs. not matched detailed refers to preference matched vs. preference for non-anonymous not matched vs. preference for anonymous not matched.

Appendix E.1

Effect sizes Cohen’s d for between-group effect sizes in the randomized arms for pre-post

| outcome | timepoint | emmean_g1 | emmean_g3 | SD_g1 | SD_g3 | n_g1 | n_g3 | sd_pooled | d_lower | d_upper | d |
| --- | --- | --- | --- | --- | --- | --- | --- | --- | --- | --- | --- |
| SPS | pre | 36.74 | 38.71 | 13.41 | 13.41 | 113 | 114 | 13.41 | -0.11 | 0.41 | 0.15 |
| SPS | post | 27.52 | 28.78 | 16.16 | 17.38 | 113 | 114 | 16.79 | -0.19 | 0.34 | 0.07 |
| SIAS | pre | 48.32 | 50.26 | 12.60 | 12.60 | 113 | 114 | 12.60 | -0.11 | 0.41 | 0.15 |
| SIAS | post | 38.72 | 40.26 | 15.35 | 16.57 | 113 | 114 | 15.98 | -0.16 | 0.36 | 0.10 |
| SA composite | pre | -0.02 | 0.14 | 0.98 | 0.98 | 113 | 114 | 0.98 | -0.10 | 0.42 | 0.16 |
| SA composite | post | -0.79 | -0.68 | 1.19 | 1.29 | 113 | 114 | 1.24 | -0.17 | 0.35 | 0.09 |
| PHQ-9 | pre | 9.19 | 9.89 | 5.01 | 5.01 | 113 | 114 | 5.01 | -0.12 | 0.40 | 0.14 |
| PHQ-9 | post | 8.24 | 8.95 | 6.07 | 6.54 | 113 | 114 | 6.31 | -0.15 | 0.37 | 0.11 |
| SF-12 Physical | pre | 51.78 | 52.93 | 7.04 | 7.04 | 113 | 114 | 7.04 | -0.10 | 0.42 | 0.16 |
| SF-12 Physical | post | 50.59 | 51.71 | 8.76 | 9.44 | 113 | 114 | 9.11 | -0.14 | 0.38 | 0.12 |
| SF-12 Mental | pre | 35.26 | 34.64 | 9.95 | 9.95 | 113 | 114 | 9.95 | -0.32 | 0.20 | -0.06 |
| SF-12 Mental | post | 39.90 | 39.14 | 12.50 | 13.49 | 113 | 114 | 13.00 | -0.32 | 0.20 | -0.06 |
| ISMI Total (No Res) | pre | 1.98 | 1.97 | 0.49 | 0.49 | 113 | 114 | 0.49 | -0.27 | 0.25 | -0.01 |
| ISMI Total (No Res) | post | 1.78 | 1.81 | 0.58 | 0.61 | 113 | 114 | 0.60 | -0.21 | 0.31 | 0.05 |
| IASMHS Total | pre | 65.32 | 64.25 | 12.26 | 12.26 | 113 | 114 | 12.26 | -0.35 | 0.17 | -0.09 |
| IASMHS Total | post | 66.76 | 68.95 | 14.40 | 15.27 | 113 | 114 | 14.85 | -0.11 | 0.41 | 0.15 |
| LPFS Total | pre | 28.71 | 29.46 | 5.70 | 5.70 | 113 | 114 | 5.70 | -0.13 | 0.39 | 0.13 |
| LPFS Total | post | 27.35 | 28.36 | 6.69 | 7.13 | 113 | 114 | 6.91 | -0.11 | 0.41 | 0.15 |
| LPFS Self | pre | 16.23 | 16.10 | 3.52 | 3.52 | 113 | 114 | 3.52 | -0.30 | 0.22 | -0.04 |
| LPFS Self | post | 15.19 | 15.37 | 4.12 | 4.39 | 113 | 114 | 4.26 | -0.22 | 0.30 | 0.04 |
| LPFS Interpersonal | pre | 12.48 | 13.36 | 3.21 | 3.21 | 113 | 114 | 3.21 | 0.01 | 0.54 | 0.27 |
| LPFS Interpersonal | post | 12.15 | 13.00 | 3.79 | 4.05 | 113 | 114 | 3.93 | -0.05 | 0.48 | 0.21 |

Note: emmean_g1 = estimated mean group 1; emmean_g3 = estimated mean group 3; group 1 = random anon; group 3 = random non-anon; SD = standard deviation calculated with standard error and n; sd_pooled = pooled standard deviation; d_lower = lower end of 95% confidence interval; d_upper = upper end of 95% confidence_interval; d = Cohen’s d

Standard deviations were derived from model-based standard errors (SD = SE × √n) rather than observed sample standard deviations, as the estimated marginal means from the mixed-effects models do not have directly corresponding observed SDs. Cohen's d was computed from these model-derived pooled SDs.

Appendix E.2

Effect sizes Cohen’s d for between-group effect sizes in the preference arms for pre-post

| outcome | timepoint | emmean_g2 | emmean_g4 | SD_g2 | SD_g4 | n_g2 | n_g4 | sd_pooled | d_lower | d_upper | d |
| --- | --- | --- | --- | --- | --- | --- | --- | --- | --- | --- | --- |
| SPS | pre | 34.03 | 35.96 | 12.55 | 12.55 | 64 | 161 | 12.55 | -0.14 | 0.44 | 0.15 |
| SPS | post | 23.70 | 26.45 | 14.66 | 15.61 | 64 | 161 | 15.35 | -0.11 | 0.47 | 0.18 |
| SIAS | pre | 48.94 | 48.22 | 11.74 | 11.74 | 64 | 161 | 11.74 | -0.35 | 0.23 | -0.06 |
| SIAS | post | 40.15 | 38.06 | 13.82 | 14.75 | 64 | 161 | 14.49 | -0.43 | 0.15 | -0.14 |
| SA composite | pre | -0.10 | -0.05 | 0.89 | 0.89 | 64 | 161 | 0.89 | -0.24 | 0.34 | 0.05 |
| SA composite | post | -0.88 | -0.86 | 1.04 | 1.11 | 64 | 161 | 1.09 | -0.28 | 0.30 | 0.01 |
| PHQ-9 | pre | 8.44 | 8.77 | 4.59 | 4.59 | 64 | 161 | 4.59 | -0.22 | 0.36 | 0.07 |
| PHQ-9 | post | 6.96 | 7.80 | 5.44 | 5.83 | 64 | 161 | 5.72 | -0.14 | 0.44 | 0.15 |
| SF-12 Physical | pre | 54.20 | 53.32 | 6.92 | 6.92 | 64 | 161 | 6.92 | -0.42 | 0.16 | -0.13 |
| SF-12 Physical | post | 52.74 | 51.52 | 8.18 | 8.75 | 64 | 161 | 8.59 | -0.43 | 0.15 | -0.14 |
| SF-12 Mental | pre | 39.11 | 37.66 | 9.80 | 9.80 | 64 | 161 | 9.80 | -0.44 | 0.14 | -0.15 |
| SF-12 Mental | post | 42.78 | 40.53 | 11.57 | 12.36 | 64 | 161 | 12.14 | -0.48 | 0.11 | -0.19 |
| ISMI Total (No Res) | pre | 1.84 | 1.90 | 0.51 | 0.51 | 64 | 161 | 0.51 | -0.17 | 0.41 | 0.12 |
| ISMI Total (No Res) | post | 1.70 | 1.76 | 0.56 | 0.59 | 64 | 161 | 0.58 | -0.20 | 0.38 | 0.09 |
| IASMHS Total | pre | 66.30 | 67.20 | 12.19 | 12.19 | 64 | 161 | 12.19 | -0.22 | 0.36 | 0.07 |
| IASMHS Total | post | 67.40 | 70.07 | 13.46 | 14.05 | 64 | 161 | 13.89 | -0.10 | 0.48 | 0.19 |
| LPFS Total | pre | 27.53 | 28.61 | 5.90 | 5.90 | 64 | 161 | 5.90 | -0.11 | 0.47 | 0.18 |
| LPFS Total | post | 27.07 | 27.67 | 6.58 | 6.84 | 64 | 161 | 6.77 | -0.20 | 0.38 | 0.09 |
| LPFS Self | pre | 14.81 | 16.21 | 3.41 | 3.41 | 64 | 161 | 3.41 | 0.12 | 0.70 | 0.41 |
| LPFS Self | post | 14.36 | 15.33 | 3.90 | 4.08 | 64 | 161 | 4.03 | -0.05 | 0.53 | 0.24 |
| LPFS Interpersonal | pre | 12.72 | 12.40 | 3.33 | 3.33 | 64 | 161 | 3.33 | -0.39 | 0.19 | -0.10 |
| LPFS Interpersonal | post | 12.71 | 12.28 | 3.80 | 3.97 | 64 | 161 | 3.93 | -0.40 | 0.18 | -0.11 |

Note: emmean_g2 = estimated mean group 2; emmean_g4 = estimated mean group 4; group 2 = prefer anon; group 4 = prefer non-anon; SD = standard deviation calculated with standard error and n; sd_pooled = pooled standard deviation; d_lower = lower end of 95% confidence interval; d_upper = upper end of 95% confidence_interval; d = Cohen’s d

Standard deviations were derived from model-based standard errors (SD = SE × √n) rather than observed sample standard deviations, as the estimated marginal means from the mixed-effects models do not have directly corresponding observed SDs. Cohen's d was computed from these model-derived pooled SDs.

Appendix E.3

Effect sizes Cohen’s d for between-group effect sizes in the preference matched vs. not matched conditions for pre-post

| outcome | timepoint | emmean_g0 | emmean_g1 | SD_g0 | SD_g1 | n_g0 | n_g1 | sd_pooled | d_lower | d_upper | d |
| --- | --- | --- | --- | --- | --- | --- | --- | --- | --- | --- | --- |
| SPS | pre | 36.31 | 37.40 | 13.05 | 13.05 | 342 | 110 | 13.05 | -0.13 | 0.30 | 0.08 |
| SPS | post | 26.92 | 26.75 | 16.16 | 15.70 | 342 | 110 | 16.05 | -0.23 | 0.20 | -0.01 |
| SIAS | pre | 48.97 | 48.52 | 12.18 | 12.18 | 342 | 110 | 12.18 | -0.25 | 0.18 | -0.04 |
| SIAS | post | 39.40 | 38.16 | 15.28 | 14.83 | 342 | 110 | 15.17 | -0.30 | 0.13 | -0.08 |
| SA composite | pre | -0.01 | 0.02 | 0.94 | 0.94 | 342 | 110 | 0.94 | -0.19 | 0.24 | 0.02 |
| SA composite | post | -0.79 | -0.85 | 1.17 | 1.14 | 342 | 110 | 1.16 | -0.27 | 0.16 | -0.05 |
| PHQ-9 | pre | 9.15 | 9.00 | 4.82 | 4.82 | 342 | 110 | 4.82 | -0.25 | 0.18 | -0.03 |
| PHQ-9 | post | 8.21 | 7.63 | 6.05 | 5.87 | 342 | 110 | 6.01 | -0.31 | 0.12 | -0.10 |
| SF-12 Physical | pre | 53.34 | 51.77 | 6.97 | 6.97 | 342 | 110 | 6.97 | -0.44 | -0.01 | -0.23 |
| SF-12 Physical | post | 51.64 | 51.03 | 8.86 | 8.64 | 342 | 110 | 8.80 | -0.28 | 0.15 | -0.07 |
| SF-12 Mental | pre | 36.87 | 35.38 | 9.97 | 9.97 | 342 | 110 | 9.97 | -0.36 | 0.07 | -0.15 |
| SF-12 Mental | post | 40.04 | 41.27 | 12.64 | 12.33 | 342 | 110 | 12.57 | -0.12 | 0.31 | 0.10 |
| ISMI Total (No Res) | pre | 1.91 | 1.99 | 0.50 | 0.50 | 342 | 110 | 0.50 | -0.04 | 0.39 | 0.17 |
| ISMI Total (No Res) | post | 1.77 | 1.74 | 0.59 | 0.58 | 342 | 110 | 0.59 | -0.27 | 0.16 | -0.05 |
| IASMHS Total | pre | 66.52 | 63.79 | 12.20 | 12.20 | 342 | 110 | 12.20 | -0.44 | -0.01 | -0.22 |
| IASMHS Total | post | 69.02 | 66.89 | 14.41 | 14.15 | 342 | 110 | 14.35 | -0.36 | 0.07 | -0.15 |
| LPFS Total | pre | 28.59 | 29.01 | 5.81 | 5.81 | 342 | 110 | 5.81 | -0.14 | 0.29 | 0.07 |
| LPFS Total | post | 27.75 | 27.44 | 6.86 | 6.71 | 342 | 110 | 6.82 | -0.26 | 0.17 | -0.04 |
| LPFS Self | pre | 15.86 | 16.38 | 3.48 | 3.48 | 342 | 110 | 3.48 | -0.07 | 0.36 | 0.15 |
| LPFS Self | post | 15.20 | 15.08 | 4.16 | 4.07 | 342 | 110 | 4.14 | -0.24 | 0.19 | -0.03 |
| LPFS Interpersonal | pre | 12.73 | 12.63 | 3.28 | 3.28 | 342 | 110 | 3.28 | -0.25 | 0.18 | -0.03 |
| LPFS Interpersonal | post | 12.54 | 12.35 | 3.95 | 3.86 | 342 | 110 | 3.92 | -0.26 | 0.17 | -0.05 |

Note: emmean_g0 = estimated mean group 0; emmean_g1 = estimated mean group 1; group 0 = preference matched; group 3 = preference not matched; SD = standard deviation calculated with standard error and n; sd_pooled = pooled standard deviation; d_lower = lower end of 95% confidence interval; d_upper = upper end of 95% confidence_interval; d = Cohen’s d

Standard deviations were derived from model-based standard errors (SD = SE × √n) rather than observed sample standard deviations, as the estimated marginal means from the mixed-effects models do not have directly corresponding observed SDs. Cohen's d was computed from these model-derived pooled SDs.

Appendix E.4

Effect sizes Cohen’s d for between-group effect sizes in the preference matched vs. not matched detailed conditions for pre-post

| outcome | Time-  point | Emm  g0 | Emm  g5 | Emm  g6 | SD_g0 | SD_g5 | SD_g6 | n_g0 | n_g5 | n_g6 | sdp  5_0 | lower  5_0 | upper  5_0 | d_5_0 | sdp  6_0 | lower  6_0 | upper  6_0 | d_6_0 | sdp  6_5 | lower  6_5 | upper  6_5 | d_6_5 |
| --- | --- | --- | --- | --- | --- | --- | --- | --- | --- | --- | --- | --- | --- | --- | --- | --- | --- | --- | --- | --- | --- | --- |
| SPS | pre | 36.31 | 37.93 | 37.21 | 13.07 | 13.07 | 13.07 | 342 | 29 | 81 | 13.07 | -0.36 | 0.40 | 0.12 | 13.07 | -0.23 | 0.25 | 0.07 | 13.07 | -0.44 | 0.41 | -0.06 |
| SPS | post | 26.92 | 25.95 | 27.03 | 16.18 | 15.84 | 15.68 | 342 | 29 | 81 | 16.15 | -0.39 | 0.37 | -0.06 | 16.08 | -0.24 | 0.24 | 0.01 | 15.72 | -0.41 | 0.44 | 0.07 |
| SIAS | pre | 48.97 | 48.90 | 48.38 | 12.19 | 12.19 | 12.19 | 342 | 29 | 81 | 12.19 | -0.38 | 0.38 | -0.01 | 12.19 | -0.25 | 0.24 | -0.05 | 12.19 | -0.43 | 0.42 | -0.04 |
| SIAS | post | 39.40 | 37.48 | 38.39 | 15.30 | 14.97 | 14.81 | 342 | 29 | 81 | 15.28 | -0.40 | 0.35 | -0.13 | 15.21 | -0.25 | 0.23 | -0.07 | 14.85 | -0.41 | 0.44 | 0.06 |
| SA composite | pre | -0.01 | 0.05 | <.001 | 0.94 | 0.94 | 0.94 | 342 | 29 | 81 | 0.94 | -0.37 | 0.39 | 0.06 | 0.94 | -0.24 | 0.24 | 0.01 | 0.94 | -0.44 | 0.41 | -0.05 |
| SA composite | post | -0.79 | -0.91 | -0.83 | 1.17 | 1.15 | 1.13 | 342 | 29 | 81 | 1.17 | -0.40 | 0.36 | -0.10 | 1.16 | -0.25 | 0.24 | -0.03 | 1.14 | -0.41 | 0.44 | 0.07 |
| PHQ-9 | pre | 9.15 | 8.48 | 9.19 | 4.82 | 4.82 | 4.82 | 342 | 29 | 81 | 4.82 | -0.41 | 0.35 | -0.14 | 4.82 | -0.24 | 0.24 | 0.01 | 4.82 | -0.39 | 0.46 | 0.15 |
| PHQ-9 | post | 8.21 | 6.91 | 7.88 | 6.06 | 5.92 | 5.86 | 342 | 29 | 81 | 6.05 | -0.42 | 0.34 | -0.21 | 6.02 | -0.25 | 0.24 | -0.06 | 5.88 | -0.39 | 0.46 | 0.16 |
| SF-12 Physical | pre | 53.34 | 51.96 | 51.71 | 6.98 | 6.98 | 6.98 | 342 | 29 | 81 | 6.98 | -0.42 | 0.34 | -0.20 | 6.98 | -0.27 | 0.21 | -0.23 | 6.98 | -0.43 | 0.42 | -0.04 |
| SF-12 Physical | post | 51.64 | 51.54 | 50.85 | 8.87 | 8.67 | 8.64 | 342 | 29 | 81 | 8.85 | -0.38 | 0.38 | -0.01 | 8.83 | -0.25 | 0.23 | -0.09 | 8.65 | -0.44 | 0.41 | -0.08 |
| SF-12  Mental | pre | 36.87 | 35.72 | 35.26 | 9.98 | 9.98 | 9.98 | 342 | 29 | 81 | 9.98 | -0.40 | 0.36 | -0.11 | 9.98 | -0.26 | 0.22 | -0.16 | 9.98 | -0.43 | 0.41 | -0.05 |
| SF-12  Mental | post | 40.04 | 42.49 | 40.84 | 12.66 | 12.37 | 12.34 | 342 | 29 | 81 | 12.64 | -0.34 | 0.42 | 0.19 | 12.60 | -0.23 | 0.25 | 0.06 | 12.35 | -0.45 | 0.40 | -0.13 |
| ISMI Total NR | pre | 1.91 | 1.89 | 2.03 | 0.50 | 0.50 | 0.50 | 342 | 29 | 81 | 0.50 | -0.39 | 0.37 | -0.03 | 0.50 | -0.21 | 0.27 | 0.25 | 0.50 | -0.36 | 0.48 | 0.28 |
| ISMI Total NR | post | 1.77 | 1.68 | 1.77 | 0.59 | 0.58 | 0.58 | 342 | 29 | 81 | 0.59 | -0.41 | 0.35 | -0.16 | 0.59 | -0.24 | 0.24 | -0.01 | 0.58 | -0.39 | 0.46 | 0.15 |
| IASMHS Total | pre | 66.52 | 63.07 | 64.05 | 12.22 | 12.22 | 12.22 | 342 | 29 | 81 | 12.22 | -0.43 | 0.32 | -0.28 | 12.22 | -0.27 | 0.22 | -0.20 | 12.22 | -0.41 | 0.44 | 0.08 |
| IASMHS Total | post | 69.02 | 68.40 | 66.35 | 14.42 | 14.18 | 14.15 | 342 | 29 | 81 | 14.40 | -0.39 | 0.37 | -0.04 | 14.37 | -0.27 | 0.22 | -0.19 | 14.15 | -0.46 | 0.39 | -0.14 |
| LPFS Total | pre | 28.59 | 28.72 | 29.11 | 5.82 | 5.82 | 5.82 | 342 | 29 | 81 | 5.82 | -0.37 | 0.38 | 0.02 | 5.82 | -0.23 | 0.25 | 0.09 | 5.82 | -0.41 | 0.44 | 0.07 |
| LPFS Total | post | 27.75 | 26.69 | 27.71 | 6.87 | 6.73 | 6.72 | 342 | 29 | 81 | 6.86 | -0.41 | 0.35 | -0.15 | 6.84 | -0.24 | 0.24 | -0.01 | 6.72 | -0.39 | 0.46 | 0.15 |
| LPFS Self | pre | 15.86 | 16.17 | 16.46 | 3.48 | 3.48 | 3.48 | 342 | 29 | 81 | 3.48 | -0.36 | 0.40 | 0.09 | 3.48 | -0.22 | 0.26 | 0.17 | 3.48 | -0.41 | 0.44 | 0.08 |
| LPFS Self | post | 15.20 | 14.44 | 15.31 | 4.16 | 4.08 | 4.07 | 342 | 29 | 81 | 4.16 | -0.41 | 0.34 | -0.18 | 4.15 | -0.24 | 0.25 | 0.03 | 4.07 | -0.38 | 0.47 | 0.21 |
| LPFS Inter-personal | pre | 12.73 | 12.55 | 12.65 | 3.29 | 3.29 | 3.29 | 342 | 29 | 81 | 3.29 | -0.39 | 0.37 | -0.05 | 3.29 | -0.25 | 0.24 | -0.02 | 3.29 | -0.42 | 0.43 | 0.03 |
| LPFS Inter-personal | post | 12.54 | 12.23 | 12.39 | 3.95 | 3.87 | 3.86 | 342 | 29 | 81 | 3.95 | -0.39 | 0.36 | -0.08 | 3.93 | -0.25 | 0.24 | -0.04 | 3.86 | -0.42 | 0.43 | 0.04 |

Note: Emm g0 = estimated mean group 0; Emm g5 = estimated mean group 5; Emm g6 = estimated mean group 6; group 0 = preference matched; group 5 = preference for anon not matched; group 6 = preference for non-anon not matched; SD = standard deviation calculated with standard error and n; sdp = pooled standard deviation; lower = lower end of 95% confidence interval; upper = upper end of 95% confidence_interval; d = Cohen’s d

Standard deviations were derived from model-based standard errors (SD = SE × √n) rather than observed sample standard deviations, as the estimated marginal means from the mixed-effects models do not have directly corresponding observed SDs. Cohen's d was computed from these model-derived pooled SDs.

Appendix F.1

Effect sizes Cohen’s d for between-group effect sizes in the randomized arms for pre-post-followup

| outcome | timepoint | emmean_g1 | emmean_g3 | SD_g1 | SD_g3 | n_g1 | n_g3 | sd_pooled | d_lower | d_upper | d |
| --- | --- | --- | --- | --- | --- | --- | --- | --- | --- | --- | --- |
| SPS | pre | 36.74 | 38.71 | 13.65 | 13.65 | 113 | 114 | 13.65 | -0.12 | 0.40 | 0.14 |
| SPS | post | 27.40 | 29.20 | 15.98 | 16.97 | 113 | 114 | 16.48 | -0.15 | 0.37 | 0.11 |
| SPS | followup | 24.03 | 27.75 | 16.93 | 17.47 | 113 | 114 | 17.20 | -0.04 | 0.48 | 0.22 |
| SIAS | pre | 48.32 | 50.26 | 13.07 | 13.07 | 113 | 114 | 13.07 | -0.11 | 0.41 | 0.15 |
| SIAS | post | 38.52 | 40.61 | 15.46 | 16.47 | 113 | 114 | 15.97 | -0.13 | 0.39 | 0.13 |
| SIAS | followup | 36.31 | 39.58 | 16.43 | 16.98 | 113 | 114 | 16.71 | -0.07 | 0.46 | 0.20 |
| SA composite | pre | -0.02 | 0.14 | 1.01 | 1.01 | 113 | 114 | 1.01 | -0.10 | 0.42 | 0.16 |
| SA composite | post | -0.81 | -0.65 | 1.19 | 1.27 | 113 | 114 | 1.23 | -0.13 | 0.39 | 0.13 |
| SA composite | followup | -1.03 | -0.75 | 1.26 | 1.31 | 113 | 114 | 1.29 | -0.04 | 0.48 | 0.22 |
| PHQ-9 | pre | 9.19 | 9.89 | 5.14 | 5.14 | 113 | 114 | 5.14 | -0.13 | 0.39 | 0.13 |
| PHQ-9 | post | 8.18 | 9.13 | 6.22 | 6.68 | 113 | 114 | 6.46 | -0.11 | 0.41 | 0.15 |
| PHQ-9 | followup | 7.79 | 9.61 | 6.71 | 6.91 | 113 | 114 | 6.81 | 0.01 | 0.53 | 0.27 |
| SF-12 Physical | pre | 51.78 | 52.93 | 7.11 | 7.11 | 113 | 114 | 7.11 | -0.10 | 0.42 | 0.16 |
| SF-12 Physical | post | 50.62 | 51.77 | 8.95 | 9.63 | 113 | 114 | 9.30 | -0.14 | 0.38 | 0.12 |
| SF-12 Physical | followup | 51.96 | 51.34 | 9.71 | 10.02 | 113 | 114 | 9.87 | -0.32 | 0.20 | -0.06 |
| SF-12 Mental | pre | 35.26 | 34.64 | 10.39 | 10.39 | 113 | 114 | 10.39 | -0.32 | 0.20 | -0.06 |
| SF-12 Mental | post | 39.91 | 38.73 | 12.99 | 13.97 | 113 | 114 | 13.49 | -0.35 | 0.17 | -0.09 |
| SF-12 Mental | followup | 39.48 | 37.31 | 14.07 | 14.51 | 113 | 114 | 14.29 | -0.41 | 0.11 | -0.15 |
| ISMI Total (No Res) | pre | 1.98 | 1.97 | 0.50 | 0.50 | 113 | 114 | 0.50 | -0.27 | 0.25 | -0.01 |
| ISMI Total (No Res) | post | 1.77 | 1.82 | 0.58 | 0.61 | 113 | 114 | 0.59 | -0.18 | 0.34 | 0.08 |
| ISMI Total (No Res) | followup | 1.77 | 1.86 | 0.61 | 0.63 | 113 | 114 | 0.62 | -0.12 | 0.40 | 0.14 |
| IASMHS Total | pre | 65.32 | 64.25 | 12.41 | 12.41 | 113 | 114 | 12.41 | -0.35 | 0.17 | -0.09 |
| IASMHS Total | post | 66.87 | 68.59 | 14.39 | 15.15 | 113 | 114 | 14.78 | -0.14 | 0.38 | 0.12 |
| IASMHS Total | followup | 68.82 | 65.93 | 15.28 | 15.66 | 113 | 114 | 15.47 | -0.45 | 0.07 | -0.19 |
| LPFS Total | pre | 28.71 | 29.46 | 5.75 | 5.75 | 113 | 114 | 5.75 | -0.13 | 0.39 | 0.13 |
| LPFS Total | post | 27.27 | 28.46 | 6.71 | 7.11 | 113 | 114 | 6.91 | -0.09 | 0.43 | 0.17 |
| LPFS Total | followup | 26.41 | 28.77 | 7.13 | 7.31 | 113 | 114 | 7.22 | 0.06 | 0.59 | 0.33 |
| LPFS Self | pre | 16.23 | 16.10 | 3.57 | 3.57 | 113 | 114 | 3.57 | -0.30 | 0.22 | -0.04 |
| LPFS Self | post | 15.17 | 15.38 | 4.16 | 4.40 | 113 | 114 | 4.28 | -0.21 | 0.31 | 0.05 |
| LPFS Self | followup | 14.46 | 15.53 | 4.42 | 4.53 | 113 | 114 | 4.47 | -0.02 | 0.50 | 0.24 |
| LPFS Interpersonal | pre | 12.48 | 13.36 | 3.19 | 3.19 | 113 | 114 | 3.19 | 0.01 | 0.54 | 0.28 |
| LPFS Interpersonal | post | 12.11 | 13.08 | 3.75 | 3.98 | 113 | 114 | 3.87 | -0.01 | 0.51 | 0.25 |
| LPFS Interpersonal | followup | 11.95 | 13.23 | 4.00 | 4.10 | 113 | 114 | 4.05 | 0.06 | 0.58 | 0.32 |

Note: emmean_g1 = estimated mean group 1; emmean_g3 = estimated mean group 3; group 1 = random anon; group 3 = random non-anon; SD = standard deviation calculated with standard error and n; sd_pooled = pooled standard deviation; d_lower = lower end of 95% confidence interval; d_upper = upper end of 95% confidence_interval; d = Cohen’s d

Standard deviations were derived from model-based standard errors (SD = SE × √n) rather than observed sample standard deviations, as the estimated marginal means from the mixed-effects models do not have directly corresponding observed SDs. Cohen's d was computed from these model-derived pooled SDs.

Appendix F.2

Effect sizes Cohen’s d for between-group effect sizes in the preference arms for pre-post-followup

| outcome | timepoint | emmean_g2 | emmean_g4 | SD_g2 | SD_g4 | n_g2 | n_g4 | sd_pooled | d_lower | d_upper | d |
| --- | --- | --- | --- | --- | --- | --- | --- | --- | --- | --- | --- |
| SPS | pre | 34.03 | 35.96 | 12.35 | 12.35 | 64 | 161 | 12.35 | -0.13 | 0.45 | 0.16 |
| SPS | post | 23.67 | 26.36 | 14.57 | 15.53 | 64 | 161 | 15.26 | -0.11 | 0.47 | 0.18 |
| SPS | followup | 21.82 | 23.46 | 16.22 | 17.06 | 64 | 161 | 16.83 | -0.19 | 0.39 | 0.10 |
| SIAS | pre | 48.94 | 48.22 | 11.77 | 11.77 | 64 | 161 | 11.77 | -0.35 | 0.23 | -0.06 |
| SIAS | post | 40.16 | 38.13 | 13.71 | 14.56 | 64 | 161 | 14.33 | -0.43 | 0.15 | -0.14 |
| SIAS | followup | 37.79 | 35.40 | 15.16 | 15.91 | 64 | 161 | 15.70 | -0.44 | 0.14 | -0.15 |
| SA composite | pre | -0.10 | -0.05 | 0.89 | 0.89 | 64 | 161 | 0.89 | -0.24 | 0.34 | 0.05 |
| SA composite | post | -0.88 | -0.86 | 1.04 | 1.10 | 64 | 161 | 1.09 | -0.28 | 0.30 | 0.01 |
| SA composite | followup | -1.06 | -1.09 | 1.15 | 1.21 | 64 | 161 | 1.19 | -0.32 | 0.26 | -0.03 |
| PHQ-9 | pre | 8.44 | 8.77 | 4.74 | 4.74 | 64 | 161 | 4.74 | -0.22 | 0.36 | 0.07 |
| PHQ-9 | post | 6.98 | 7.88 | 5.53 | 5.87 | 64 | 161 | 5.78 | -0.14 | 0.44 | 0.15 |
| PHQ-9 | followup | 7.99 | 7.67 | 6.11 | 6.42 | 64 | 161 | 6.33 | -0.34 | 0.24 | -0.05 |
| SF-12 Physical | pre | 54.20 | 53.32 | 6.96 | 6.96 | 64 | 161 | 6.96 | -0.42 | 0.16 | -0.13 |
| SF-12 Physical | post | 52.74 | 51.48 | 8.23 | 8.78 | 64 | 161 | 8.63 | -0.44 | 0.14 | -0.15 |
| SF-12 Physical | followup | 54.23 | 52.39 | 9.18 | 9.67 | 64 | 161 | 9.53 | -0.48 | 0.10 | -0.19 |
| SF-12 Mental | pre | 39.11 | 37.66 | 10.02 | 10.02 | 64 | 161 | 10.02 | -0.44 | 0.14 | -0.15 |
| SF-12 Mental | post | 42.88 | 40.52 | 11.99 | 12.84 | 64 | 161 | 12.61 | -0.48 | 0.10 | -0.19 |
| SF-12 Mental | followup | 41.48 | 41.24 | 13.47 | 14.21 | 64 | 161 | 14.00 | -0.31 | 0.27 | -0.02 |
| ISMI Total (No Res) | pre | 1.84 | 1.90 | 0.51 | 0.51 | 64 | 161 | 0.51 | -0.17 | 0.41 | 0.12 |
| ISMI Total (No Res) | post | 1.70 | 1.76 | 0.56 | 0.59 | 64 | 161 | 0.58 | -0.20 | 0.38 | 0.09 |
| ISMI Total (No Res) | followup | 1.63 | 1.71 | 0.61 | 0.63 | 64 | 161 | 0.63 | -0.15 | 0.43 | 0.14 |
| IASMHS Total | pre | 66.30 | 67.20 | 12.26 | 12.26 | 64 | 161 | 12.26 | -0.22 | 0.36 | 0.07 |
| IASMHS Total | post | 67.42 | 70.10 | 13.47 | 14.01 | 64 | 161 | 13.86 | -0.10 | 0.48 | 0.19 |
| IASMHS Total | followup | 69.40 | 71.45 | 14.46 | 14.90 | 64 | 161 | 14.78 | -0.15 | 0.43 | 0.14 |
| LPFS Total | pre | 27.53 | 28.61 | 5.97 | 5.97 | 64 | 161 | 5.97 | -0.11 | 0.47 | 0.18 |
| LPFS Total | post | 27.03 | 27.68 | 6.74 | 7.03 | 64 | 161 | 6.95 | -0.20 | 0.38 | 0.09 |
| LPFS Total | followup | 26.71 | 27.31 | 7.32 | 7.58 | 64 | 161 | 7.51 | -0.21 | 0.37 | 0.08 |
| LPFS Self | pre | 14.81 | 16.21 | 3.43 | 3.43 | 64 | 161 | 3.43 | 0.12 | 0.70 | 0.41 |
| LPFS Self | post | 14.33 | 15.35 | 3.95 | 4.15 | 64 | 161 | 4.09 | -0.04 | 0.54 | 0.25 |
| LPFS Self | followup | 14.51 | 15.16 | 4.34 | 4.51 | 64 | 161 | 4.46 | -0.15 | 0.43 | 0.14 |
| LPFS Interpersonal | pre | 12.72 | 12.40 | 3.38 | 3.38 | 64 | 161 | 3.38 | -0.38 | 0.19 | -0.10 |
| LPFS Interpersonal | post | 12.70 | 12.29 | 3.89 | 4.07 | 64 | 161 | 4.02 | -0.39 | 0.19 | -0.10 |
| LPFS Interpersonal | followup | 12.19 | 12.12 | 4.26 | 4.43 | 64 | 161 | 4.38 | -0.31 | 0.27 | -0.02 |

Note: emmean_g2 = estimated mean group 2; emmean_g4 = estimated mean group 4; group 2 = prefer anon; group 4 = prefer non-anon; SD = standard deviation calculated with standard error and n; sd_pooled = pooled standard deviation; d_lower = lower end of 95% confidence interval; d_upper = upper end of 95% confidence_interval; d = Cohen’s d

Standard deviations were derived from model-based standard errors (SD = SE × √n) rather than observed sample standard deviations, as the estimated marginal means from the mixed-effects models do not have directly corresponding observed SDs. Cohen's d was computed from these model-derived pooled SDs.

Appendix F.3

Effect sizes Cohen’s d for between-group effect sizes in the preference matched vs. not matched conditions for pre-post-followup

| outcome | timepoint | emmean_g0 | emmean_g1 | SD_g0 | SD_g1 | n_g0 | n_g1 | sd_pooled | d_lower | d_upper | d |
| --- | --- | --- | --- | --- | --- | --- | --- | --- | --- | --- | --- |
| SPS | pre | 36.31 | 37.40 | 13.12 | 13.12 | 342 | 110 | 13.12 | -0.13 | 0.30 | 0.08 |
| SPS | post | 27.03 | 26.82 | 16.02 | 15.54 | 342 | 110 | 15.91 | -0.23 | 0.20 | -0.01 |
| SPS | followup | 24.67 | 24.09 | 17.33 | 16.19 | 342 | 110 | 17.06 | -0.25 | 0.18 | -0.03 |
| SIAS | pre | 48.97 | 48.52 | 12.45 | 12.45 | 342 | 110 | 12.45 | -0.25 | 0.18 | -0.04 |
| SIAS | post | 39.53 | 38.13 | 15.24 | 14.78 | 342 | 110 | 15.13 | -0.31 | 0.12 | -0.09 |
| SIAS | followup | 37.57 | 35.69 | 16.50 | 15.41 | 342 | 110 | 16.24 | -0.33 | 0.10 | -0.12 |
| SA composite | pre | -0.01 | 0.02 | 0.96 | 0.96 | 342 | 110 | 0.96 | -0.19 | 0.24 | 0.02 |
| SA composite | post | -0.78 | -0.85 | 1.17 | 1.13 | 342 | 110 | 1.16 | -0.27 | 0.16 | -0.06 |
| SA composite | followup | -0.95 | -1.06 | 1.26 | 1.18 | 342 | 110 | 1.24 | -0.30 | 0.13 | -0.08 |
| PHQ-9 | pre | 9.15 | 9.00 | 4.96 | 4.96 | 342 | 110 | 4.96 | -0.24 | 0.19 | -0.03 |
| PHQ-9 | post | 8.31 | 7.61 | 6.16 | 5.97 | 342 | 110 | 6.12 | -0.33 | 0.10 | -0.11 |
| PHQ-9 | followup | 8.66 | 7.12 | 6.70 | 6.28 | 342 | 110 | 6.60 | -0.45 | -0.02 | -0.23 |
| SF-12 Physical | pre | 53.34 | 51.77 | 7.02 | 7.02 | 342 | 110 | 7.02 | -0.44 | -0.01 | -0.22 |
| SF-12 Physical | post | 51.60 | 51.10 | 9.00 | 8.74 | 342 | 110 | 8.93 | -0.27 | 0.16 | -0.06 |
| SF-12 Physical | followup | 52.74 | 50.95 | 9.89 | 9.20 | 342 | 110 | 9.73 | -0.40 | 0.03 | -0.18 |
| SF-12 Mental | pre | 36.87 | 35.38 | 10.29 | 10.29 | 342 | 110 | 10.29 | -0.36 | 0.07 | -0.14 |
| SF-12 Mental | post | 39.95 | 41.11 | 13.13 | 12.77 | 342 | 110 | 13.05 | -0.13 | 0.30 | 0.09 |
| SF-12 Mental | followup | 39.13 | 41.51 | 14.43 | 13.43 | 342 | 110 | 14.19 | -0.05 | 0.38 | 0.17 |
| ISMI Total (No Res) | pre | 1.91 | 1.99 | 0.50 | 0.50 | 342 | 110 | 0.50 | -0.04 | 0.39 | 0.17 |
| ISMI Total (No Res) | post | 1.78 | 1.75 | 0.59 | 0.58 | 342 | 110 | 0.59 | -0.27 | 0.16 | -0.06 |
| ISMI Total (No Res) | followup | 1.76 | 1.75 | 0.63 | 0.60 | 342 | 110 | 0.62 | -0.23 | 0.20 | -0.02 |
| IASMHS Total | pre | 66.52 | 63.79 | 12.35 | 12.35 | 342 | 110 | 12.35 | -0.44 | -0.01 | -0.22 |
| IASMHS Total | post | 68.88 | 67.02 | 14.40 | 14.13 | 342 | 110 | 14.34 | -0.34 | 0.09 | -0.13 |
| IASMHS Total | followup | 69.42 | 67.90 | 15.39 | 14.73 | 342 | 110 | 15.23 | -0.31 | 0.12 | -0.10 |
| LPFS Total | pre | 28.59 | 29.01 | 5.88 | 5.88 | 342 | 110 | 5.88 | -0.14 | 0.29 | 0.07 |
| LPFS Total | post | 27.80 | 27.35 | 6.97 | 6.80 | 342 | 110 | 6.93 | -0.28 | 0.15 | -0.06 |
| LPFS Total | followup | 27.57 | 26.80 | 7.46 | 7.12 | 342 | 110 | 7.38 | -0.32 | 0.11 | -0.10 |
| LPFS Self | pre | 15.86 | 16.38 | 3.52 | 3.52 | 342 | 110 | 3.52 | -0.07 | 0.36 | 0.15 |
| LPFS Self | post | 15.22 | 15.04 | 4.21 | 4.11 | 342 | 110 | 4.19 | -0.26 | 0.17 | -0.04 |
| LPFS Self | followup | 15.13 | 14.60 | 4.53 | 4.31 | 342 | 110 | 4.48 | -0.33 | 0.10 | -0.12 |
| LPFS Interpersonal | pre | 12.73 | 12.63 | 3.30 | 3.30 | 342 | 110 | 3.30 | -0.25 | 0.18 | -0.03 |
| LPFS Interpersonal | post | 12.57 | 12.30 | 3.97 | 3.87 | 342 | 110 | 3.95 | -0.28 | 0.15 | -0.07 |
| LPFS Interpersonal | followup | 12.43 | 12.18 | 4.28 | 4.07 | 342 | 110 | 4.23 | -0.28 | 0.15 | -0.06 |

Note: emmean_g0 = estimated mean group 0; emmean_g1 = estimated mean group 1; group 0 = preference matched; group 3 = preference not matched; SD = standard deviation calculated with standard error and n; sd_pooled = pooled standard deviation; d_lower = lower end of 95% confidence interval; d_upper = upper end of 95% confidence_interval; d = Cohen’s d

Standard deviations were derived from model-based standard errors (SD = SE × √n) rather than observed sample standard deviations, as the estimated marginal means from the mixed-effects models do not have directly corresponding observed SDs. Cohen's d was computed from these model-derived pooled SDs.

Appendix F.4

Effect sizes Cohen’s d for between-group effect sizes in the preference matched vs. not matched detailed conditions for pre-post-followup

| outcome | Time  point | Emm  g0 | Emm  g5 | Emm  g6 | SD  g0 | SD  g5 | SD  g6 | n_g0 | n_g5 | n_g6 | sdp  5_0 | lower  5_0 | upper  5_0 | d  5_0 | sdp  6_0 | lower  6_0 | upper  6_0 | d  6_0 | sdp  6_5 | lower  6_5 | upper  6_5 | d  6_5 |
| --- | --- | --- | --- | --- | --- | --- | --- | --- | --- | --- | --- | --- | --- | --- | --- | --- | --- | --- | --- | --- | --- | --- |
| SPS | pre | 36.31 | 37.93 | 37.21 | 13.13 | 13.13 | 13.13 | 342 | 29 | 81 | 13.13 | -0.36 | 0.40 | 0.12 | 13.13 | -0.23 | 0.25 | 0.07 | 13.13 | -0.44 | 0.41 | -0.05 |
| SPS | post | 27.03 | 26.40 | 26.95 | 16.04 | 15.63 | 15.54 | 342 | 29 | 81 | 16.01 | -0.39 | 0.37 | -0.04 | 15.95 | -0.24 | 0.24 | 0.00 | 15.57 | -0.42 | 0.43 | 0.04 |
| SPS | followup | 24.67 | 25.02 | 23.71 | 17.35 | 15.63 | 16.45 | 342 | 29 | 81 | 17.23 | -0.38 | 0.38 | 0.02 | 17.19 | -0.25 | 0.24 | -0.06 | 16.24 | -0.44 | 0.41 | -0.08 |
| SIAS | pre | 48.97 | 48.90 | 48.38 | 12.47 | 12.47 | 12.47 | 342 | 29 | 81 | 12.47 | -0.38 | 0.38 | -0.01 | 12.47 | -0.25 | 0.24 | -0.05 | 12.47 | -0.43 | 0.42 | -0.04 |
| SIAS | post | 39.53 | 38.08 | 38.16 | 15.26 | 14.87 | 14.79 | 342 | 29 | 81 | 15.23 | -0.40 | 0.36 | -0.10 | 15.17 | -0.25 | 0.23 | -0.09 | 14.81 | -0.42 | 0.43 | 0.01 |
| SIAS | followup | 37.57 | 35.31 | 35.86 | 16.52 | 14.87 | 15.66 | 342 | 29 | 81 | 16.40 | -0.41 | 0.35 | -0.14 | 16.36 | -0.26 | 0.23 | -0.10 | 15.46 | -0.42 | 0.43 | 0.04 |
| SA composite | pre | -0.01 | 0.05 | 0.00 | 0.96 | 0.96 | 0.96 | 342 | 29 | 81 | 0.96 | -0.37 | 0.39 | 0.06 | 0.96 | -0.24 | 0.24 | 0.01 | 0.96 | -0.44 | 0.41 | -0.05 |
| SA composite | post | -0.78 | -0.86 | -0.84 | 1.17 | 1.14 | 1.13 | 342 | 29 | 81 | 1.17 | -0.39 | 0.36 | -0.07 | 1.16 | -0.25 | 0.24 | -0.05 | 1.13 | -0.42 | 0.43 | 0.02 |
| SA composite | followup | -0.95 | -1.04 | -1.06 | 1.26 | 1.14 | 1.20 | 342 | 29 | 81 | 1.26 | -0.39 | 0.37 | -0.07 | 1.25 | -0.25 | 0.23 | -0.09 | 1.18 | -0.43 | 0.42 | -0.02 |
| PHQ-9 | pre | 9.15 | 8.48 | 9.19 | 4.97 | 4.97 | 4.97 | 342 | 29 | 81 | 4.97 | -0.40 | 0.35 | -0.13 | 4.97 | -0.24 | 0.24 | 0.01 | 4.97 | -0.39 | 0.45 | 0.14 |
| PHQ-9 | post | 8.31 | 7.00 | 7.83 | 6.17 | 6.00 | 5.97 | 342 | 29 | 81 | 6.16 | -0.42 | 0.34 | -0.21 | 6.13 | -0.25 | 0.23 | -0.08 | 5.98 | -0.39 | 0.45 | 0.14 |
| PHQ-9 | followup | 8.66 | 7.05 | 7.12 | 6.71 | 6.00 | 6.40 | 342 | 29 | 81 | 6.66 | -0.43 | 0.33 | -0.24 | 6.65 | -0.27 | 0.21 | -0.23 | 6.30 | -0.42 | 0.43 | 0.01 |
| SF-12 Physical | pre | 53.34 | 51.96 | 51.71 | 7.03 | 7.03 | 7.03 | 342 | 29 | 81 | 7.03 | -0.42 | 0.34 | -0.20 | 7.03 | -0.27 | 0.21 | -0.23 | 7.03 | -0.43 | 0.42 | -0.04 |
| SF-12 Physical | post | 51.60 | 51.53 | 50.94 | 9.01 | 8.75 | 8.76 | 342 | 29 | 81 | 8.99 | -0.38 | 0.38 | -0.01 | 8.96 | -0.25 | 0.23 | -0.07 | 8.76 | -0.44 | 0.41 | -0.07 |
| SF-12 Physical | followup | 52.74 | 51.03 | 50.92 | 9.91 | 8.75 | 9.41 | 342 | 29 | 81 | 9.83 | -0.41 | 0.35 | -0.17 | 9.82 | -0.27 | 0.22 | -0.18 | 9.25 | -0.43 | 0.42 | -0.01 |
| SF-12 Mental | pre | 36.87 | 35.72 | 35.26 | 10.30 | 10.30 | 10.30 | 342 | 29 | 81 | 10.30 | -0.40 | 0.36 | -0.11 | 10.30 | -0.26 | 0.22 | -0.16 | 10.30 | -0.43 | 0.41 | -0.05 |
| SF-12 Mental | post | 39.95 | 41.94 | 40.80 | 13.15 | 12.78 | 12.79 | 342 | 29 | 81 | 13.12 | -0.35 | 0.41 | 0.15 | 13.08 | -0.23 | 0.25 | 0.07 | 12.79 | -0.44 | 0.40 | -0.09 |
| SF-12 Mental | followup | 39.13 | 42.71 | 41.01 | 14.45 | 12.78 | 13.73 | 342 | 29 | 81 | 14.33 | -0.33 | 0.43 | 0.25 | 14.32 | -0.23 | 0.26 | 0.13 | 13.49 | -0.45 | 0.40 | -0.13 |
| ISMI Total (No Res) | pre | 1.91 | 1.89 | 2.03 | 0.50 | 0.50 | 0.50 | 342 | 29 | 81 | 0.50 | -0.39 | 0.37 | -0.03 | 0.50 | -0.21 | 0.27 | 0.25 | 0.50 | -0.36 | 0.48 | 0.28 |
| ISMI Total (No Res) | post | 1.78 | 1.69 | 1.77 | 0.59 | 0.58 | 0.58 | 342 | 29 | 81 | 0.59 | -0.41 | 0.35 | -0.16 | 0.59 | -0.24 | 0.24 | -0.02 | 0.58 | -0.39 | 0.45 | 0.14 |
| ISMI Total (No Res) | followup | 1.76 | 1.73 | 1.75 | 0.63 | 0.58 | 0.61 | 342 | 29 | 81 | 0.63 | -0.39 | 0.37 | -0.05 | 0.63 | -0.24 | 0.24 | -0.01 | 0.60 | -0.42 | 0.43 | 0.04 |
| IASMHS Total | pre | 66.52 | 63.07 | 64.05 | 12.36 | 12.36 | 12.36 | 342 | 29 | 81 | 12.36 | -0.43 | 0.33 | -0.28 | 12.36 | -0.27 | 0.22 | -0.20 | 12.36 | -0.41 | 0.44 | 0.08 |
| IASMHS Total | post | 68.88 | 68.58 | 66.47 | 14.41 | 14.14 | 14.13 | 342 | 29 | 81 | 14.39 | -0.38 | 0.37 | -0.02 | 14.36 | -0.26 | 0.22 | -0.17 | 14.13 | -0.46 | 0.39 | -0.15 |
| IASMHS Total | followup | 69.42 | 66.30 | 68.58 | 15.39 | 14.34 | 14.90 | 342 | 29 | 81 | 15.31 | -0.42 | 0.34 | -0.20 | 15.30 | -0.25 | 0.24 | -0.06 | 14.76 | -0.39 | 0.46 | 0.15 |
| LPFS Total | pre | 28.59 | 28.72 | 29.11 | 5.88 | 5.88 | 5.88 | 342 | 29 | 81 | 5.88 | -0.37 | 0.38 | 0.02 | 5.88 | -0.23 | 0.25 | 0.09 | 5.88 | -0.41 | 0.44 | 0.07 |
| LPFS Total | post | 27.80 | 26.61 | 27.61 | 6.97 | 6.81 | 6.80 | 342 | 29 | 81 | 6.96 | -0.41 | 0.35 | -0.17 | 6.94 | -0.25 | 0.24 | -0.03 | 6.80 | -0.39 | 0.46 | 0.15 |
| LPFS Total | followup | 27.57 | 27.77 | 26.38 | 7.46 | 6.92 | 7.20 | 342 | 29 | 81 | 7.42 | -0.37 | 0.38 | 0.03 | 7.41 | -0.26 | 0.22 | -0.16 | 7.13 | -0.47 | 0.38 | -0.20 |
| LPFS Self | pre | 15.86 | 16.17 | 16.46 | 3.52 | 3.52 | 3.52 | 342 | 29 | 81 | 3.52 | -0.36 | 0.40 | 0.09 | 3.52 | -0.22 | 0.26 | 0.17 | 3.52 | -0.41 | 0.44 | 0.08 |
| LPFS Self | post | 15.22 | 14.37 | 15.28 | 4.22 | 4.11 | 4.11 | 342 | 29 | 81 | 4.21 | -0.42 | 0.34 | -0.20 | 4.20 | -0.24 | 0.24 | 0.01 | 4.11 | -0.38 | 0.47 | 0.22 |
| LPFS Self | followup | 15.13 | 14.85 | 14.47 | 4.53 | 4.18 | 4.37 | 342 | 29 | 81 | 4.50 | -0.39 | 0.37 | -0.06 | 4.50 | -0.26 | 0.22 | -0.15 | 4.32 | -0.44 | 0.41 | -0.09 |
| LPFS Interpersonal | pre | 12.73 | 12.55 | 12.65 | 3.31 | 3.31 | 3.31 | 342 | 29 | 81 | 3.31 | -0.39 | 0.37 | -0.05 | 3.31 | -0.25 | 0.24 | -0.02 | 3.31 | -0.42 | 0.43 | 0.03 |
| LPFS Interpersonal | post | 12.57 | 12.21 | 12.33 | 3.97 | 3.88 | 3.87 | 342 | 29 | 81 | 3.97 | -0.40 | 0.36 | -0.09 | 3.96 | -0.25 | 0.23 | -0.06 | 3.88 | -0.42 | 0.43 | 0.03 |
| LPFS Interpersonal | followup | 12.43 | 12.87 | 11.89 | 4.28 | 3.94 | 4.12 | 342 | 29 | 81 | 4.25 | -0.36 | 0.40 | 0.10 | 4.25 | -0.26 | 0.23 | -0.13 | 4.08 | -0.48 | 0.37 | -0.24 |

Note: Emm g0 = estimated mean group 0; Emm g5 = estimated mean group 5; Emm g6 = estimated mean group 6; group 0 = preference matched; group 5 = preference for anon not matched; group 6 = preference for non-anon not matched; SD = standard deviation calculated with standard error and n; sdp = pooled standard deviation; lower = lower end of 95% confidence interval; upper = upper end of 95% confidence_interval; d = Cohen’s d

Standard deviations were derived from model-based standard errors (SD = SE × √n) rather than observed sample standard deviations, as the estimated marginal means from the mixed-effects models do not have directly corresponding observed SDs. Cohen's d was computed from these model-derived pooled SDs.

Appendix G.1

Effect sizes Cohen’s d for within-group effect sizes in the randomized arms for pre-post

| outcome | group | n | emmean_pre | emmean_post | SD_pre | SD_post | d_lower | d_upper | d |
| --- | --- | --- | --- | --- | --- | --- | --- | --- | --- |
| SPS | 1 | 113 | 36.74 | 27.52 | 13.41 | 16.16 | -0.89 | -0.35 | -0.62 |
| SPS | 3 | 114 | 38.71 | 28.78 | 13.41 | 17.38 | -0.91 | -0.37 | -0.64 |
| SIAS | 1 | 113 | 48.32 | 38.72 | 12.60 | 15.35 | -0.95 | -0.41 | -0.68 |
| SIAS | 3 | 114 | 50.26 | 40.26 | 12.60 | 16.57 | -0.95 | -0.41 | -0.68 |
| SA composite | 1 | 113 | -0.02 | -0.79 | 0.98 | 1.19 | -0.98 | -0.44 | -0.71 |
| SA composite | 3 | 114 | 0.14 | -0.68 | 0.98 | 1.29 | -0.99 | -0.45 | -0.72 |
| PHQ-9 | 1 | 113 | 9.19 | 8.24 | 5.01 | 6.07 | -0.43 | 0.09 | -0.17 |
| PHQ-9 | 3 | 114 | 9.89 | 8.95 | 5.01 | 6.54 | -0.42 | 0.10 | -0.16 |
| SF-12 Physical | 1 | 113 | 51.78 | 50.59 | 7.04 | 8.76 | -0.41 | 0.11 | -0.15 |
| SF-12 Physical | 3 | 114 | 52.93 | 51.71 | 7.04 | 9.44 | -0.41 | 0.11 | -0.15 |
| SF-12 Mental | 1 | 113 | 35.26 | 39.90 | 9.95 | 12.50 | 0.15 | 0.67 | 0.41 |
| SF-12 Mental | 3 | 114 | 34.64 | 39.14 | 9.95 | 13.49 | 0.12 | 0.64 | 0.38 |
| ISMI Total (No Res) | 1 | 113 | 1.98 | 1.78 | 0.49 | 0.58 | -0.64 | -0.12 | -0.38 |
| ISMI Total (No Res) | 3 | 114 | 1.97 | 1.81 | 0.49 | 0.61 | -0.56 | -0.04 | -0.30 |
| IASMHS Total | 1 | 113 | 65.32 | 66.76 | 12.26 | 14.40 | -0.15 | 0.37 | 0.11 |
| IASMHS Total | 3 | 114 | 64.25 | 68.95 | 12.26 | 15.27 | 0.08 | 0.60 | 0.34 |
| LPFS Total | 1 | 113 | 28.71 | 27.35 | 5.70 | 6.69 | -0.48 | 0.04 | -0.22 |
| LPFS Total | 3 | 114 | 29.46 | 28.36 | 5.70 | 7.13 | -0.43 | 0.09 | -0.17 |
| LPFS Self | 1 | 113 | 16.23 | 15.19 | 3.52 | 4.12 | -0.53 | -0.01 | -0.27 |
| LPFS Self | 3 | 114 | 16.10 | 15.37 | 3.52 | 4.39 | -0.44 | 0.08 | -0.18 |
| LPFS Interpersonal | 1 | 113 | 12.48 | 12.15 | 3.21 | 3.79 | -0.35 | 0.17 | -0.09 |
| LPFS Interpersonal | 3 | 114 | 13.36 | 13.00 | 3.21 | 4.05 | -0.36 | 0.16 | -0.10 |

Note: group 1 = random anon; group 3 = random non-anon; emmean_pre = estimated mean at pre; emmean_post = estimated mean at post; SD_pre = standard deviation at pre calculated with standard error and n; SD_post = standard deviation at post calculated with standard error and n; d_lower = lower end of 95% confidence interval; d_upper = upper end of 95% confidence_interval; d = Cohen’s d

Standard deviations were derived from model-based standard errors (SD = SE × √n) rather than observed sample standard deviations, as the estimated marginal means from the mixed-effects models do not have directly corresponding observed SDs. Cohen's d was computed from these model-derived pooled SDs.

Appendix G.2

Effect sizes Cohen’s d for within-group effect sizes in the preference arms for pre-post

| outcome | group | n | emmean_pre | emmean_post | SD_pre | SD_post | d_lower | d_upper | d |
| --- | --- | --- | --- | --- | --- | --- | --- | --- | --- |
| SPS | 2 | 64 | 34.03 | 23.70 | 12.55 | 14.66 | -1.11 | -0.40 | -0.76 |
| SPS | 4 | 161 | 35.96 | 26.45 | 12.55 | 15.61 | -0.90 | -0.45 | -0.67 |
| SIAS | 2 | 64 | 48.94 | 40.15 | 11.74 | 13.82 | -1.04 | -0.33 | -0.69 |
| SIAS | 4 | 161 | 48.22 | 38.06 | 11.74 | 14.75 | -0.99 | -0.54 | -0.76 |
| SA composite | 2 | 64 | -0.10 | -0.88 | 0.89 | 1.04 | -1.17 | -0.45 | -0.81 |
| SA composite | 4 | 161 | -0.05 | -0.86 | 0.89 | 1.11 | -1.03 | -0.58 | -0.81 |
| PHQ-9 | 2 | 64 | 8.44 | 6.96 | 4.59 | 5.44 | -0.64 | 0.05 | -0.29 |
| PHQ-9 | 4 | 161 | 8.77 | 7.80 | 4.59 | 5.83 | -0.40 | 0.03 | -0.18 |
| SF-12 Physical | 2 | 64 | 54.20 | 52.74 | 6.92 | 8.18 | -0.54 | 0.15 | -0.19 |
| SF-12 Physical | 4 | 161 | 53.32 | 51.52 | 6.92 | 8.75 | -0.45 | -0.01 | -0.23 |
| SF-12 Mental | 2 | 64 | 39.11 | 42.78 | 9.80 | 11.57 | -0.01 | 0.69 | 0.34 |
| SF-12 Mental | 4 | 161 | 37.66 | 40.53 | 9.80 | 12.36 | 0.04 | 0.48 | 0.26 |
| ISMI Total (No Res) | 2 | 64 | 1.84 | 1.70 | 0.51 | 0.56 | -0.60 | 0.10 | -0.25 |
| ISMI Total (No Res) | 4 | 161 | 1.90 | 1.76 | 0.51 | 0.59 | -0.48 | -0.04 | -0.26 |
| IASMHS Total | 2 | 64 | 66.30 | 67.40 | 12.19 | 13.46 | -0.26 | 0.43 | 0.09 |
| IASMHS Total | 4 | 161 | 67.20 | 70.07 | 12.19 | 14.05 | 0.00 | 0.44 | 0.22 |
| LPFS Total | 2 | 64 | 27.53 | 27.07 | 5.90 | 6.58 | -0.42 | 0.27 | -0.07 |
| LPFS Total | 4 | 161 | 28.61 | 27.67 | 5.90 | 6.84 | -0.37 | 0.07 | -0.15 |
| LPFS Self | 2 | 64 | 14.81 | 14.36 | 3.41 | 3.90 | -0.47 | 0.22 | -0.12 |
| LPFS Self | 4 | 161 | 16.21 | 15.33 | 3.41 | 4.08 | -0.45 | -0.01 | -0.23 |
| LPFS Interpersonal | 2 | 64 | 12.72 | 12.71 | 3.33 | 3.80 | -0.35 | 0.34 | 0.00 |
| LPFS Interpersonal | 4 | 161 | 12.40 | 12.28 | 3.33 | 3.97 | -0.25 | 0.19 | -0.03 |

Note: group 2 = prefer anon; group 4 = prefer non-anon; emmean_pre = estimated mean at pre; emmean_post = estimated mean at post; SD_pre = standard deviation at pre calculated with standard error and n; SD_post = standard deviation at post calculated with standard error and n; d_lower = lower end of 95% confidence interval; d_upper = upper end of 95% confidence_interval; d = Cohen’s d

Standard deviations were derived from model-based standard errors (SD = SE × √n) rather than observed sample standard deviations, as the estimated marginal means from the mixed-effects models do not have directly corresponding observed SDs. Cohen's d was computed from these model-derived pooled SDs.

Appendix G.3

Effect sizes Cohen’s d for within-group effect sizes in the preference matched vs. not matched conditions for pre-post

| outcome | group | n | emmean_pre | emmean_post | SD_pre | SD_post | d_lower | d_upper | d |
| --- | --- | --- | --- | --- | --- | --- | --- | --- | --- |
| SPS | 0 | 342 | 36.31 | 26.92 | 13.05 | 16.16 | -0.79 | -0.49 | -0.64 |
| SPS | 1 | 110 | 37.40 | 26.75 | 13.05 | 15.70 | -1.01 | -0.46 | -0.74 |
| SIAS | 0 | 342 | 48.97 | 39.40 | 12.18 | 15.28 | -0.85 | -0.54 | -0.69 |
| SIAS | 1 | 110 | 48.52 | 38.16 | 12.18 | 14.83 | -1.04 | -0.49 | -0.76 |
| SA composite | 0 | 342 | -0.01 | -0.79 | 0.94 | 1.17 | -0.89 | -0.58 | -0.74 |
| SA composite | 1 | 110 | 0.02 | -0.85 | 0.94 | 1.14 | -1.10 | -0.55 | -0.83 |
| PHQ-9 | 0 | 342 | 9.15 | 8.21 | 4.82 | 6.05 | -0.32 | -0.02 | -0.17 |
| PHQ-9 | 1 | 110 | 9.00 | 7.63 | 4.82 | 5.87 | -0.52 | 0.01 | -0.26 |
| SF-12 Physical | 0 | 342 | 53.34 | 51.64 | 6.97 | 8.86 | -0.36 | -0.06 | -0.21 |
| SF-12 Physical | 1 | 110 | 51.77 | 51.03 | 6.97 | 8.64 | -0.36 | 0.17 | -0.09 |
| SF-12 Mental | 0 | 342 | 36.87 | 40.04 | 9.97 | 12.64 | 0.13 | 0.43 | 0.28 |
| SF-12 Mental | 1 | 110 | 35.38 | 41.27 | 9.97 | 12.33 | 0.26 | 0.79 | 0.53 |
| ISMI Total (No Res) | 0 | 342 | 1.91 | 1.77 | 0.50 | 0.59 | -0.40 | -0.10 | -0.25 |
| ISMI Total (No Res) | 1 | 110 | 1.99 | 1.74 | 0.50 | 0.58 | -0.73 | -0.19 | -0.46 |
| IASMHS Total | 0 | 342 | 66.52 | 69.02 | 12.20 | 14.41 | 0.04 | 0.34 | 0.19 |
| IASMHS Total | 1 | 110 | 63.79 | 66.89 | 12.20 | 14.15 | -0.03 | 0.50 | 0.23 |
| LPFS Total | 0 | 342 | 28.59 | 27.75 | 5.81 | 6.86 | -0.28 | 0.02 | -0.13 |
| LPFS Total | 1 | 110 | 29.01 | 27.44 | 5.81 | 6.71 | -0.51 | 0.02 | -0.25 |
| LPFS Self | 0 | 342 | 15.86 | 15.20 | 3.48 | 4.16 | -0.32 | -0.02 | -0.17 |
| LPFS Self | 1 | 110 | 16.38 | 15.08 | 3.48 | 4.07 | -0.61 | -0.08 | -0.34 |
| LPFS Interpersonal | 0 | 342 | 12.73 | 12.54 | 3.28 | 3.95 | -0.20 | 0.10 | -0.05 |
| LPFS Interpersonal | 1 | 110 | 12.63 | 12.35 | 3.28 | 3.86 | -0.34 | 0.19 | -0.08 |

Note: group 0 = preference matched; group 1 = preference not matched; emmean_pre = estimated mean at pre; emmean_post = estimated mean at post; SD_pre = standard deviation at pre calculated with standard error and n; SD_post = standard deviation at post calculated with standard error and n; d_lower = lower end of 95% confidence interval; d_upper = upper end of 95% confidence_interval; d = Cohen’s d

Standard deviations were derived from model-based standard errors (SD = SE × √n) rather than observed sample standard deviations, as the estimated marginal means from the mixed-effects models do not have directly corresponding observed SDs. Cohen's d was computed from these model-derived pooled SDs.

Appendix G.4

Effect sizes Cohen’s d for within-group effect sizes in the preference matched vs. not matched detailed conditions for pre-post

| outcome | group | n | emmean_pre | emmean_post | SD_pre | SD_post | d_lower | d_upper | d |
| --- | --- | --- | --- | --- | --- | --- | --- | --- | --- |
| SPS | 0 | 342 | 36.31 | 26.92 | 13.07 | 16.18 | -0.20 | 0.10 | -0.64 |
| SPS | 5 | 29 | 37.93 | 25.95 | 13.07 | 15.84 | -0.73 | 0.30 | -0.82 |
| SPS | 6 | 81 | 37.21 | 27.03 | 13.07 | 15.68 | -0.42 | 0.20 | -0.71 |
| SIAS | 0 | 342 | 48.97 | 39.40 | 12.19 | 15.30 | -0.20 | 0.10 | -0.69 |
| SIAS | 5 | 29 | 48.90 | 37.48 | 12.19 | 14.97 | -0.73 | 0.30 | -0.84 |
| SIAS | 6 | 81 | 48.38 | 38.39 | 12.19 | 14.81 | -0.42 | 0.19 | -0.74 |
| SA composite | 0 | 342 | -0.01 | -0.79 | 0.94 | 1.17 | -0.21 | 0.09 | -0.74 |
| SA composite | 5 | 29 | 0.05 | -0.91 | 0.94 | 1.15 | -0.76 | 0.28 | -0.92 |
| SA composite | 6 | 81 | 0.00 | -0.83 | 0.94 | 1.13 | -0.43 | 0.18 | -0.80 |
| PHQ-9 | 0 | 342 | 9.15 | 8.21 | 4.82 | 6.06 | -0.16 | 0.14 | -0.17 |
| PHQ-9 | 5 | 29 | 8.48 | 6.91 | 4.82 | 5.92 | -0.59 | 0.44 | -0.29 |
| PHQ-9 | 6 | 81 | 9.19 | 7.88 | 4.82 | 5.86 | -0.35 | 0.27 | -0.24 |
| SF-12 Physical | 0 | 342 | 53.34 | 51.64 | 6.98 | 8.87 | -0.17 | 0.13 | -0.21 |
| SF-12 Physical | 5 | 29 | 51.96 | 51.54 | 6.98 | 8.67 | -0.53 | 0.50 | -0.05 |
| SF-12 Physical | 6 | 81 | 51.71 | 50.85 | 6.98 | 8.64 | -0.33 | 0.29 | -0.11 |
| SF-12 Mental | 0 | 342 | 36.87 | 40.04 | 9.98 | 12.66 | -0.13 | 0.17 | 0.28 |
| SF-12 Mental | 5 | 29 | 35.72 | 42.49 | 9.98 | 12.37 | -0.36 | 0.67 | 0.60 |
| SF-12 Mental | 6 | 81 | 35.26 | 40.84 | 9.98 | 12.34 | -0.23 | 0.39 | 0.50 |
| ISMI Total (No Res) | 0 | 342 | 1.91 | 1.77 | 0.50 | 0.59 | -0.17 | 0.13 | -0.25 |
| ISMI Total (No Res) | 5 | 29 | 1.89 | 1.68 | 0.50 | 0.58 | -0.62 | 0.41 | -0.39 |
| ISMI Total (No Res) | 6 | 81 | 2.03 | 1.77 | 0.50 | 0.58 | -0.38 | 0.23 | -0.49 |
| IASMHS Total | 0 | 342 | 66.52 | 69.02 | 12.22 | 14.42 | -0.14 | 0.16 | 0.19 |
| IASMHS Total | 5 | 29 | 63.07 | 68.40 | 12.22 | 14.18 | -0.41 | 0.62 | 0.40 |
| IASMHS Total | 6 | 81 | 64.05 | 66.35 | 12.22 | 14.15 | -0.28 | 0.34 | 0.17 |
| LPFS Total | 0 | 342 | 28.59 | 27.75 | 5.82 | 6.87 | -0.16 | 0.14 | -0.13 |
| LPFS Total | 5 | 29 | 28.72 | 26.69 | 5.82 | 6.73 | -0.60 | 0.43 | -0.32 |
| LPFS Total | 6 | 81 | 29.11 | 27.71 | 5.82 | 6.72 | -0.34 | 0.27 | -0.22 |
| LPFS Self | 0 | 342 | 15.86 | 15.20 | 3.48 | 4.16 | -0.16 | 0.14 | -0.17 |
| LPFS Self | 5 | 29 | 16.17 | 14.44 | 3.48 | 4.08 | -0.63 | 0.40 | -0.46 |
| LPFS Self | 6 | 81 | 16.46 | 15.31 | 3.48 | 4.07 | -0.36 | 0.26 | -0.30 |
| LPFS Interpersonal | 0 | 342 | 12.73 | 12.54 | 3.29 | 3.95 | -0.15 | 0.15 | -0.05 |
| LPFS Interpersonal | 5 | 29 | 12.55 | 12.23 | 3.29 | 3.87 | -0.54 | 0.49 | -0.09 |
| LPFS Interpersonal | 6 | 81 | 12.65 | 12.39 | 3.29 | 3.86 | -0.32 | 0.30 | -0.07 |

Note: group 0 = preference matched; matched; group 5 = preference for anon not matched; group 6 = preference for non-anon not matched; emmean_pre = estimated mean at pre; emmean_post = estimated mean at post; SD_pre = standard deviation at pre calculated with standard error and n; SD_post = standard deviation at post calculated with standard error and n; d_lower = lower end of 95% confidence interval; d_upper = upper end of 95% confidence_interval; d = Cohen’s d

Standard deviations were derived from model-based standard errors (SD = SE × √n) rather than observed sample standard deviations, as the estimated marginal means from the mixed-effects models do not have directly corresponding observed SDs. Cohen's d was computed from these model-derived pooled SDs.

Appendix H.1

Effect sizes Cohen’s d for within-group effect sizes in the randomized arms for pre-post-followup

| outcome | group | n | emmean pre | emmean post | emmean fu | SD_pre | SD_post | SD_fu | d_lower pp | d_upper pp | d_pp | d_lower pre_fu | d_upper pre_fu | d  pre_fu | d_lower post_fu | d_upper post_fu | d  post_fu |
| --- | --- | --- | --- | --- | --- | --- | --- | --- | --- | --- | --- | --- | --- | --- | --- | --- | --- |
| SPS | 1 | 113 | 36.74 | 27.40 | 24.03 | 13.65 | 15.98 | 16.93 | -0.90 | -0.36 | -0.63 | -1.10 | -0.55 | -0.83 | -0.47 | 0.06 | -0.20 |
| SPS | 3 | 114 | 38.71 | 29.20 | 27.75 | 13.65 | 16.97 | 17.47 | -0.88 | -0.35 | -0.62 | -0.97 | -0.43 | -0.70 | -0.34 | 0.18 | -0.08 |
| SIAS | 1 | 113 | 48.32 | 38.52 | 36.31 | 13.07 | 15.46 | 16.43 | -0.95 | -0.42 | -0.68 | -1.08 | -0.54 | -0.81 | -0.40 | 0.12 | -0.14 |
| SIAS | 3 | 114 | 50.26 | 40.61 | 39.58 | 13.07 | 16.47 | 16.98 | -0.92 | -0.38 | -0.65 | -0.97 | -0.44 | -0.71 | -0.32 | 0.20 | -0.06 |
| SA composite | 1 | 113 | -0.02 | -0.81 | -1.03 | 1.01 | 1.19 | 1.26 | -0.98 | -0.44 | -0.71 | -1.16 | -0.61 | -0.89 | -0.45 | 0.08 | -0.18 |
| SA composite | 3 | 114 | 0.14 | -0.65 | -0.75 | 1.01 | 1.27 | 1.31 | -0.95 | -0.42 | -0.69 | -1.03 | -0.49 | -0.76 | -0.34 | 0.18 | -0.08 |
| PHQ-9 | 1 | 113 | 9.19 | 8.18 | 7.79 | 5.14 | 6.22 | 6.71 | -0.44 | 0.08 | -0.18 | -0.50 | 0.03 | -0.23 | -0.32 | 0.20 | -0.06 |
| PHQ-9 | 3 | 114 | 9.89 | 9.13 | 9.61 | 5.14 | 6.68 | 6.91 | -0.39 | 0.13 | -0.13 | -0.30 | 0.21 | -0.05 | -0.19 | 0.33 | 0.07 |
| SF-12 Physical | 1 | 113 | 51.78 | 50.62 | 51.96 | 7.11 | 8.95 | 9.71 | -0.40 | 0.12 | -0.14 | -0.24 | 0.28 | 0.02 | -0.12 | 0.40 | 0.14 |
| SF-12 Physical | 3 | 114 | 52.93 | 51.77 | 51.34 | 7.11 | 9.63 | 10.02 | -0.40 | 0.12 | -0.14 | -0.44 | 0.08 | -0.18 | -0.30 | 0.22 | -0.04 |
| SF-12 Mental | 1 | 113 | 35.26 | 39.91 | 39.48 | 10.39 | 12.99 | 14.07 | 0.13 | 0.66 | 0.40 | 0.08 | 0.60 | 0.34 | -0.29 | 0.23 | -0.03 |
| SF-12 Mental | 3 | 114 | 34.64 | 38.73 | 37.31 | 10.39 | 13.97 | 14.51 | 0.07 | 0.59 | 0.33 | -0.05 | 0.47 | 0.21 | -0.36 | 0.16 | -0.10 |
| ISMI Total (No Res) | 1 | 113 | 1.98 | 1.77 | 1.77 | 0.50 | 0.58 | 0.61 | -0.65 | -0.12 | -0.39 | -0.64 | -0.11 | -0.38 | -0.27 | 0.25 | -0.01 |
| ISMI Total (No Res) | 3 | 114 | 1.97 | 1.82 | 1.86 | 0.50 | 0.61 | 0.63 | -0.54 | -0.01 | -0.28 | -0.47 | 0.05 | -0.21 | -0.20 | 0.32 | 0.06 |
| IASMHS Total | 1 | 113 | 65.32 | 66.87 | 68.82 | 12.41 | 14.39 | 15.28 | -0.15 | 0.38 | 0.12 | -0.01 | 0.51 | 0.25 | -0.13 | 0.39 | 0.13 |
| IASMHS Total | 3 | 114 | 64.25 | 68.59 | 65.93 | 12.41 | 15.15 | 15.66 | 0.05 | 0.57 | 0.31 | -0.14 | 0.38 | 0.12 | -0.43 | 0.09 | -0.17 |
| LPFS Total | 1 | 113 | 28.71 | 27.27 | 26.41 | 5.75 | 6.71 | 7.13 | -0.49 | 0.03 | -0.23 | -0.62 | -0.09 | -0.35 | -0.39 | 0.14 | -0.12 |
| LPFS Total | 3 | 114 | 29.46 | 28.46 | 28.77 | 5.75 | 7.11 | 7.31 | -0.41 | 0.11 | -0.15 | -0.36 | 0.16 | -0.10 | -0.22 | 0.30 | 0.04 |
| LPFS Self | 1 | 113 | 16.23 | 15.17 | 14.46 | 3.57 | 4.16 | 4.42 | -0.54 | -0.01 | -0.27 | -0.70 | -0.18 | -0.44 | -0.43 | 0.10 | -0.16 |
| LPFS Self | 3 | 114 | 16.10 | 15.38 | 15.53 | 3.57 | 4.40 | 4.53 | -0.44 | 0.08 | -0.18 | -0.40 | 0.12 | -0.14 | -0.23 | 0.29 | 0.03 |
| LPFS Interpersonal | 1 | 113 | 12.48 | 12.11 | 11.95 | 3.19 | 3.75 | 4.00 | -0.37 | 0.16 | -0.11 | -0.41 | 0.11 | -0.15 | -0.30 | 0.22 | -0.04 |
| LPFS Interpersonal | 3 | 114 | 13.36 | 13.08 | 13.23 | 3.19 | 3.98 | 4.10 | -0.34 | 0.18 | -0.08 | -0.29 | 0.23 | -0.03 | -0.22 | 0.30 | 0.04 |

Note: group 1 = random anon; group 3 = random non-anon; emmean_pre = estimated mean at pre; emmean_post = estimated mean at post; emm fu = estimated mean at followup SD_pre = standard deviation at pre calculated with standard error and n; SD_post = standard deviation at post calculated with standard error and n; SD_fu = standard deviation at followup calculated with standard error and n; d_lower = lower end of 95% confidence interval; d_upper = upper end of 95% confidence_interval; d = Cohen’s d; pp = pre-post

Standard deviations were derived from model-based standard errors (SD = SE × √n) rather than observed sample standard deviations, as the estimated marginal means from the mixed-effects models do not have directly corresponding observed SDs. Cohen's d was computed from these model-derived pooled SDs.

Appendix H.2

Effect sizes Cohen’s d for within-group effect sizes in the preference arms for pre-post-followup

| outcome | group | n | emmean pre | emmean post | emmean fu | SD_pre | SD_post | SD_fu | d_lower pp | d_upper pp | d_pp | d_lower pre_fu | d_upper pre_fu | d pre_fu | d_lower post_fu | d_upper post_fu | d post_fu |
| --- | --- | --- | --- | --- | --- | --- | --- | --- | --- | --- | --- | --- | --- | --- | --- | --- | --- |
| SPS | 2 | 64 | 34.03 | 23.67 | 21.82 | 12.35 | 14.57 | 16.22 | -1.12 | -0.41 | -0.77 | -1.21 | -0.48 | -0.85 | -0.47 | 0.23 | -0.12 |
| SPS | 4 | 161 | 35.96 | 26.36 | 23.46 | 12.35 | 15.53 | 17.06 | -0.91 | -0.46 | -0.68 | -1.07 | -0.61 | -0.84 | -0.40 | 0.04 | -0.18 |
| SIAS | 2 | 64 | 48.94 | 40.16 | 37.79 | 11.77 | 13.71 | 15.16 | -1.04 | -0.33 | -0.69 | -1.18 | -0.46 | -0.82 | -0.51 | 0.18 | -0.16 |
| SIAS | 4 | 161 | 48.22 | 38.13 | 35.40 | 11.77 | 14.56 | 15.91 | -0.99 | -0.54 | -0.76 | -1.15 | -0.69 | -0.92 | -0.40 | 0.04 | -0.18 |
| SA composite | 2 | 64 | -0.10 | -0.88 | -1.06 | 0.89 | 1.04 | 1.15 | -1.17 | -0.45 | -0.81 | -1.30 | -0.57 | -0.94 | -0.51 | 0.19 | -0.16 |
| SA composite | 4 | 161 | -0.05 | -0.86 | -1.09 | 0.89 | 1.10 | 1.21 | -1.04 | -0.58 | -0.81 | -1.21 | -0.75 | -0.98 | -0.42 | 0.02 | -0.20 |
| PHQ-9 | 2 | 64 | 8.44 | 6.98 | 7.99 | 4.74 | 5.53 | 6.11 | -0.63 | 0.07 | -0.28 | -0.43 | 0.26 | -0.08 | -0.17 | 0.52 | 0.17 |
| PHQ-9 | 4 | 161 | 8.77 | 7.88 | 7.67 | 4.74 | 5.87 | 6.42 | -0.39 | 0.05 | -0.17 | -0.41 | 0.02 | -0.20 | -0.25 | 0.18 | -0.03 |
| SF-12 Physical | 2 | 64 | 54.20 | 52.74 | 54.23 | 6.96 | 8.23 | 9.18 | -0.54 | 0.16 | -0.19 | -0.34 | 0.35 | 0.00 | -0.18 | 0.52 | 0.17 |
| SF-12 Physical | 4 | 161 | 53.32 | 51.48 | 52.39 | 6.96 | 8.78 | 9.67 | -0.45 | -0.01 | -0.23 | -0.33 | 0.11 | -0.11 | -0.12 | 0.32 | 0.10 |
| SF-12 Mental | 2 | 64 | 39.11 | 42.88 | 41.48 | 10.02 | 11.99 | 13.47 | -0.01 | 0.69 | 0.34 | -0.15 | 0.55 | 0.20 | -0.46 | 0.24 | -0.11 |
| SF-12 Mental | 4 | 161 | 37.66 | 40.52 | 41.24 | 10.02 | 12.84 | 14.21 | 0.03 | 0.47 | 0.25 | 0.07 | 0.51 | 0.29 | -0.17 | 0.27 | 0.05 |
| ISMI Total (No Res) | 2 | 64 | 1.84 | 1.70 | 1.63 | 0.51 | 0.56 | 0.61 | -0.60 | 0.10 | -0.25 | -0.72 | -0.02 | -0.37 | -0.48 | 0.22 | -0.13 |
| ISMI Total (No Res) | 4 | 161 | 1.90 | 1.76 | 1.71 | 0.51 | 0.59 | 0.63 | -0.47 | -0.04 | -0.26 | -0.54 | -0.10 | -0.32 | -0.29 | 0.15 | -0.07 |
| IASMHS Total | 2 | 64 | 66.30 | 67.42 | 69.40 | 12.26 | 13.47 | 14.46 | -0.26 | 0.43 | 0.09 | -0.12 | 0.58 | 0.23 | -0.21 | 0.49 | 0.14 |
| IASMHS Total | 4 | 161 | 67.20 | 70.10 | 71.45 | 12.26 | 14.01 | 14.90 | 0.00 | 0.44 | 0.22 | 0.09 | 0.53 | 0.31 | -0.13 | 0.31 | 0.09 |
| LPFS Total | 2 | 64 | 27.53 | 27.03 | 26.71 | 5.97 | 6.74 | 7.32 | -0.43 | 0.27 | -0.08 | -0.47 | 0.22 | -0.12 | -0.39 | 0.30 | -0.04 |
| LPFS Total | 4 | 161 | 28.61 | 27.68 | 27.31 | 5.97 | 7.03 | 7.58 | -0.36 | 0.08 | -0.14 | -0.41 | 0.03 | -0.19 | -0.27 | 0.17 | -0.05 |
| LPFS Self | 2 | 64 | 14.81 | 14.33 | 14.51 | 3.43 | 3.95 | 4.34 | -0.48 | 0.22 | -0.13 | -0.42 | 0.27 | -0.08 | -0.30 | 0.39 | 0.04 |
| LPFS Self | 4 | 161 | 16.21 | 15.35 | 15.16 | 3.43 | 4.15 | 4.51 | -0.44 | -0.01 | -0.23 | -0.48 | -0.04 | -0.26 | -0.26 | 0.17 | -0.05 |
| LPFS Interpersonal | 2 | 64 | 12.72 | 12.70 | 12.19 | 3.38 | 3.89 | 4.26 | -0.35 | 0.34 | -0.01 | -0.48 | 0.21 | -0.14 | -0.47 | 0.22 | -0.12 |
| LPFS Interpersonal | 4 | 161 | 12.40 | 12.29 | 12.12 | 3.38 | 4.07 | 4.43 | -0.25 | 0.19 | -0.03 | -0.29 | 0.15 | -0.07 | -0.26 | 0.18 | -0.04 |

Note: group 2 = prefer anon; group 4 = prefer non-anon; emmean_pre = estimated mean at pre; emmean_post = estimated mean at post; emm fu = estimated mean at followup SD_pre = standard deviation at pre calculated with standard error and n; SD_post = standard deviation at post calculated with standard error and n; SD_fu = standard deviation at followup calculated with standard error and n; d_lower = lower end of 95% confidence interval; d_upper = upper end of 95% confidence_interval; d = Cohen’s d; pp = pre-post

Standard deviations were derived from model-based standard errors (SD = SE × √n) rather than observed sample standard deviations, as the estimated marginal means from the mixed-effects models do not have directly corresponding observed SDs. Cohen's d was computed from these model-derived pooled SDs.

Appendix H.3

Effect sizes Cohen’s d for within-group effect sizes in the preference matched vs. not matched conditions for pre-post-followup

| outcome | group | n | emmean pre | emmean post | emmean fu | SD_pre | SD_post | SD_fu | d_lower pp | d_upper pp | d_pp | d_lower pre_fu | d_upper pre_fu | d pre_fu | d_lower post_fu | d_upper post_fu | d post_fu |
| --- | --- | --- | --- | --- | --- | --- | --- | --- | --- | --- | --- | --- | --- | --- | --- | --- | --- |
| SPS | 0 | 342 | 36.31 | 27.03 | 24.67 | 13.12 | 16.02 | 17.33 | -0.79 | -0.48 | -0.63 | -0.91 | -0.60 | -0.76 | -0.29 | 0.01 | -0.14 |
| SPS | 1 | 110 | 37.40 | 26.82 | 24.09 | 13.12 | 15.54 | 16.19 | -1.01 | -0.46 | -0.74 | -1.18 | -0.62 | -0.90 | -0.44 | 0.09 | -0.17 |
| SIAS | 0 | 342 | 48.97 | 39.53 | 37.57 | 12.45 | 15.24 | 16.50 | -0.83 | -0.52 | -0.68 | -0.94 | -0.62 | -0.78 | -0.27 | 0.03 | -0.12 |
| SIAS | 1 | 110 | 48.52 | 38.13 | 35.69 | 12.45 | 14.78 | 15.41 | -1.03 | -0.49 | -0.76 | -1.19 | -0.64 | -0.92 | -0.43 | 0.10 | -0.16 |
| SA composite | 0 | 342 | -0.01 | -0.78 | -0.95 | 0.96 | 1.17 | 1.26 | -0.88 | -0.57 | -0.72 | -1.00 | -0.69 | -0.85 | -0.30 | 0.00 | -0.15 |
| SA composite | 1 | 110 | 0.02 | -0.85 | -1.06 | 0.96 | 1.13 | 1.18 | -1.10 | -0.55 | -0.82 | -1.28 | -0.72 | -1.00 | -0.45 | 0.08 | -0.18 |
| PHQ-9 | 0 | 342 | 9.15 | 8.31 | 8.66 | 4.96 | 6.16 | 6.70 | -0.30 | 0.00 | -0.15 | -0.23 | 0.07 | -0.08 | -0.09 | 0.20 | 0.05 |
| PHQ-9 | 1 | 110 | 9.00 | 7.61 | 7.12 | 4.96 | 5.97 | 6.28 | -0.52 | 0.01 | -0.25 | -0.60 | -0.07 | -0.33 | -0.34 | 0.18 | -0.08 |
| SF-12 Physical | 0 | 342 | 53.34 | 51.60 | 52.74 | 7.02 | 9.00 | 9.89 | -0.37 | -0.06 | -0.22 | -0.22 | 0.08 | -0.07 | -0.03 | 0.27 | 0.12 |
| SF-12 Physical | 1 | 110 | 51.77 | 51.10 | 50.95 | 7.02 | 8.74 | 9.20 | -0.35 | 0.18 | -0.09 | -0.36 | 0.16 | -0.10 | -0.28 | 0.25 | -0.02 |
| SF-12 Mental | 0 | 342 | 36.87 | 39.95 | 39.13 | 10.29 | 13.13 | 14.43 | 0.11 | 0.41 | 0.26 | 0.03 | 0.33 | 0.18 | -0.21 | 0.09 | -0.06 |
| SF-12 Mental | 1 | 110 | 35.38 | 41.11 | 41.51 | 10.29 | 12.77 | 13.43 | 0.23 | 0.76 | 0.49 | 0.24 | 0.78 | 0.51 | -0.23 | 0.29 | 0.03 |
| ISMI Total (No Res) | 0 | 342 | 1.91 | 1.78 | 1.76 | 0.50 | 0.59 | 0.63 | -0.39 | -0.09 | -0.24 | -0.42 | -0.11 | -0.27 | -0.19 | 0.11 | -0.04 |
| ISMI Total (No Res) | 1 | 110 | 1.99 | 1.75 | 1.75 | 0.50 | 0.58 | 0.60 | -0.73 | -0.19 | -0.46 | -0.71 | -0.18 | -0.45 | -0.26 | 0.27 | 0.00 |
| IASMHS Total | 0 | 342 | 66.52 | 68.88 | 69.42 | 12.35 | 14.40 | 15.39 | 0.03 | 0.33 | 0.18 | 0.06 | 0.36 | 0.21 | -0.11 | 0.19 | 0.04 |
| IASMHS Total | 1 | 110 | 63.79 | 67.02 | 67.90 | 12.35 | 14.13 | 14.73 | -0.02 | 0.51 | 0.24 | 0.04 | 0.57 | 0.30 | -0.20 | 0.33 | 0.06 |
| LPFS Total | 0 | 342 | 28.59 | 27.80 | 27.57 | 5.88 | 6.97 | 7.46 | -0.27 | 0.03 | -0.12 | -0.30 | 0.00 | -0.15 | -0.18 | 0.12 | -0.03 |
| LPFS Total | 1 | 110 | 29.01 | 27.35 | 26.80 | 5.88 | 6.80 | 7.12 | -0.53 | 0.00 | -0.26 | -0.60 | -0.07 | -0.34 | -0.34 | 0.19 | -0.08 |
| LPFS Self | 0 | 342 | 15.86 | 15.22 | 15.13 | 3.52 | 4.21 | 4.53 | -0.32 | -0.02 | -0.17 | -0.33 | -0.03 | -0.18 | -0.17 | 0.13 | -0.02 |
| LPFS Self | 1 | 110 | 16.38 | 15.04 | 14.60 | 3.52 | 4.11 | 4.31 | -0.62 | -0.08 | -0.35 | -0.72 | -0.19 | -0.45 | -0.37 | 0.16 | -0.10 |
| LPFS Interpersonal | 0 | 342 | 12.73 | 12.57 | 12.43 | 3.30 | 3.97 | 4.28 | -0.19 | 0.11 | -0.04 | -0.23 | 0.07 | -0.08 | -0.18 | 0.12 | -0.03 |
| LPFS Interpersonal | 1 | 110 | 12.63 | 12.30 | 12.18 | 3.30 | 3.87 | 4.07 | -0.35 | 0.17 | -0.09 | -0.39 | 0.14 | -0.12 | -0.30 | 0.23 | -0.03 |

Note: group 0 = prefererence matched; group 1 = preference not matched; emmean_pre = estimated mean at pre; emmean_post = estimated mean at post; emm fu = estimated mean at followup SD_pre = standard deviation at pre calculated with standard error and n; SD_post = standard deviation at post calculated with standard error and n; SD_fu = standard deviation at followup calculated with standard error and n; d_lower = lower end of 95% confidence interval; d_upper = upper end of 95% confidence_interval; d = Cohen’s d; pp = pre-post

Standard deviations were derived from model-based standard errors (SD = SE × √n) rather than observed sample standard deviations, as the estimated marginal means from the mixed-effects models do not have directly corresponding observed SDs. Cohen's d was computed from these model-derived pooled SDs.

Appendix H.4

Effect sizes Cohen’s d for within-group effect sizes in the preference matched vs. not matched detailed conditions for pre-post-followup

| outcome | group | n | emmean pre | emmean post | emmean fu | SD_pre | SD_post | SD_fu | d_lower pp | d_upper pp | d_pp | d_lower pre_fu | d_upper pre_fu | d pre_fu | d_lower post_fu | d_upper post_fu | d post_fu |
| --- | --- | --- | --- | --- | --- | --- | --- | --- | --- | --- | --- | --- | --- | --- | --- | --- | --- |
| SPS | 0 | 342 | 36.31 | 27.03 | 24.67 | 13.13 | 16.04 | 17.35 | -0.20 | 0.10 | -0.63 | -0.21 | 0.09 | -0.76 | -0.16 | 0.14 | -0.14 |
| SPS | 5 | 29 | 37.93 | 26.40 | 25.02 | 13.13 | 15.63 | 15.63 | -0.72 | 0.31 | -0.80 | -0.75 | 0.28 | -0.89 | -0.54 | 0.49 | -0.09 |
| SPS | 6 | 81 | 37.21 | 26.95 | 23.71 | 13.13 | 15.54 | 16.45 | -0.42 | 0.20 | -0.71 | -0.45 | 0.17 | -0.91 | -0.34 | 0.28 | -0.20 |
| SIAS | 0 | 342 | 48.97 | 39.53 | 37.57 | 12.47 | 15.26 | 16.52 | -0.20 | 0.10 | -0.68 | -0.21 | 0.09 | -0.78 | -0.16 | 0.14 | -0.12 |
| SIAS | 5 | 29 | 48.90 | 38.08 | 35.31 | 12.47 | 14.87 | 14.87 | -0.72 | 0.31 | -0.79 | -0.78 | 0.26 | -0.99 | -0.56 | 0.47 | -0.19 |
| SIAS | 6 | 81 | 48.38 | 38.16 | 35.86 | 12.47 | 14.79 | 15.66 | -0.43 | 0.19 | -0.75 | -0.45 | 0.17 | -0.88 | -0.33 | 0.28 | -0.15 |
| SA composite | 0 | 342 | -0.01 | -0.78 | -0.95 | 0.96 | 1.17 | 1.26 | -0.21 | 0.09 | -0.72 | -0.21 | 0.09 | -0.85 | -0.16 | 0.14 | -0.15 |
| SA composite | 5 | 29 | 0.05 | -0.86 | -1.04 | 0.96 | 1.14 | 1.14 | -0.74 | 0.29 | -0.87 | -0.79 | 0.25 | -1.04 | -0.55 | 0.47 | -0.15 |
| SA composite | 6 | 81 | 0.00 | -0.84 | -1.06 | 0.96 | 1.13 | 1.20 | -0.43 | 0.18 | -0.80 | -0.46 | 0.15 | -0.98 | -0.34 | 0.28 | -0.19 |
| PHQ-9 | 0 | 342 | 9.15 | 8.31 | 8.66 | 4.97 | 6.17 | 6.71 | -0.16 | 0.14 | -0.15 | -0.16 | 0.14 | -0.08 | -0.15 | 0.15 | 0.05 |
| PHQ-9 | 5 | 29 | 8.48 | 7.00 | 7.05 | 4.97 | 6.00 | 6.00 | -0.59 | 0.44 | -0.27 | -0.58 | 0.45 | -0.26 | -0.51 | 0.52 | 0.01 |
| PHQ-9 | 6 | 81 | 9.19 | 7.83 | 7.12 | 4.97 | 5.97 | 6.40 | -0.35 | 0.27 | -0.25 | -0.36 | 0.25 | -0.36 | -0.33 | 0.29 | -0.11 |
| SF-12 Physical | 0 | 342 | 53.34 | 51.60 | 52.74 | 7.03 | 9.01 | 9.91 | -0.17 | 0.13 | -0.21 | -0.16 | 0.14 | -0.07 | -0.14 | 0.16 | 0.12 |
| SF-12 Physical | 5 | 29 | 51.96 | 51.53 | 51.03 | 7.03 | 8.75 | 8.75 | -0.53 | 0.50 | -0.05 | -0.55 | 0.48 | -0.12 | -0.53 | 0.50 | -0.06 |
| SF-12 Physical | 6 | 81 | 51.71 | 50.94 | 50.92 | 7.03 | 8.76 | 9.41 | -0.32 | 0.29 | -0.10 | -0.32 | 0.29 | -0.09 | -0.31 | 0.31 | 0.00 |
| SF-12 Mental | 0 | 342 | 36.87 | 39.95 | 39.13 | 10.30 | 13.15 | 14.45 | -0.13 | 0.17 | 0.26 | -0.14 | 0.16 | 0.18 | -0.15 | 0.15 | -0.06 |
| SF-12 Mental | 5 | 29 | 35.72 | 41.94 | 42.71 | 10.30 | 12.78 | 12.78 | -0.38 | 0.66 | 0.54 | -0.36 | 0.67 | 0.60 | -0.50 | 0.53 | 0.06 |
| SF-12 Mental | 6 | 81 | 35.26 | 40.80 | 41.01 | 10.30 | 12.79 | 13.73 | -0.23 | 0.38 | 0.48 | -0.23 | 0.38 | 0.47 | -0.31 | 0.31 | 0.02 |
| ISMI Total (No Res) | 0 | 342 | 1.91 | 1.78 | 1.76 | 0.50 | 0.59 | 0.63 | -0.17 | 0.13 | -0.24 | -0.17 | 0.13 | -0.27 | -0.15 | 0.15 | -0.04 |
| ISMI Total (No Res) | 5 | 29 | 1.89 | 1.69 | 1.73 | 0.50 | 0.58 | 0.58 | -0.61 | 0.42 | -0.38 | -0.59 | 0.44 | -0.30 | -0.50 | 0.53 | 0.07 |
| ISMI Total (No Res) | 6 | 81 | 2.03 | 1.77 | 1.75 | 0.50 | 0.58 | 0.61 | -0.38 | 0.23 | -0.49 | -0.39 | 0.23 | -0.50 | -0.31 | 0.30 | -0.03 |
| IASMHS Total | 0 | 342 | 66.52 | 68.88 | 69.42 | 12.36 | 14.41 | 15.39 | -0.14 | 0.16 | 0.18 | -0.13 | 0.17 | 0.21 | -0.15 | 0.15 | 0.04 |
| IASMHS Total | 5 | 29 | 63.07 | 68.58 | 66.30 | 12.36 | 14.14 | 14.34 | -0.41 | 0.62 | 0.41 | -0.45 | 0.58 | 0.24 | -0.56 | 0.47 | -0.16 |
| IASMHS Total | 6 | 81 | 64.05 | 66.47 | 68.58 | 12.36 | 14.13 | 14.90 | -0.28 | 0.34 | 0.18 | -0.26 | 0.36 | 0.33 | -0.29 | 0.33 | 0.15 |
| LPFS Total | 0 | 342 | 28.59 | 27.80 | 27.57 | 5.88 | 6.97 | 7.46 | -0.16 | 0.14 | -0.12 | -0.16 | 0.14 | -0.15 | -0.15 | 0.15 | -0.03 |
| LPFS Total | 5 | 29 | 28.72 | 26.61 | 27.77 | 5.88 | 6.81 | 6.92 | -0.60 | 0.43 | -0.33 | -0.55 | 0.48 | -0.15 | -0.47 | 0.56 | 0.17 |
| LPFS Total | 6 | 81 | 29.11 | 27.61 | 26.38 | 5.88 | 6.80 | 7.20 | -0.34 | 0.27 | -0.24 | -0.37 | 0.24 | -0.42 | -0.34 | 0.28 | -0.18 |
| LPFS Self | 0 | 342 | 15.86 | 15.22 | 15.13 | 3.52 | 4.22 | 4.53 | -0.16 | 0.14 | -0.17 | -0.16 | 0.14 | -0.18 | -0.15 | 0.15 | -0.02 |
| LPFS Self | 5 | 29 | 16.17 | 14.37 | 14.85 | 3.52 | 4.11 | 4.18 | -0.64 | 0.39 | -0.47 | -0.60 | 0.43 | -0.34 | -0.48 | 0.55 | 0.12 |
| LPFS Self | 6 | 81 | 16.46 | 15.28 | 14.47 | 3.52 | 4.11 | 4.37 | -0.36 | 0.26 | -0.31 | -0.39 | 0.23 | -0.50 | -0.34 | 0.28 | -0.19 |
| LPFS Interpersonal | 0 | 342 | 12.73 | 12.57 | 12.43 | 3.31 | 3.97 | 4.28 | -0.15 | 0.15 | -0.04 | -0.16 | 0.14 | -0.08 | -0.15 | 0.15 | -0.03 |
| LPFS Interpersonal | 5 | 29 | 12.55 | 12.21 | 12.87 | 3.31 | 3.88 | 3.94 | -0.54 | 0.49 | -0.09 | -0.49 | 0.54 | 0.09 | -0.47 | 0.56 | 0.17 |
| LPFS Interpersonal | 6 | 81 | 12.65 | 12.33 | 11.89 | 3.31 | 3.87 | 4.12 | -0.32 | 0.29 | -0.09 | -0.34 | 0.28 | -0.21 | -0.33 | 0.29 | -0.11 |

Note: group 0 = prefererence matched; matched; group 5 = preference for anon not matched; group 6 = preference for non-anon not matched; emmean_pre = estimated mean at pre; emmean_post = estimated mean at post; emm fu = estimated mean at followup SD_pre = standard deviation at pre calculated with standard error and n; SD_post = standard deviation at post calculated with standard error and n; SD_fu = standard deviation at followup calculated with standard error and n; d_lower = lower end of 95% confidence interval; d_upper = upper end of 95% confidence_interval; d = Cohen’s d; pp = pre-post

Standard deviations were derived from model-based standard errors (SD = SE × √n) rather than observed sample standard deviations, as the estimated marginal means from the mixed-effects models do not have directly corresponding observed SDs. Cohen's d was computed from these model-derived pooled SDs.

Appendix I.1

Preference changes from baseline to subsequent timepoints in the randomized and preference arms

| Change pre to mid1 | |  |  |  |  |  |
| --- | --- | --- | --- | --- | --- | --- |
| **Condition** | **0** | **1** | **2** | **3** | **4** | **88** |
| **Random anon** | 41 | 4 | 23 | 3 | 13 | 29 |
| **Preference anon** | 41 | 5 | 0 | 4 | 0 | 14 |
| **Random non-anon** | 56 | 2 | 7 | 4 | 3 | 42 |
| **Preference non-anon** | 101 | 0 | 12 | 0 | 9 | 39 |
|  |  |  |  |  |  |  |
| Change pre to mid2 | |  |  |  |  |  |
| **Condition** | **0** | **1** | **2** | **3** | **4** | **88** |
| **Random anon** | 34 | 2 | 21 | 2 | 10 | 44 |
| **Preference anon** | 30 | 6 | 0 | 4 | 0 | 24 |
| **Random non-anon** | 46 | 6 | 5 | 1 | 3 | 53 |
| **Preference non-anon** | 69 | 0 | 11 | 0 | 8 | 73 |
|  |  |  |  |  |  |  |
| Change pre to post | |  |  |  |  |  |
| **Condition** | **0** | **1** | **2** | **3** | **4** | **88** |
| **Random anon** | 33 | 2 | 14 | 3 | 12 | 49 |
| **Preference anon** | 27 | 6 | 0 | 5 | 0 | 26 |
| **Random non-anon** | 35 | 6 | 5 | 0 | 7 | 61 |
| **Preference non-anon** | 62 | 0 | 13 | 0 | 6 | 80 |
|  |  |  |  |  |  |  |
| Change pre to followup | |  |  |  |  |  |
| **Condition** | **0** | **1** | **2** | **3** | **4** | **88** |
| **Random anon** | 25 | 0 | 15 | 1 | 9 | 88 |
| **Preference anon** | 36 | 1 | 0 | 3 | 0 | 37 |
| **Random non-anon** | 23 | 4 | 2 | 3 | 2 | 67 |
| **Preference non-anon** | 44 | 0 | 10 | 0 | 7 | 100 |

Note: 0 = no change, 1 = change from anon to non-anon, 2 = change from non-anon to anon, 3 = change from anon to no preference, 4 = change from non-anon to no Preference, 88 = no value possible due to missings

Appendix I.2

Preference changes from baseline to subsequent timepoints in the whole sample split by preference match vs. no match

| Change pre to mid1 | |  |  |  |  |  |
| --- | --- | --- | --- | --- | --- | --- |
| **Condition** | **0** | **1** | **2** | **3** | **4** | **88** |
| **Match** | 203 | 9 | 19 | 7 | 12 | 92 |
| **No match** | 36 | 2 | 23 | 4 | 13 | 32 |
|  |  |  |  |  |  |  |
| Change pre to mid2 | |  |  |  |  |  |
| **Condition** | **0** | **1** | **2** | **3** | **4** | **88** |
| **Match** | 150 | 8 | 16 | 6 | 11 | 151 |
| **No match** | 29 | 6 | 21 | 1 | 10 | 43 |
|  |  |  |  |  |  |  |
| Change pre to post | |  |  |  |  |  |
| **Condition** | **0** | **1** | **2** | **3** | **4** | **88** |
| **Match** | 128 | 8 | 18 | 8 | 13 | 167 |
| **No match** | 29 | 6 | 14 | 0 | 12 | 49 |
|  |  |  |  |  |  |  |
| Change pre to followup | |  |  |  |  |  |
| **Condition** | **0** | **1** | **2** | **3** | **4** | **88** |
| **Match** | 109 | 1 | 12 | 4 | 9 | 207 |
| **No match** | 19 | 4 | 15 | 3 | 9 | 60 |

Note: 0 = no change, 1 = change from anon to non-anon, 2 = change from non-anon to anon, 3 = change from anon to no preference, 4 = change from non-anon to no Preference, 88 = no value possible due to missings

Appendix J.1

Pooled mixed-effects model results for the primary outcome measures under multiple imputation (missing at random), randomized arm

| Outcome | Parameter | Estimate | SE | t | df | p | FMI |
| --- | --- | --- | --- | --- | --- | --- | --- |
| Composite score | Intercept (JOURNeY_A, pre) | -0.017 | 0.09 | -0.19 | >10000 | 0.848 | 0 |
| Composite score | Time (pre to post, JOURNeY_A) | -0.7 | 0.115 | -6.07 | 250.9 | <.001 | 0.28 |
| Composite score | Version (JOURNeY_NonA vs. A, pre) | 0.161 | 0.127 | 1.27 | >10000 | 0.204 | 0 |
| Composite score | Time x Version | -0.142 | 0.164 | -0.87 | 231.6 | 0.387 | 0.29 |
| SPS | Intercept (JOURNeY_A, pre) | 36.743 | 1.267 | 29 | >10000 | <.001 | 0 |
| SPS | Time (pre to post, JOURNeY_A) | -8.5 | 1.716 | -4.95 | 231.9 | <.001 | 0.29 |
| SPS | Version (JOURNeY_NonA vs. A, pre) | 1.967 | 1.788 | 1.1 | >10000 | 0.271 | 0 |
| SPS | Time x Version | -1.9 | 2.448 | -0.78 | 208.6 | 0.439 | 0.31 |
| SIAS | Intercept (JOURNeY_A, pre) | 48.319 | 1.238 | 39.03 | >10000 | <.001 | 0 |
| SIAS | Time (pre to post, JOURNeY_A) | -8.519 | 1.737 | -4.91 | 172.7 | <.001 | 0.34 |
| SIAS | Version (JOURNeY_NonA vs. A, pre) | 1.945 | 1.747 | 1.11 | >10000 | 0.266 | 0 |
| SIAS | Time x Version | -1.551 | 2.415 | -0.64 | 195.3 | 0.522 | 0.32 |

Note. Results are pooled across m = 20 imputed datasets (predictive mean matching) using Rubin's rules, from linear mixed-effects models of the form outcome ~ time × version + (1 | participant), estimated for the pre-to-post interval. The reference category is JOURNeY_A (anonymous) at pre-treatment; "Time × Version" is the time × program version interaction, the confirmatory test of superiority. SE = standard error; df = degrees of freedom; FMI = fraction of missing information. SPS = Social Phobia Scale; SIAS = Social Interaction Anxiety Scale; composite = mean of the z-standardized SPS and SIAS scores. This imputation model included only the primary outcome variables.

Appendix J.2

Pooled mixed-effects model for the composite score under the auxiliary-enriched imputation model (δ = 0), randomized arm

| Outcome | Parameter | Estimate | SE | t | df | p | FMI |
| --- | --- | --- | --- | --- | --- | --- | --- |
| Composite score (auxiliary model) | Intercept (JOURNeY_A, pre) | -0.017 | 0.097 | -0.18 | >10000 | 0.859 | 0 |
| Composite score (auxiliary model) | Time (pre to post, JOURNeY_A) | -0.724 | 0.109 | -6.62 | 136.3 | <.001 | 0.38 |
| Composite score (auxiliary model) | Version (JOURNeY_NonA vs. A, pre) | 0.161 | 0.137 | 1.18 | >10000 | 0.239 | 0 |
| Composite score (auxiliary model) | Time x Version | -0.032 | 0.14 | -0.23 | 345.8 | 0.816 | 0.24 |

Note. As Table J1, but the imputation model was enriched with auxiliary variables predictive of the missing values or of missingness (follow-up primary outcome score, mid-treatment measures serving solely as auxiliary predictors, and baseline covariates), and no shift was applied to imputed values (δ = 0). This model serves as the reference point for the delta-adjusted analysis in Table J.3. The reduced fraction of missing information for the interaction (FMI = .24), relative to the corresponding model in Table J.1, reflects the information recovered by the auxiliary variables. Abbreviations as in Table J.1.

Appendix J.3

Delta-adjusted (tipping-point) sensitivity analysis for the primary outcome (composite score), randomized arm

| Scenario | delta | Estimate | SE | p |
| --- | --- | --- | --- | --- |
| JOURNeY_A non-completers worse | 0 | -0.032 | 0.14 | 0.816 |
| JOURNeY_A non-completers worse | 0.1 | -0.075 | 0.14 | 0.593 |
| JOURNeY_A non-completers worse | 0.2 | -0.117 | 0.141 | 0.405 |
| JOURNeY_A non-completers worse | 0.3 | -0.16 | 0.141 | 0.259 |
| JOURNeY_A non-completers worse | 0.4 | -0.202 | 0.142 | 0.156 |
| JOURNeY_A non-completers worse | 0.5 | -0.245 | 0.143 | 0.088 |
| JOURNeY_A non-completers worse | 0.6 | -0.287 | 0.144 | 0.047 |
| JOURNeY_A non-completers worse | 0.7 | -0.33 | 0.146 | 0.024 |
| JOURNeY_A non-completers worse | 0.8 | -0.372 | 0.147 | 0.012 |
| JOURNeY_A non-completers worse | 0.9 | -0.415 | 0.149 | 0.006 |
| JOURNeY_A non-completers worse | 1 | -0.457 | 0.15 | 0.003 |
| JOURNeY_A non-completers worse | 1.1 | -0.5 | 0.152 | 0.001 |
| JOURNeY_A non-completers worse | 1.2 | -0.542 | 0.154 | <.001 |
| JOURNeY_A non-completers worse | 1.3 | -0.585 | 0.156 | <.001 |
| JOURNeY_A non-completers worse | 1.4 | -0.627 | 0.158 | <.001 |
| JOURNeY_A non-completers worse | 1.5 | -0.67 | 0.161 | <.001 |
| JOURNeY_NonA non-completers worse | 0 | -0.032 | 0.14 | 0.816 |
| JOURNeY_NonA non-completers worse | 0.1 | 0.02 | 0.14 | 0.885 |
| JOURNeY_NonA non-completers worse | 0.2 | 0.073 | 0.14 | 0.604 |
| JOURNeY_NonA non-completers worse | 0.3 | 0.125 | 0.141 | 0.374 |
| JOURNeY_NonA non-completers worse | 0.4 | 0.178 | 0.142 | 0.209 |
| JOURNeY_NonA non-completers worse | 0.5 | 0.231 | 0.143 | 0.106 |
| JOURNeY_NonA non-completers worse | 0.6 | 0.283 | 0.144 | 0.049 |
| JOURNeY_NonA non-completers worse | 0.7 | 0.336 | 0.145 | 0.021 |
| JOURNeY_NonA non-completers worse | 0.8 | 0.389 | 0.146 | 0.008 |
| JOURNeY_NonA non-completers worse | 0.9 | 0.441 | 0.148 | 0.003 |
| JOURNeY_NonA non-completers worse | 1 | 0.494 | 0.149 | 0.001 |
| JOURNeY_NonA non-completers worse | 1.1 | 0.547 | 0.151 | <.001 |
| JOURNeY_NonA non-completers worse | 1.2 | 0.599 | 0.153 | <.001 |
| JOURNeY_NonA non-completers worse | 1.3 | 0.652 | 0.155 | <.001 |
| JOURNeY_NonA non-completers worse | 1.4 | 0.704 | 0.157 | <.001 |
| JOURNeY_NonA non-completers worse | 1.5 | 0.757 | 0.159 | <.001 |

Note. Each row reports the pooled time × version interaction (estimate, SE = standard error, and p-value across m = 20 imputed datasets) after shifting the imputed post-treatment values of non-completers in the indicated condition by δ standard deviation units toward greater symptom severity. δ is expressed in the metric of the composite score, standardized to the pre-treatment mean and standard deviation of the full sample. The two scenarios shift non-completers in the anonymous (JOURNeY_A) and non-anonymous (JOURNeY_NonA) conditions, respectively.

Appendix J.4

Figure.

Tipping-point analysis for the primary outcome (composite score) in the randomized arm

Note: Pooled p-values of the time × version interaction as a function of δ, the shift (in pre-treatment SD units, toward greater severity) applied to imputed post-treatment values of non-completers in the anonymous condition. The dashed line marks p = .05; the interaction becomes significant at δ ≥ 0.6.
